# Condition‐Associated Pattern Extraction and Recovery From Multi‐Condition Single‐Cell RNA‐seq Data With CAPER

**DOI:** 10.1002/advs.76186

**Published:** 2026-06-22

**Authors:** Ye Li, Jin Ning, An Wang, Minxi Shi, Yuanze Chen, Guoliang Liu, Shiquan Sun

**Affiliations:** ^1^ Center For Single‐Cell Omics and Health School of Public Health Xi'an Jiaotong University Xi'an Shaanxi P. R. China; ^2^ Collaborative Innovation Center of Endemic Diseases and Health Promotion in Silk Road Region and NHC Key Laboratory of Environment and Endemic Diseases Xi'an Jiaotong University Xi'an Shaanxi P. R. China; ^3^ Sun Yat‐Sen Memorial Hospital Sun Yat‐Sen University Guangzhou Guangdong P. R. China; ^4^ Key Laboratory of Environment and Genes Related to Diseases (Xi'an Jiaotong University) Ministry of Education Xi'an Shaanxi P. R. China; ^5^ Key Laboratory for Disease Prevention and Control and Health Promotion of Shaanxi Province Xi'an Shaanxi P. R. China

**Keywords:** batch correction, biological signal disentanglement, cell‐population‐specific response, multi‐condition scRNA‐seq data, reconstructed gene expression matrix

## Abstract

A central challenge in multi‐condition single‐cell RNA sequencing (scRNA‐seq) data analysis is the disentanglement of true biological signals from unwanted variations in complex experimental designs. Current statistical and machine learning‐based methods struggle with this task, often providing only visualizable embeddings, over‐correcting and discarding biological signal, or failing to resolve cell‐type‐specific responses. Here, we present CAPER, a matrix factorization framework that explicitly disentangles shared biological states from condition‐specific variations. CAPER directly outputs an interpretable, batch‐corrected expression matrix in which the signal of interest is preserved and isolated. The performance of CAPER is validated using extensive simulations, followed by three real‐world multi‐condition scRNA‐seq data applications, representing distinct signal‐to‐noise ratio (SNR) scenarios: a controlled immune stimulation in PBMCs with high SNR, a tumor‐microenvironment dataset from LUAD with confounded SNR, and a complex autoimmune disease dataset from T1D with low SNR. Across these settings, CAPER yields interpretable latent factors linked to relevant biology, accurately recovers key differentially expressed genes, and correctly identifies the most responsive cell populations. CAPER is a robust and interpretable tool for recovering biological signals from multi‐condition single‐cell RNA‐seq data, enabling reliable discovery in disease research and functional genomics.

## Graphical abstract

Decoupling biological signals from unwanted variation in multi‑condition single‑cell RNA sequencing data remains challenging. CAPER disentangles condition‑associated biological effects from sample heterogeneity through matrix factorization, producing interpretable latent factors and a batch‑corrected expression matrix. Across high, confounded, and low signal‑to‑noise ratio regimes, CAPER robustly recovers true biological signals, captures cell‑population heterogeneity, and enables reliable downstream analyses.

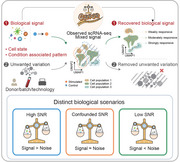

## 1 Introduction

Single‐cell RNA sequencing (scRNA‐seq) has revolutionized our ability to characterize cellular heterogeneity and dynamic responses in development, disease, and perturbation experiments [1, 2]. A growing number of studies involve multi‐condition designs, e.g., comparing control and diseased tissue or stimulated and control cells, to identify the cellular and molecular responses of biological processes [3, 4, 5]. An analytical bottleneck, however, is the isolation of these condition‐specific and/or cell‐type‐specific signals from confounding variations, including batch effects, donor differences, and technical variation [5, 6].

Downstream analysis of multi‐condition single‐cell data, such as differential expression [7, 8] and trajectory inference [9], ideally operates on a corrected expression matrix from which unwanted variation has been removed while biological signals of interest are preserved. Existing computational strategies, e.g., integrative analysis [10] or batch correction analysis [5], have limitations in achieving this balance. Methods like Harmony [11], BBKNN [12], or the variational autoencoder (VAE)‐based tools (e.g., SCALEX [13]) output low‐dimensional embeddings or neighborhood graphs designed for visualization and clustering, but they do not provide a corrected gene expression matrix. Other approaches, such as scVI [14], scGen [15], ComBat [16], and fastMNN [17], do produce corrected, predicted, or model‐derived expression data, yet their underlying model assumptions may restrict their ability to recover condition‐associated signals. More recently, a statistical method CellANOVA [18], was specifically designed for multi‑condition scRNA‑seq data; however, it enforces strict orthogonality between biological and technical factors, risking the loss of true biological signal. Meanwhile, deep learning approaches such as scDisInFact [19] can over‐integrate data, homogenizing critical cell‐type‐specific response patterns.

To address these shortcomings, we present CAPER, a flexible and statistical matrix factorization framework to extract condition‐associated patterns. Specifically, CAPER explicitly models two sources of variations: shared latent factors that capture core cell identity and condition‐associated biological signals, and condition‐specific latent factors that capture residual unwanted variation unique to each condition. This design allows CAPER to output an interpretable, batch‐corrected expression matrix in which the signal of interest is both isolated and preserved.

We demonstrate CAPER's performance through extensive simulations and three real‐world applications of multi‐condition scRNA‐seq data, with decreasing signal‐to‐noise ratio (SNR) scenarios, i.e., a controlled cytokine stimulation of peripheral blood mononuclear cells (PBMCs) with high SNR, a tumor‐vs.‐control comparison in lung adenocarcinoma (LUAD) with confounded SNR, and a case‐control study in type 1 diabetes (T1D) with low SNR. In each case, CAPER provides interpretable latent factors linked to relevant biology, correctly identifies the most responsive cell populations, accurately recovers cell‐type‐specific differentially expressed genes, and supports other downstream analyses such as gene co‐expression analysis, trajectory inference, and clustering. CAPER thus provides a robust, interpretable solution for recovering biological signals from multi‐condition single‐cell data, facilitating more reliable downstream analysis and discovery.

## 2 Results

### 2.1 Overview of CAPER

**FIGURE 1:**
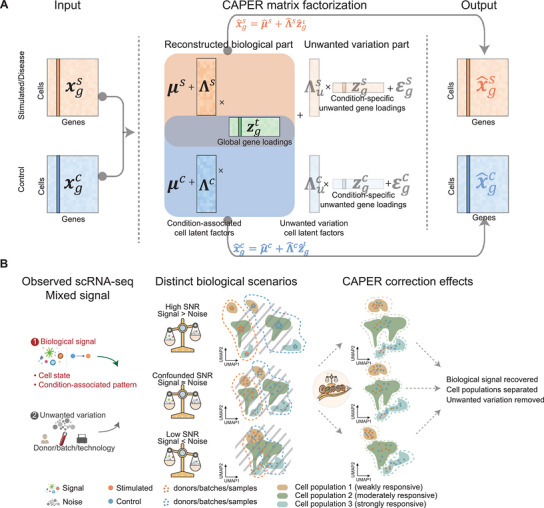
Overview of the CAPER framework. (A) CAPER is a statistical matrix‐factorization framework designed to disentangle true biological signals from unwanted technical and sample‐to‐sample variation. The method takes as input scaled, normalized gene expression matrices from single‐cell experiments spanning multiple conditions. Through its factorization approach, CAPER explicitly separates biological states from condition‐specific variations, outputting two key products: (1) interpretable latent factors that capture shared cell‐state structure and reproducible condition‐associated biological effects, and (2) a corrected gene expression matrix in which the signal of interest is preserved and isolated for robust downstream analysis. (B) The schematic illustrates CAPER's ability to recover biological structure across different signal‐to‐noise regimes: (1) High SNR; (2) Confounded SNR; (3) Low SNR. In the UMAP embedding, weakly responsive cell populations remain nearly overlapped between conditions, moderately responsive populations remain partially overlapped, and strongly responsive populations become nearly separated.

CAPER is primarily built on a statistical matrix‐factorization (MF) framework that exploits a two‐condition (i.e., control and perturbation) experimental design to explicitly identify biological variations of interest in perturbation‐based responses of single‐cell transcriptomics data (Materials and Methods). Notably, for the generalizability to three or more conditions, we can apply CAPER in a pairwise manner. The model explicitly separates two intertwined sources of variability, i.e., perturbation‐induced biological effects and unwanted variations, by introducing two sets of latent factors (Figure 1A). Specifically, (1) the shared latent factors capture baseline gene‐expression levels common to both conditions and encode condition‐associated biological effects; whereas (2) the condition‐specific latent factors capture unwanted variations unique to each condition. For each gene *g*, the transformed gene expression profiles from control (*c*) and stimulated (*s*) cells were modeled as follows:

(1) $$\begin{array}{ccc} x_{g}^{c} & = & \mu^{c}+\Lambda^{c}z_{g}^{t}+\Lambda_{u}^{c}z_{g}^{c}+\varepsilon_{g}^{c};\;x_{g}^{s}\\ & = & \mu^{s}+\Lambda^{s}z_{g}^{t}+\Lambda_{u}^{s}z_{g}^{s}+\varepsilon_{g}^{s},g=1,2,\cdots ,G. \end{array}$$

where $x_{g}^{m}\in R^{n^{m}}\left(m=c\;or\;s\right)$ denotes the vector of expression values for gene *g* measured in *n^m^* cells; $\mu^{m}\in R^{n^{m}}\;\left(m=c\;or\;s\right)$ is the condition‐specific mean expression; $\Lambda^{m}\in R^{n^{m}\times k_{1}}\left(m=c\;or\;s\right)$ is the cell‐level global low‐dimensional latent factors. These terms are used to model the condition‐associated biological effects; $\Lambda_{u}^{m}\in R^{n^{m}\times k_{2}}\left(m=c\;or\;s\right)$ denotes the cell‐level condition‐specific low‐dimensional latent factors. These terms are used to model the unwanted variations. $z_{g}^{t}\in R^{k_{1}}$ represents global gene‐level effects across conditions, where $z_{g}^{t}∼\mathrm{MVN}\left(0,\;I_{k_{1}\times k_{1}}\right)$; $z_{g}^{m}\in R^{k_{2}},\left(m=c\;or\;s\right)$ represents condition‐specific factor loading vector, where $z_{g}^{m}∼\mathrm{MVN}\left(0,\;\sigma_{m}I_{k_{2}\times k_{2}}\right)$. The scale parameters  σ_*c*_ and  σ_*s*_ were predefined (fixed values) by the scRNA‐seq data to prevent the identifiability issue. Together, $\Lambda^{m}z_{g}^{t}$ captures shared cell‐state structure and reproducible condition‐associated biological signals, whereas $\Lambda_{u}^{m}z_{g}^{m}$ captures residual condition‐specific unwanted variation.

Our primary goal is to infer the cell‐level global low‐dimensional latent factors $\Lambda^{m}\in R^{n^{m}\times k_{1}}\left(m=c\;or\;s\right)$, which are hypothesized to encode the true biological differences arising from perturbations, disease states, or other experimental conditions. Specifically, we reconstructed the batch‐corrected gene expression matrix using the estimates of $\Lambda^{m}\left(m=c\;or\;s\right)\;\left({\hat{\Lambda }}^{m}\right)$ and the posterior of $z_{g}^{t}$ (${\hat{z}}_{g}^{t}$), estimated by expectation–maximization (EM) algorithm [20], i.e.,

(2) $${\hat{x}}_{g}^{c}={\hat{\mu }}^{c}+{\hat{\Lambda }}^{c}{\hat{z}}_{g}^{t};\;{\hat{x}}_{g}^{s}={\hat{\mu }}^{s}+{\hat{\Lambda }}^{s}{\hat{z}}_{g}^{t},g=1,2,\cdots ,G.$$

The reconstructed gene expression data ${\hat{x}}_{g}^{c}$ and ${\hat{x}}_{g}^{s}$ remain applicable across high‐, confounded‐, and low‐SNR scenarios. By retaining condition‐associated biological signals, preserving separation among cell populations, and removing batch‐associated unwanted variation, the reconstructed matrices support a wide range of downstream analyses, including the identification of condition‐associated cell populations, cell clustering, differential expression analysis, gene co‐expression analysis, and trajectory inference (Figure 1B). We refer to the above method as CAPER (**C**ondition‐**A**ssociated **P**attern **E**xtraction and **R**ecovery). CAPER is implemented as an R package with performance‐critical C/C++ modules linked via Rcpp. CAPER software is publicly available at: https://github.com/liye‐me/CAPER.

### 2.2 Benchmarking CAPER in Simulations

We first benchmarked CAPER against six existing batch correction methods that can generate batch‐corrected gene expression data for downstream analysis, including CellANOVA, scDisInFact, scVI, scGen, ComBat, and fastMNN, together with the uncorrected raw data, using simulated two‐condition scRNA‐seq datasets with controlled signal‐to‐noise ratios (SNRs) (Materials and Methods). The simulations were designed to evaluate whether each method could remove donor‐associated unwanted variation while preserving cell‐type identity and condition‐associated biological signals. The three simulated scenarios corresponded to high‐, confounded‐, and low‐SNR settings, with SNRs of 6.896, 1.903, and 0.826, respectively. In the simulation design, the cell‐type response strength was heterogeneous, with CP1 showing the strongest response, followed by CP2, whereas CP3 and CP4 showed weaker responses, thereby allowing us to assess whether CAPER could recover cell‐type‐specific condition responses rather than producing global condition separation. To visualize cell distributions after batch correction, we performed principal component analysis (PCA) on each method‐specific corrected expression matrix and used the top 30 principal components (PCs) as input for uniform manifold approximation and projection (UMAP) visualization [21], as well as for evaluating biological‐conservation and batch correction performance.

Across all simulated settings, CAPER showed consistently stronger performance than other methods. It achieved the highest overall benchmark score in the high‐, confounded‐, and low‐SNR scenarios, suggesting that its performance was stable across different noise regimes. Importantly, CAPER maintained a favorable balance between biological conservation and batch correction (Materials and Methods).

**FIGURE 2:**
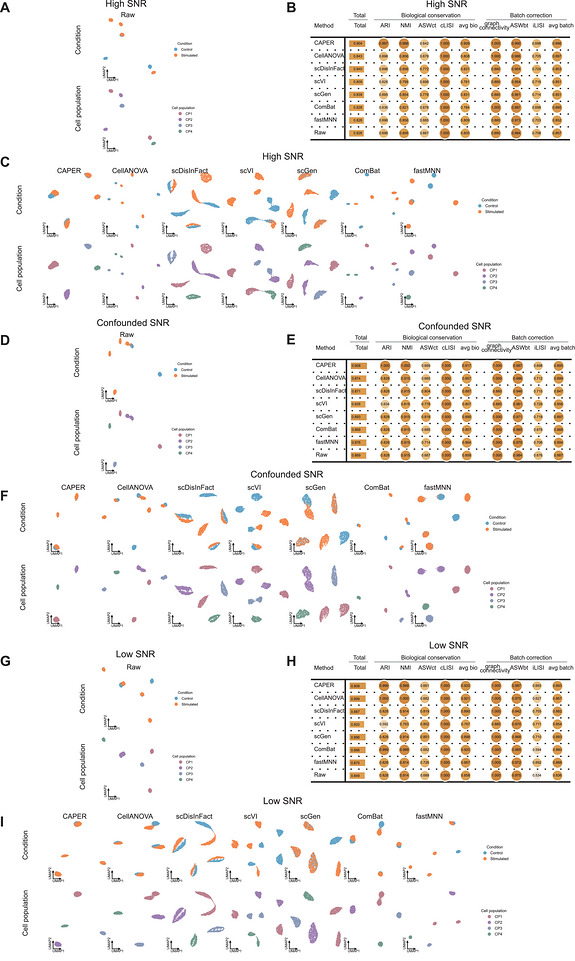
Benchmarking CAPER in simulations across different signal‐to‐noise ratio settings. (A) UMAP visualization of the uncorrected raw simulated data in the high‐SNR scenario. Cells are colored by condition and cell population, showing that the condition‐associated signal is stronger than noise before correction. (B) Quantitative benchmark in the high‐SNR scenario. The bubble plot summarizes the total score, biological conservation metrics, and batch‐correction metrics across methods, showing that CAPER achieves the highest total score while maintaining a favorable balance between biological conservation and batch correction. (C) UMAP visualization of corrected simulated data in the high‐SNR scenario. UMAP plots show cells colored by condition and cell population, demonstrating that CAPER preserves cell‐population structure and condition‐associated biological signal while reducing unwanted variation. (D) UMAP visualization of the uncorrected raw simulated data in the confounded‐SNR scenario. Cells are colored by condition and cell population, showing that the condition‐associated signal is comparable to noise before correction. (E) Quantitative benchmark in the confounded‐SNR scenario. The bubble plot summarizes benchmark metrics across methods, showing that CAPER achieves the highest total score and maintains a favorable balance between biological conservation and batch correction. (F) UMAP visualization of corrected simulated data in the confounded‐SNR scenario. UMAP plots show cells colored by condition and cell population, demonstrating that CAPER maintains cell‐population organization under stronger confounding. (G) UMAP visualization of the uncorrected raw simulated data in the low‐SNR scenario. Cells are colored by condition and cell population, showing that the condition‐associated signal is weaker than noise before correction. (H) Quantitative benchmark in the low‐SNR scenario. The bubble plot summarizes benchmark metrics across methods, showing that CAPER achieves the highest total score and maintains strong biological conservation and batch‐correction performance. (I) UMAP visualization of corrected simulated data in the low‐SNR scenario. UMAP plots show cells colored by condition and cell population, demonstrating that CAPER preserves cell‐population structure even when condition‐associated signals are weak.

Specifically, in the high‐SNR scenario (SNR = 6.896), the uncorrected raw data showed that the condition‐associated signal was stronger than noise before correction (Figure 2A). CAPER achieved the highest total score (0.904) compared with other methods, while maintaining a favorable balance between biological conservation and batch correction; notably, its biological‐conservation score was substantially higher than those of the compared methods [10] (Figure 2B). In the UMAP visualization of corrected data, CAPER preserved clear cell‐population structure while reducing unwanted variation and also showed distinct separation of the most responsive cell population (CP1; Figure 2C). Although several compared methods achieved comparable batch correction scores, they showed weaker biological conservation than CAPER, with less coherent cell‐population organization in the UMAP visualization. For example, CellANOVA showed fragmented cell‐population structure, whereas scDisInFact, scVI, and scGen retained evident condition‐driven separation or distorted cell‐population distribution. ComBat and fastMNN also showed less clear cell‐population organization than CAPER. These results indicate that CAPER achieved a better balance between preserving biological structure and correcting unwanted variation in the high‐SNR setting.

In the confounded‐SNR scenario (SNR = 1.903), the uncorrected raw data showed that the condition‐associated signal was affected by stronger confounding before correction (Figure 2D). Under this setting, CAPER again achieved the highest total benchmark score (0.908) and maintained a favorable balance between biological conservation and batch correction (Figure 2E). In the UMAP visualization of corrected data, CAPER maintained distinct cell‐population organization while avoiding excessive condition‐driven global segregation (Figure 2F). CellANOVA, scDisInFact, and scGen retained part of the biological structure but showed lower total scores than CAPER, indicating a less optimal balance between biological conservation and batch correction.

In the low‐SNR scenario (SNR = 0.826), the uncorrected raw data showed that the condition‐associated signal was weaker than noise before correction (Figure 2G). Even in this lower‐SNR setting, CAPER maintained the highest total benchmark score (0.909), with strong biological conservation and competitive batch‐correction performance (Figure 2H). In the UMAP visualization of corrected data, CAPER preserved cell‐population structure even when condition‐associated signals were weak (Figure 2I). CellANOVA, scGen, and ComBat achieved relatively close total scores, whereas fastMNN, scDisInFact, scVI, and Raw data showed lower overall performance.

We further performed ablation and sensitivity analyses to evaluate the stability of CAPER. The CAPER workflow remained stable when the number of latent factors was varied across *k*_1_ = 10, 30, and 50, with consistently high total scores across all SNR settings. CAPER variants with and without the correction step showed comparable overall performance, although the correction step modestly improved several batch‐correction‐related metrics in some settings. These results suggest that CAPER is not sensitive to a narrow choice of latent dimensionality and can robustly balance biological conservation, batch correction, and condition‐associated signal recovery (Figure S1A–C). The simulated cell populations were designed to follow a graded response hierarchy, with CP1 > CP2 > CP3 > CP4. CAPER recovered this expected cell‐population‐specific response pattern across SNR settings, with the strongest separation in the high‐SNR setting and weaker separation under low SNR (Figure S1D). This result indicates that CAPER's response recovery improves with increasing signal strength.

Together, these simulation results show that CAPER performs favorably across high‐, confounded‐, and low‐SNR settings. Compared with existing methods, CAPER more consistently preserves cell‐type identity, reduces donor‐associated variation, and recovers cell‐type‐specific condition‐associated signals without inducing excessive global condition separation.

### 2.3 CAPER Identifies Cell Populations and Genes Responsive to the Interferon‐*β* Stimulation

**FIGURE 3:**
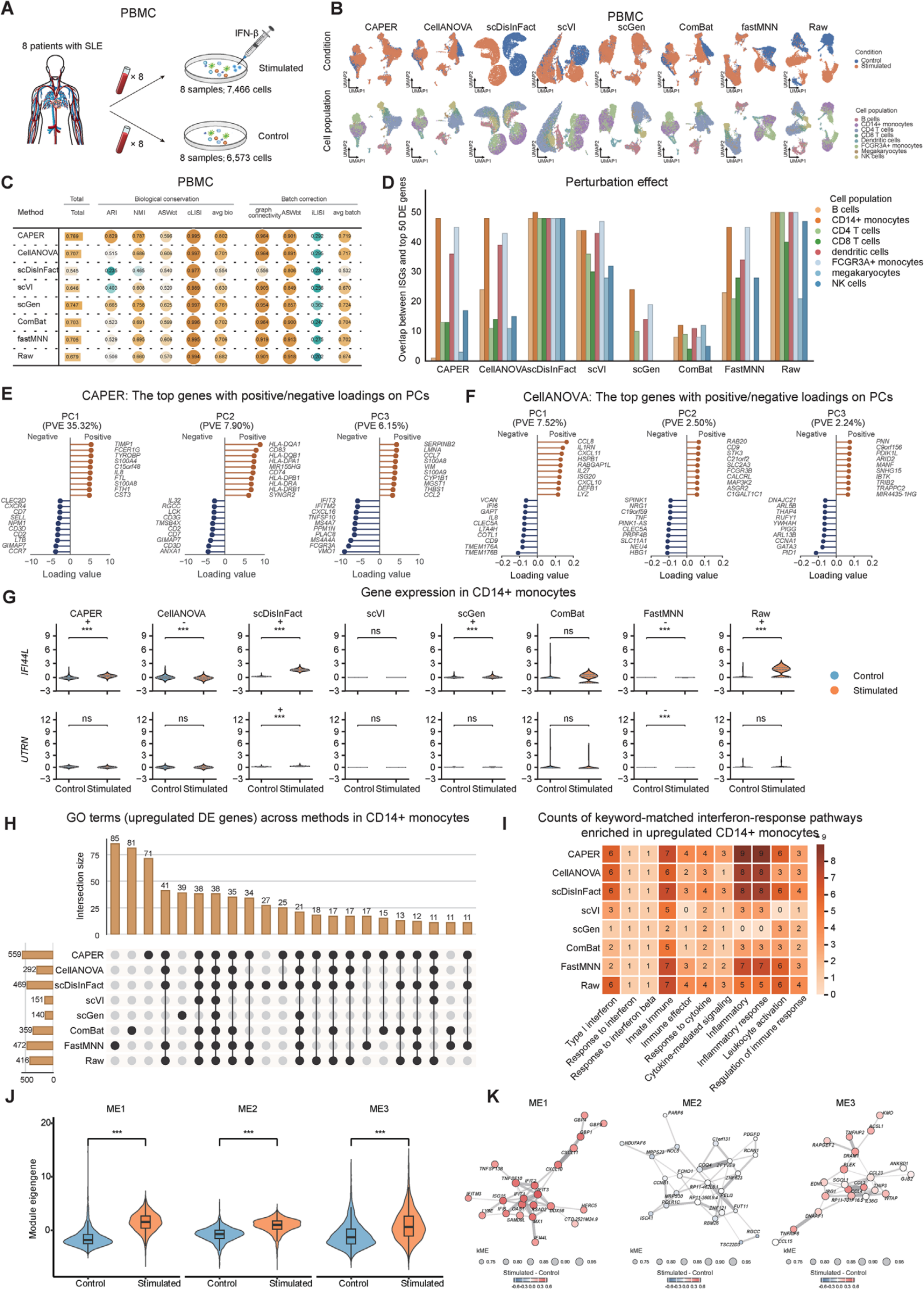
CAPER identifies cell populations and genes responsive to the interferon‐*β* stimulation. (A) Experimental design of the PBMC dataset. Peripheral blood mononuclear cells (PBMCs) were collected from eight patients with systemic lupus erythematosus (SLE). The dataset comprises 7466 cells from the IFN‐*β*‐stimulated condition (stimulated) and 6573 cells from matched control samples, enabling direct comparison of perturbation responses across cell types. (B) UMAP visualization of corrected PBMC data. The gene expression matrices reconstructed by CAPER, CellANOVA, scDisInFact, scVI, scGen, ComBat, fastMNN, and uncorrected Raw were projected into 2D UMAP embeddings. Cells are colored by condition (top row) and annotated cell type (bottom row), demonstrating that CAPER preserves major cell‐population structure while retaining IFN‐*β*‐associated transcriptional differences in responsive populations. (C) Quantitative benchmark of PBMC data integration. The benchmark summary compares overall performance, biological conservation, and batch correction metrics across methods, showing that CAPER achieves the highest total score and the strongest biological conservation. (D) Perturbation response intensity across cell types. Bar plots show the number of overlapping genes between the top 50 upregulated differentially expressed (DE) genes per cell type and a reference set of interferon‐stimulated genes (ISGs) from the Interferome database. CAPER identifies CD14+ monocytes, FCGR3A+ monocytes, and dendritic cells as strongly responsive populations. scDisInFact fails to distinguish perturbation intensity across cell types, yielding uniformly high overlap. (E) Top contributing genes to major axes of variation. Lollipop plots display the top 10 genes with the most positive and most negative loadings on the first three principal components (PCs) identified by CAPER. These genes represent the primary sources of biological variation captured by CAPER. (F) Top contributing genes to major axes of variation. Lollipop plots display the top ten genes with the most positive and most negative loadings on the first three principal components (PCs) identified by CellANOVA. These genes represent the primary sources of biological variation captured by CellANOVA. (G) Corrected expression of representative genes in CD14+ monocytes. Violin plots show expression of *IFI44L* and *UTRN* across stimulated and control cells, demonstrating that CAPER recovers the expected upregulation of the interferon‐stimulated gene *IFI44L* while avoiding a biologically unsupported DE call for *UTRN*. (H) Overlap of GO terms enriched by upregulated DE genes across methods in CD14+ monocyte. The UpSet plot summarizes shared and method‐specific GO terms enriched by upregulated DE genes across CAPER and compared methods. (I) Interferon‐response pathway recovery in CD14+ monocytes. The heatmap summarizes keyword‐matched interferon‐ and immune‐response GO terms enriched among upregulated DE genes, showing stronger pathway‐level recovery by CAPER. (J) Co‐expression module eigengene analysis. Violin plots show stimulated‐control differences in representative co‐expression module eigengenes, indicating that CAPER‐corrected expression supports co‐expression analysis. (K) Hub‐gene networks of co‐expression modules. Network plots show representative hub genes from CAPER‐derived modules, including interferon‐stimulated genes such as *IFIT3*, *CXCL10*, and *ISG15*, and inflammatory chemokine‐related genes such as *CCL23*, *CCL4*, and *CCL3*.

We then applied CAPER to 16 scRNA‐seq datasets from the peripheral blood mononuclear cells (PBMCs) of 8 patients with systemic lupus erythematosus (SLE), which included eight IFN‐*β*‐stimulated samples (stimulated; *N* = 7,466 cells) and eight unstimulated controls (control; *N* = 6,573 cells) [22] (Figure 3A; Materials and Methods). This dataset represented a high‐SNR scenario (SNR = 11.134; Materials and Methods). Because this scRNA‐seq dataset contains explicit treatment conditions with 8 cell populations, the IFN‐*β*‐responsive cell populations and genes are well characterized, and batch effects are expected to be minimal. To provide a comprehensive benchmarking of CAPER's ability to recover biological variation that may be lost during batch integration, we compared CAPER with six existing methods, including CellANOVA [18], scDisInFact [19], scVI [14], scGen [15], ComBat [16], fastMNN [17], and the uncorrected raw data. All methods were applied to the same 3242 highly variable genes (HVGs) after stringent quality controls, followed by the same downstream workflow for visualization, quantitative benchmarking, differential expression analysis, and gene co‐expression analysis (Materials and Methods).

To visualize the cell distribution following batch correction, we performed principal component analysis (PCA) on the corrected expression matrices, and used the top 30 principal components (PCs) as input for UMAP visualization and quantitative benchmarking. As expected, well‐established IFN‐*β*‐responsive cells [22] such as CD14+ monocytes and dendritic cells exhibited the distinct patterns. In contrast, CellANOVA showed a separation of CD4 T cells, which was not consistent with existing knowledge, as T cells (i.e., CD4 and CD8) are generally not strongly responsive to IFN‐*β* [23]. Additionally, scDisInFact insufficiently corrected the gene expression, leading to complete segregation between the stimulated and control groups, and poor separation of major cell populations (Figure 3B). Quantitative benchmarking showed CAPER achieved the highest total score among all methods (total score = 0.769), with the highest average biological conservation score (avg bio = 0.802) and competitive batch‐correction performance (avg batch = 0.719) (Figure 3C). Although scGen showed a slightly higher average batch‐correction score (avg batch = 0.724), its biological conservation score was lower than that of CAPER. CellANOVA, ComBat, fastMNN, scVI, Raw, and especially scDisInFact showed lower total scores, indicating less balanced performance between preserving biological structure and reducing unwanted variation. In particular, the corrected expression matrix generated by scDisInFact retained an overly strong global condition‐associated signal, resulting in pronounced stimulated‐control separation and reduced preservation of cell‐population structure.

In the CAPER model, the cell‐level condition‐specific low‐dimensional latent factors $\Lambda_{u}^{m}$ (*m*  =  *c* *or* *s*; PCs  =  10) were designed to capture variation independent of core cell identity, which we confirmed by the visualization of UMAPs (Figure S2A). To further assess whether CAPER reconstruction preferentially retained stimulation‐relevant biological signals, we compared upregulated DE genes identified from CAPER reconstruction and from the unwanted‐variation component in CD14+ monocytes. Although the two components shared a subset of upregulated DE genes, many genes were specific to either CAPER reconstruction or the unwanted‐variation component (Figure S2B). GO enrichment analysis showed that CAPER reconstruction‐specific upregulated genes were strongly enriched for IFN‐β‐stimulation‐related immune response programs, including defense response (GO:0006952, adjusted *p*‐value = 1.28e‐12) and response to biotic stimulus (GO:0009607, adjusted *p*‐value = 1.41e‐12; Figure S2C). In contrast, unwanted‐variation‐specific genes showed less stimulation‐specific enrichment patterns. These results indicate that CAPER reconstruction preferentially preserved biologically meaningful IFN‐β‐induced signals, whereas the removed component mainly captured residual variation less directly related to the stimulation response.

To assess the biological interpretability of the factorized gene expression patterns, we examined the gene loadings for individual principal components. For CAPER, PC1 explained a large proportion of variance (35.32% variance explained, i.e., PVE = 35.32%) and highlighted inflammation‐ and myeloid‐activation‐related genes, such as *FCER1G*, *IL8*, and *FTL* (Figure 3E). The top 100 genes with negative loadings were associated with immune response and chemotaxis [24, 25] (Figure 3E), i.e., *CCR7*, *GIMAP7*, and *CXCR4*, and were enriched for regulation of immune system process (GO:0002682, adjusted *p*‐value = 1.42e‐5, Figure S2D). For CellANOVA, PC1 (PVE = 7.52%) positive‐loading genes were linked to chemokine signaling (Figure 3F), i.e., *CCL8*, *CXCL11*, and *CXCL10*, while negative‐loading genes were related to membrane processes (Figure 3F), i.e., *TMEM176A/B*, and *CD9*. However, the subsequent CellANOVA components explained less variance and showed weaker functional specificity than the corresponding CAPER components (Figure S2E). We excluded the remaining methods from this comparison because their outputs do not provide directly comparable gene‐loading structures.

We then assessed the reconstruction of gene‐level responses by performing differential expression (DE) analysis between stimulated and control cells (Materials and Methods). Differentially expressed genes were defined using adjusted *p*‐value < 0.05 and |∆mean| > 0.1 (|Δmean| > 0.01 for scGen; |Δmean| > 0.001 for scVI and fastMNN) (Table S3A). A key challenge in analyzing perturbation responses is capturing their cell‐type‐specific difference nature, as interferon‐stimulated genes (ISGs) are well‐known to vary across lineages [23, 26].

To systematically evaluate signal recovery across all cell types, we assessed the enrichment of a curated set of interferon‐stimulated genes from the interferome v2.01 database [27] among the top upregulated DE genes for each cell population (Materials and Methods). CAPER identified CD14+ monocytes, FCGR3A+ monocytes, and dendritic cells as strongly responsive populations, consistent with the known sensitivity of myeloid cells to IFN‐*β* stimulation (Figure 3D and Table S3B) [22]. CellANOVA and several other methods recovered part of this pattern, whereas scVI and ComBat failed to accurately rank the most responsive cell populations. scDisInFact showed high ISG overlap across many cell populations, suggesting reduced specificity in distinguishing cell‐type‐dependent response intensity. These results indicate that CAPER preserves the expected cell‐population‐specific interferon‐response structure.

To quantify perturbation‐based response of each cell type, we also ranked cell‐type‐specific DE genes between two conditions by their statistics from non‐parametric Wilcoxon rank sum test, and then computed pairwise Kendall's τ between cell‐type rankings [28], and summarized global concordance using Kendall's W [29] (Materials and Methods). CAPER showed lower cross‐population concordance (Kendall's W = 0.148), consistent with recovering population‐specific ISG patterns (Figure S2F). This is consistent with known biology: myeloid cells (e.g., CD14+ and FCGR3A+ monocytes) typically mount stronger interferon responses than lymphoid cells (e.g., T and B cells), resulting in higher similarity within myeloid and within lymphoid lineages but lower similarity between them [26]. CellANOVA and scGen also showed low concordance (Kendall's W = 0.107 and 0.275, respectively), whereas scVI showed intermediate concordance (Kendall's W = 0.678). In contrast, scDisInFact and ComBat exhibited high concordance across populations, indicating a failure to resolve this biological heterogeneity (Kendall's W = 0.889 and 0.972, respectively; Figure S2F). This limitation likely arises because scDisInFact's deep‐learning architecture is optimized for multi‐condition integration but lacks explicit modeling for distinct, cell‐type‐specific response patterns [19], whereas ComBat mainly corrects gene‐level additive and multiplicative batch effects and does not directly model heterogeneous condition responses across cell populations [16] (Materials and Methods).

Next, to validate whether the corrected expression matrices successfully recovered biologically meaningful gene‐level signals, we examined representative genes in CD14+ monocytes. CAPER correctly identified the canonical interferon‐stimulated gene *IFI44L* as upregulated in stimulated cells (∆mean = 0.355; adjusted *p*‐value < 1e‐300), consistent with the expected IFN‐*β* response [30, 31, 32] (Figure 3G). scDisInFact (∆mean = 1.456; adjusted *p*‐value < 1e‐300), scGen (∆mean = 0.0539; adjusted *p*‐value = 8.18e‐17), and Raw (∆mean = 1.329; adjusted *p*‐value < 1e‐300) also detected *IFI44L* upregulation. In contrast, although scVI (∆mean = 6.13e‐4; adjusted *p*‐value < 1e‐300) and ComBat (∆mean = −0.0116; adjusted *p*‐value = 5.50e‐73) yielded statistically significant adjusted *p*‐values, their effect sizes did not pass the predefined differential‐expression threshold. CellANOVA (∆mean = −0.157; adjusted *p*‐value = 1.48e‐31) and fastMNN (∆mean = −0.0126; adjusted *p*‐value < 1e‐300) showed negative mean differences, indicating inconsistent recovery of the expected *IFI44L* induction. For *UTRN*, a gene not expected to represent a core IFN‐*β* response in CD14+ monocytes [33] (Figure 3G), CAPER showed only a small negative difference that did not pass the effect‐size threshold (∆mean = −0.088; adjusted *p*‐value = 5.20e‐66). Similarly, CellANOVA (∆mean = −0.0135; adjusted *p*‐value = 0.034), scVI (∆mean = 6.26e‐6; adjusted *p*‐value = 2.44e‐43), scGen (∆mean = −0.00268; adjusted *p*‐value = 8.69e‐3), ComBat (∆mean = 0.0186; adjusted *p*‐value < 1e‐300), and Raw (∆mean = 0.0649; adjusted *p*‐value = 0.103) did not identify *UTRN* as a biologically meaningful DE gene under the predefined criteria. In contrast, scDisInFact identified *UTRN* as upregulated (∆mean = 0.157; adjusted *p*‐value < 1e‐300), while fastMNN showed a negative shift (∆mean = −0.00125; adjusted *p*‐value < 1e‐300). These results indicate that CAPER recovered the expected interferon‐induced expression of *IFI44L* while avoiding spurious effect‐size‐level changes in the non‐core response gene *UTRN*.

Different methods may identify different DE genes that nonetheless participate in related biological processes. To assess functional coherence, we performed gene set enrichment analysis on upregulated DE genes identified by each method in CD14+ monocytes (Materials and Methods). The overlap of enriched GO terms showed that different methods recovered partially shared interferon‐ and immune‐related pathways, while also producing method‐specific functional terms (Figure 3H). We further counted the number of enriched GO terms matching known interferon‐response keywords, including type I interferon, response to interferon, cytokine‐mediated signaling and so on. CAPER showed consistently high keyword‐matched counts across these categories, whereas scVI, scGen, and ComBat showed weaker pathway‐level recovery (Figure 3I and Table S3C).

Finally, we evaluated whether CAPER's corrected expression matrix could be used for stable co‐expression analysis. Three representative co‐expression modules showed significant differences in module eigengene values between control and stimulated CD14+ monocytes (Figure 3J). ME1 and ME3 represented stimulated‐upregulated co‐expression modules, whereas ME2 represented a stimulated‐downregulated module. The hub‐gene networks were biologically interpretable. ME1 contained canonical interferon‐stimulated genes, i.e., IFIT3, CXCL10, ISG15 (Figure 3K), enriched in defense response to virus (GO:0051607, adjusted *p*‐value = 2.36e‐10) and so on (Figure S2G); ME2 contained mitochondrial and ribosomal‐related genes; and ME3 contained inflammatory chemokine‐related genes, i.e., CCL23, CCL4, CCL3 (Figure 3K), enriched in cellular response to interleukin‐1 (GO:0071347, adjusted *p*‐value = 4.40e‐4) and neutrophil chemotaxis (GO:0030593, adjusted *p*‐value = 3.82e‐3) and so on (Figure S2G). These results indicate that CAPER's corrected matrix supports stable co‐expression analysis and recovers biologically coherent gene modules.

Taken together, these results demonstrate that CAPER effectively preserves biological signals in a high‐SNR scenario. The corrected gene expression matrix produced by CAPER therefore provides a highly reliable foundation for identifying the IFN‐*β*‐responsive cell populations and genes and performing gene co‐expression analysis.

### 2.4 CAPER Identifies LUAD‐Associated Cell Populations and Genes

**FIGURE 4:**
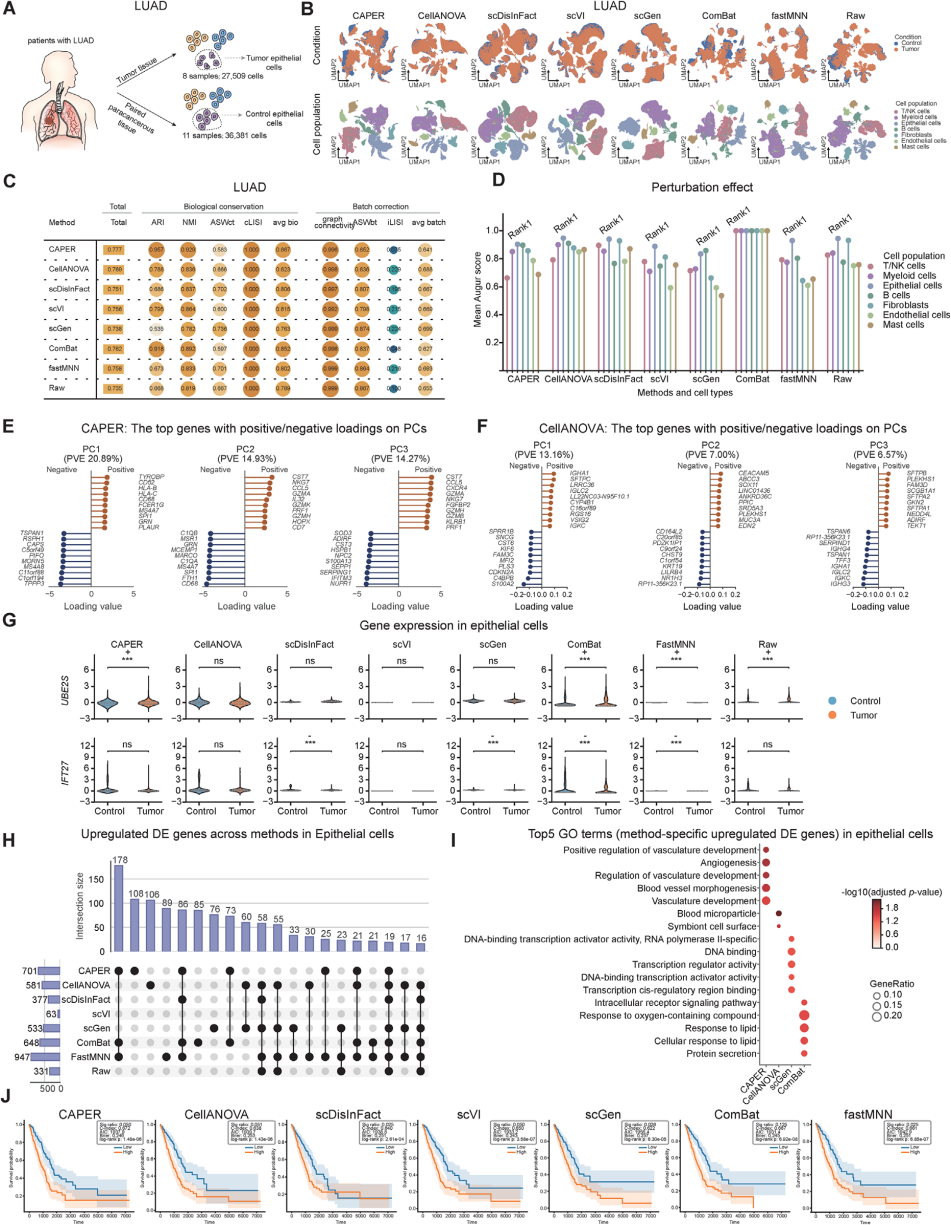
CAPER identifies LUAD‐associated cell populations and genes. (A) Experimental design of the LUAD dataset. The dataset includes 27 509 cells from eight tumor samples and 36 381 cells from eleven paracancerous control samples, enabling analysis of tumor‐associated transcriptional changes in a confounded‐SNR setting. (B) UMAP visualization of corrected LUAD data. The gene expression matrices reconstructed by CAPER, CellANOVA, scDisInFact, scVI, scGen, ComBat, fastMNN, and uncorrected Raw were projected into 2D UMAP embeddings. Cells are colored by condition (tumor vs. control; top row) and annotated cell type (bottom row), demonstrating that CAPER preserves major cell‐population structure while retaining tumor‐control transcriptional differences in LUAD‐relevant populations. (C) Quantitative benchmark of LUAD data integration. The benchmark summary compares overall performance, biological conservation, and batch correction metrics, showing that CAPER achieves the highest total score and strongest biological conservation. (D) Perturbation response intensity across cell populations. Lollipop plots display the mean area under the ROC curve (AUC) from Augur analysis for each cell type, quantifying the responsiveness of each population to tumor‐associated changes. CAPER identifies the strongest responses in epithelial cells, which are known to play critical roles in LUAD pathogenesis. ComBat fails to distinguish response intensity across cell types, yielding uniformly high or flat signals. (E) Top contributing genes to major axes of variation. Lollipop plots display the top ten genes with the most positive and most negative loadings on the first three principal components identified by CAPER. These genes represent the primary sources of tumor‐associated biological variation captured by CAPER. (F) Top contributing genes to major axes of variation. Lollipop plots display the top ten genes with the most positive and most negative loadings on the first three principal components identified by CellANOVA. These genes represent the primary sources of tumor‐associated biological variation captured by CellANOVA. (G) Corrected expression of representative genes in epithelial cells. Violin plots show expression of *UBE2S* and *IFT27* across tumor and control cells, demonstrating that CAPER recovers tumor‐associated upregulation of *UBE2S* while avoiding a statistically unsupported DE call for *IFT27*. (H) Overlap of upregulated DE genes in epithelial cells. The UpSet plot summarizes shared and method‐specific upregulated DE genes across CAPER and compared methods. (I) Functional enrichment of method‐specific upregulated DE genes. Bubble plots show enriched GO terms among method‐specific upregulated DE genes in epithelial cells, indicating that CAPER‐specific genes are enriched for vascular and angiogenesis‐related pathways. (J) Survival prediction using method‐derived gene signatures. Kaplan‐Meier curves show survival probabilities for low‐risk and high‐risk patient groups stratified by Cox proportional hazards models built from the top 40 DE genes identified by each method, applied to independent TCGA LUAD bulk RNA‐seq data. Shaded areas indicate 95% confidence intervals. Insets display key performance metrics for each model: significant gene ratio (proportion of genes with Wald test *p* < 0.05), concordance index (C‐index), Akaike Information Criterion (AIC), Brier score, and log‐rank *p*‐value. The gene signature derived from CAPER's corrected expression matrix significantly stratifies patient survival and achieves competitive predictive performance, including the highest C‐index among the compared methods, suggesting that CAPER‐recovered epithelial signals carry prognostic information.

We next applied CAPER to scRNA‐seq datasets from patients with lung adenocarcinoma (LUAD), which included eight tumor samples (tumor; *N* = 27 509) and eleven paracancerous tissue samples (control; *N* = 36 381) [34] (Figure 4A; Materials and Methods). This dataset represented a confounded‐SNR scenario (SNR = 1.194; Materials and Methods). We aimed to examine whether CAPER could recover the corrected gene expression matrix with cancer signals but heterogeneous tissues. All methods were applied to the same 2640 highly variable genes after stringent quality control, and their corrected or model‐derived expression matrices were used for visualization, benchmarking, differential expression analysis, and downstream analyses (Materials and Methods).

We performed PCA on the corrected expression matrices, retaining the top 30 principal components for UMAP visualization and quantitative benchmarking. The UMAP visualization of the integrated scRNA‐seq data revealed that CAPER preserved major cell‐population structures while retaining tumor‐control transcriptional differences in biologically relevant populations, especially epithelial cells (Figure 4B). In contrast, some competing methods showed less optimal balance between condition mixing and cell‐population preservation, with weaker recovery of LUAD‐relevant tumor‐control differences in epithelial cells. Quantitative benchmarking was then performed using these method‐specific low‐dimensional representations. CAPER achieved the highest total score among all methods (total score = 0.777), with the highest average biological conservation score (avg bio = 0.867) and competitive batch‐correction performance (avg batch = 0.641) (Figure 4C). Although several competing methods, including CellANOVA, scGen, and fastMNN, showed higher average batch‐correction scores, their biological conservation scores were lower than that of CAPER. These results indicate that CAPER achieved a favorable balance between preserving biological structure and reducing unwanted variation in the confounded‐SNR scenario.

Notably, the condition‐specific latent factors $\Lambda_{u}^{m}$ (with top 10 PCs) captured variation independent of core cell identity, which we confirmed by UMAP visualization (Figure S3A). We next compared epithelial‐cell upregulated DE genes identified from CAPER reconstruction and from the unwanted‐variation component. The two components showed limited overlap, with a substantial number of CAPER reconstruction‐specific genes (Figure S3B). GO enrichment analysis showed that CAPER reconstruction‐specific upregulated genes were strongly enriched for epithelial structural programs related to lung epithelial cell states, including cilium (GO:0005929, adjusted *p*‐value = 4.05e‐24) and axoneme (GO:0005930, adjusted *p*‐value = 2.41e‐22; Figure S3C). These epithelial programs were more coherent with LUAD‐associated epithelial remodeling than the enrichment patterns observed in the unwanted‐variation‐specific genes. Together, these results suggest that CAPER reconstruction preferentially retained LUAD‐relevant epithelial signals, while the unwanted‐variation component captured residual signals that were less directly linked to the tumor‐associated epithelial response.

Furthermore, to identify the biologically relevant tumor‐associated cell populations, we used *Augur* [35] to quantify the separability of tumor and control groups (Materials and Methods). As a result, CAPER correctly identified epithelial cells as the most altered cell population in LUAD (mean AUC = 0.904; Figure 4D), consistent with their pathogenic role [36, 37, 38]. Raw reached the same conclusion. In contrast, ComBat showed relatively high and flat Augur scores across multiple cell populations, suggesting reduced specificity in distinguishing LUAD‐associated cell‐type‐dependent responses. scGen also showed different response‐ranking patterns from CAPER, indicating less consistent prioritization of LUAD‐relevant epithelial tumor responses.

To further investigate whether the gene loadings could reveal key LUAD biology, we examined the top 100 genes with the largest positive and negative loadings for the leading principal components. CAPER factors revealed axes contrasting immune activation, myeloid states, cytotoxic lymphocyte programs, and epithelial structural features (Figure 4E). For instance, CAPER's PC1 (PVE = 20.89%) highlighted immune and myeloid‐associated genes such as *TYROBP*, *CD68*, *FCER1G*, and *SPI1* [34, 39, 40], whereas PC2 (PVE = 14.93%) and PC3 (PVE = 14.27%) captured cytotoxic and lymphocyte‐associated genes such as *NKG7*, *CCL5*, *GZMA*, *PRF1*, and *CST7* [41, 42]. Enrichment analysis confirmed that CAPER loading‐associated genes were involved in antigen presentation and immune activation, including antigen processing and presentation of peptide antigen (GO:0048002, adjusted *p*‐value = 2.17e‐11), MHC protein complex (GO:0042611, adjusted *p*‐value = 2.25e‐10), lymphocyte activation (GO:0046649, adjusted *p*‐value = 3.80e‐6), and T cell receptor complex (GO:0042101, adjusted *p*‐value = 2.69e‐6; Figure S3D). These pathways are consistent with antigen presentation, immune‐cell activation, and tumor microenvironment remodeling in LUAD. In contrast, CellANOVA's leading components explained lower variance, with PVE = 13.16% for PC1, PVE = 7.00% for PC2, and PVE = 6.57% for PC3 (Figure 4F). Its loading genes included epithelial, secretory, and immunoglobulin‐related genes such as *S100A2*, *SFTPC*, *IGHA1*, and *IGKC* [34, 39, 43]. GO enrichment of CellANOVA loading‐associated genes was mainly related to IgG immunoglobulin complex (GO:0071735, adjusted *p*‐value = 2.61e‐4), immunoglobulin complex, circulating (GO:0042571, adjusted *p*‐value = 5.16e‐3), epidermis development (GO:0008544, adjusted *p*‐value = 3.79e‐3), cilium organization (GO:0044782, adjusted *p*‐value = 5.04e‐3), and B cell receptor signaling pathway (GO:0050853, adjusted *p*‐value = 5.15e‐4; Figure S3E). These results suggest that CellANOVA also captured tissue‐ and immune‐related signals, but its loading‐associated pathways were less focused on LUAD‐relevant immune activation and tumor microenvironment programs than those recovered by CAPER. We excluded the remaining methods from this comparison because their outputs do not provide directly comparable gene‐loading structures.

We then assessed the reconstruction of gene‐level tumor‐associated responses by performing differential expression (DE) analysis between tumor and control cells (Materials and Methods). Differentially expressed genes were defined using adjusted *p*‐value < 0.05 and |∆mean| > 0.1 (|Δmean| > 0.01 for scGen; |Δmean| > 0.001 for scVI and fastMNN) (Table S4A). In epithelial cells, CAPER identified the LUAD‐associated proliferation‐related gene *UBE2S* as upregulated in tumor cells (∆mean = 0.128; adjusted *p*‐value = 8.07e‐26; Figure 4G) [44, 45]. ComBat (∆mean = 0.120; adjusted *p*‐value = 3.61e‐14), fastMNN (∆mean = 1.02e‐3; adjusted *p*‐value = 5.39e‐10), and Raw (∆mean = 0.108; adjusted *p*‐value = 2.93e‐30) also identified *UBE2S* as upregulated. In contrast, although CellANOVA (∆mean = −0.0670; adjusted *p*‐value = 2.38e‐20), scDisInFact (∆mean = 0.0876; adjusted *p*‐value < 1e‐300), and scVI (∆mean = 7.53e‐5; adjusted *p*‐value = 3.15e‐301) yielded statistically significant adjusted *p*‐values, their effect sizes did not pass the predefined differential‐expression threshold. scGen did not identify *UBE2S* as differentially expressed (∆mean = 0.00957; adjusted *p*‐value = 0.252). For *IFT27*, a gene not expected to represent a core LUAD epithelial tumor‐response signal, CAPER did not identify it as differentially expressed because the adjusted *p*‐value was not significant despite a large mean difference (∆mean = −0.465; adjusted *p*‐value = 0.473) [46]. Similarly, CellANOVA (∆mean = −0.467; adjusted *p*‐value = 0.208) and Raw (∆mean = −0.120; adjusted *p*‐value = 1.00) did not identify *IFT27* as a significant DE gene. In contrast, scDisInFact (∆mean = −0.106; adjusted *p*‐value = 2.88e‐192), scGen (∆mean = −0.0189; adjusted *p*‐value = 4.36e‐25), ComBat (∆mean = −0.419; adjusted *p*‐value = 1.06e‐91), and fastMNN (∆mean = −0.00342; adjusted *p*‐value = 8.28e‐202) identified *IFT27* as negatively changed under the predefined criteria. These results indicate that CAPER recovered the expected tumor‐associated upregulation of *UBE2S* while avoiding a statistically unsupported DE call for the non‐core response gene *IFT27*.

To further evaluate whether each method preserved cell‐type‐specific tumor‐response heterogeneity, we compared differential‐expression gene rankings across cell populations using pairwise Kendall's τ and global Kendall's W. CAPER showed intermediate concordance across cell types, indicating that it captured known biological differences in tumor‐associated responses among distinct cell populations. In contrast, ComBat produced uniformly high concordance across cell types, suggesting that its reconstructed expression matrices tended to smooth or homogenize tumor‐response patterns and thereby reduced cell‐type‐specific biological heterogeneity (Figure S3F).

Different methods may identify different DE genes that nonetheless participate in related biological processes. To assess functional coherence, we performed enrichment analysis separately for method‐specific upregulated and downregulated DE genes in epithelial cells (Materials and Methods). For upregulated DE genes compared with all other methods (Figure 4H), CAPER‐specific genes were enriched for vascular and angiogenesis‐related pathways, including positive regulation of vasculature development (GO:1904018, adjusted *p*‐value = 0.0124), angiogenesis (GO:0001525, adjusted *p*‐value = 0.0127) and blood vessel morphogenesis (GO:0048514, adjusted *p*‐value = 0.0198; Figure 4I). These pathways are consistent with LUAD epithelial tumor programs that interact with the tumor microenvironment through angiogenic and vascular remodeling signals [39, 45, 47]. In contrast, method‐specific upregulated genes identified by the compared methods were enriched for less LUAD epithelial‐relevant or more general biological processes. For example, CellANOVA‐specific genes were enriched for blood microparticle (GO:0072562, adjusted *p*‐value = 0.00601), whereas scGen‐ or ComBat‐specific genes were associated with broader transcriptional or cellular response programs, such as transcription regulator activity (GO:0140110, adjusted *p*‐value = 0.0428) and response to oxygen‐containing compound (GO:1901700, adjusted *p*‐value = 0.0368). Overall, these method‐specific pathways showed less direct relevance to LUAD epithelial tumor programs than the vascular and angiogenesis‐related pathways captured by CAPER. For method‐specific downregulated DE genes (Figure S3G), the significant method‐specific terms were mainly observed in scGen and fastMNN and were dominated by innate immune, leukocyte activation, and antigen receptor‐mediated signaling. This pattern is less consistent with known LUAD epithelial biology (Figure S3H and Table S4B).

Finally, we evaluated whether method‐derived epithelial tumor‐associated genes contained prognostic information for survival analyses in independent TCGA‐LUAD bulk RNA‐seq data. For each method, the top DE genes were used to fit a Cox proportional hazards model, and patients were stratified into high‐ and low‐risk groups (Materials and Methods). CAPER‐derived genes significantly stratified patient survival (log‐rank *p*‐value = 1.48e‐6) and achieved competitive predictive performance (significant gene ratio = 0.050, C‐index = 0.672, AIC = 1937.9, Brier score = 0.246; Figure 4J). Among the significant survival‐associated genes identified by CAPER, *TGFBI* and *GLRX* showed clear biological relevance to LUAD. *TGFBI* is linked to extracellular matrix organization, collagen interaction, and integrin‐related adhesion, suggesting a connection between the CAPER‐derived signature and tumor matrix remodeling [47, 48]. These results suggest that CAPER‑recovered epithelial gene signatures show prognostic associations in independent TCGA data, while larger LUAD cohorts will be needed to establish their broader clinical robustness.

Taken together, these results show that CAPER successfully retains biological signals in LUAD scRNA‐seq experiments, i.e., confounded SNR scenario. The corrected gene expression matrix generated by CAPER, therefore, identifies epithelial tumor‐associated genes with exploratory prognostic associations and provides key cell populations and genes associated with LUAD biology.

### 2.5 CAPER Identifies the T1D‐Dependent Cell Populations

**FIGURE 5:**
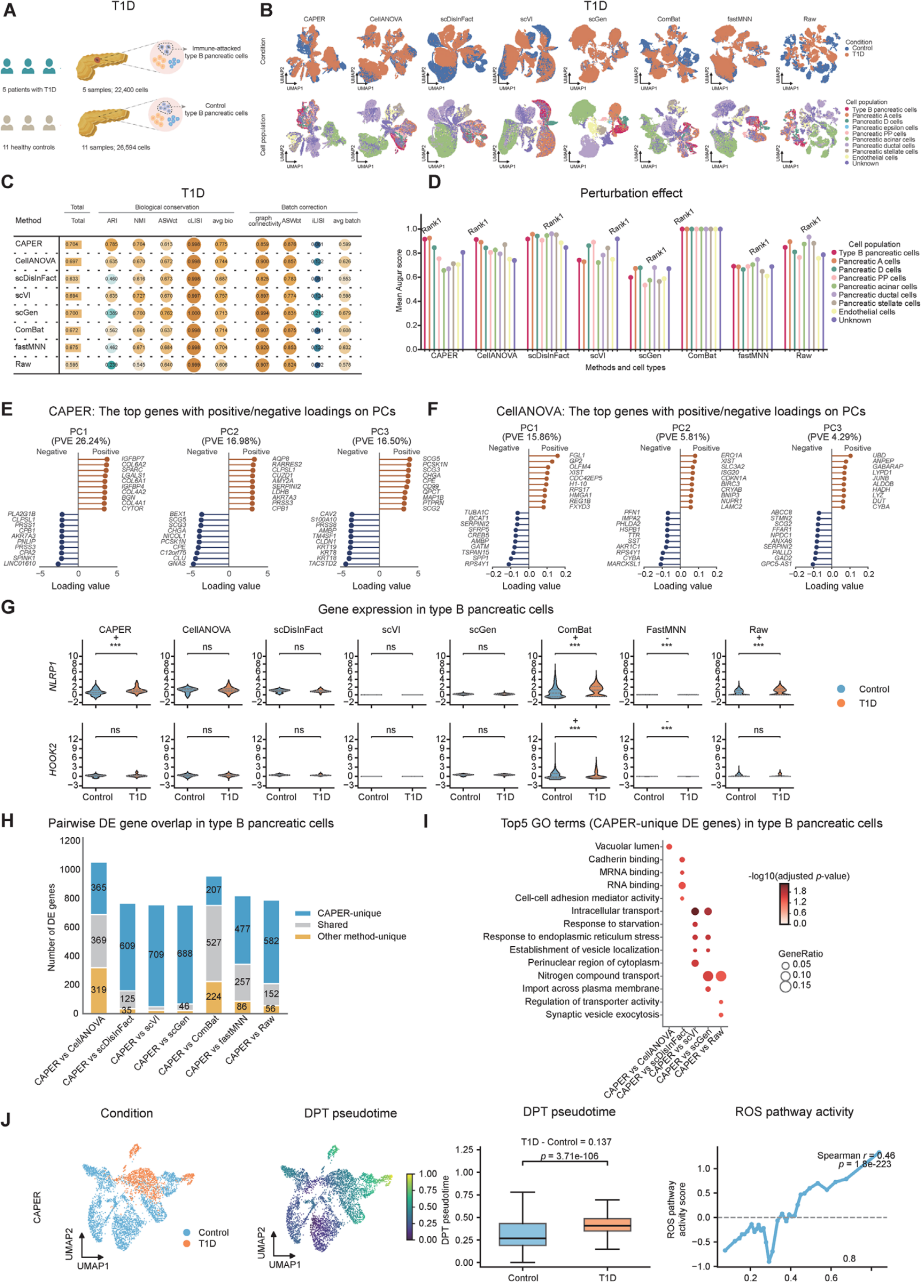
CAPER identifies the T1D‐dependent cell populations and genes. (A) Experimental design of the T1D dataset. Pancreatic islet tissues were collected from five patients with type 1 diabetes (T1D) and eleven healthy controls. The dataset comprises 22 400 cells from T1D donors and 26 594 cells from control donors, enabling investigation of disease‐associated transcriptional changes in rare and subtle cell populations. (B) UMAP visualization of corrected T1D data. The gene expression matrices reconstructed by CAPER, CellANOVA, scDisInFact, scVI, scGen, ComBat, fastMNN, and uncorrected Raw were projected into 2D UMAP embeddings. Cells are colored by condition (T1D vs. control; top row) and annotated cell type (bottom row). CAPER preserves subtle separation between T1D and control cells in disease‐relevant populations while maintaining overall biological structure. (C) Quantitative benchmark of T1D data integration. The benchmark summary compares overall performance, biological conservation, and batch correction metrics, showing that CAPER achieves the highest total score across methods. (D) Perturbation response intensity across cell types. Lollipop plots display the mean area under the ROC curve (AUC) from Augur analysis for each cell type, quantifying the responsiveness of each population to T1D‐associated changes. CAPER and CellANOVA identify the strongest responses in pancreatic A cells and type B pancreatic cells, which are the key cell types implicated in T1D pathogenesis. In contrast, analysis of raw uncorrected data identifies strongest responses in ductal cells, reflecting confounding by sample heterogeneity. scDisInFact produced high Augur scores across multiple endocrine and non‐endocrine populations and ranked pancreatic ductal cells first, suggesting reduced specificity in prioritizing T1D‐relevant endocrine responses. (E) Top contributing genes to major axes of variation. Lollipop plots display the top 10 genes with the most positive and most negative loadings on the first three principal components identified by CAPER. These genes represent the primary sources of T1D‐associated biological variation captured by CAPER. (F) Top contributing genes to major axes of variation. Lollipop plots display the top 10 genes with the most positive and most negative loadings on the first three principal components identified by CellANOVA. These genes represent the primary sources of T1D‐associated biological variation captured by CellANOVA. (G) Corrected expression of representative genes in type B pancreatic cells. Violin plots show expression of *NLRP1* and *HOOK2* across T1D and control cells, demonstrating that CAPER recovers disease‐associated upregulation of *NLRP1* while avoiding a biologically unsupported DE call for *HOOK2*. (H) Pairwise overlap of DE genes in type B pancreatic cells. Stacked bar plots summarize shared, CAPER‐unique, and compared method‐unique DE genes for each CAPER‐vs.‐compared method comparison. (I) Functional enrichment of CAPER‐unique DE genes. Bubble plots show enriched GO terms among CAPER‐unique DE genes from pairwise comparisons, highlighting β‐cell‐relevant vesicle‐localization, endoplasmic‐reticulum‐stress, and synaptic‐vesicle‐exocytosis programs. (J) Trajectory inference and ROS pathway activity analysis using CAPER‐corrected expression. UMAP plots show type B pancreatic cells colored by condition and diffusion pseudotime, followed by pseudotime comparison between T1D and control cells and ROS pathway activity along pseudotime, indicating a T1D‐associated β‐cell progression pattern linked to oxidative‐stress activation.

We applied CAPER to a single‐cell RNA‐seq dataset from pancreatic tissue, comprising samples from five individuals with type 1 diabetes (T1D; N = 22 400 cells) and eleven healthy controls (control; N = 26 594 cells) [49] (Figure 5A; Materials and Methods). This dataset represented a low‐SNR scenario (SNR = 0.611; Materials and Methods). The goal of this application was to examine whether CAPER was sufficiently sensitive to detect disease‐associated changes without being overwhelmed by expression patterns of abundant cell types. All methods were applied to the same 2626 highly variable genes after stringent quality control, and their corrected or model‐derived expression matrices were used for visualization, benchmarking, differential expression analysis, and downstream analyses (Materials and Methods).

We performed PCA on the corrected expression matrices, retaining the top 30 principal components for UMAP visualization and quantitative benchmarking. CAPER preserved the major pancreatic cell‐population structure while retaining T1D‐control transcriptional differences in disease‐relevant endocrine populations, especially type B pancreatic cells and pancreatic A cells (Figure 5B). In contrast, some competing methods showed less balanced patterns between condition‐associated separation and cell‐population preservation. Quantitative benchmarking was then performed using these method‐specific low‐dimensional representations. CAPER achieved the highest total score among all methods (total score = 0.704), with the highest average biological conservation score (avg bio = 0.775) and competitive batch‐correction performance (avg batch = 0.599; Figure 5C). Although scGen, fastMNN, CellANOVA, and ComBat showed higher average batch‐correction scores, their biological conservation scores were lower than that of CAPER. These results indicate that CAPER achieved a favorable overall balance between preserving pancreatic cell identity and reducing unwanted variation in the low‐SNR T1D setting.

Visualization of CAPER's condition‐specific latent space (top 10 PCs) confirmed its role in capturing unwanted variation independently of core cell identity (Figure S4A). We then compared upregulated DE genes in type B pancreatic cells identified from CAPER reconstruction and from the unwanted‐variation component. The two components contained both shared and component‐specific DE genes, with CAPER reconstruction retaining a larger set of specific upregulated genes (Figure S4B). GO enrichment analysis showed that CAPER reconstruction‐specific upregulated genes were enriched for β‐cell functional programs related to secretory activity, including synapse (GO:0045202, adjusted *p*‐value = 1.83e‐6) and regulation of calcium ion‐dependent exocytosis (GO:0017158, adjusted *p*‐value = 7.96e‐4; Figure S4C). These processes are closely related to β‐cell vesicle release and secretory function, which are central to T1D‐associated β‐cell dysfunction. By contrast, unwanted‐variation‐specific genes showed less disease‐targeted enrichment patterns. These results indicate that CAPER reconstruction preferentially preserved biologically relevant T1D‐associated β‐cell functional signals, whereas the unwanted‐variation component mainly represented residual variation outside the reconstructed disease‐associated signal.

Furthermore, the quantification of condition separability using *Augur* [35] confirmed that CAPER correctly identified pancreatic A cells (mean AUC = 0.925) and type B pancreatic cells (mean AUC = 0.917) as the most altered cell populations, consistent with the involvement of endocrine islet cells in T1D pathogenesis [50]. CellANOVA showed a similar pattern, ranking type B pancreatic cells (mean AUC = 0.914) and pancreatic A cells (mean AUC = 0.891) among the most separable populations. In contrast, Raw ranked pancreatic ductal cells as the most separable population (mean AUC = 0.935), suggesting that uncorrected data may retain stronger sample‐composition or tissue‐remodeling signals (Figure 5D). Furthermore, scDisInFact produced high Augur scores across multiple endocrine and non‐endocrine populations and ranked pancreatic ductal cells first (mean AUC = 0.961), indicating reduced specificity in prioritizing T1D‐relevant endocrine responses. These results suggest that CAPER more specifically prioritizes disease‐relevant endocrine populations in the T1D dataset.

CAPER factors revealed interpretable axes related to extracellular matrix remodeling and β‐cell secretory function. For instance, CAPER's PC1 (PVE = 26.24%) highlighted extracellular matrix‐ and stromal‐associated genes such as *IGFBP7*, *COL6A2*, *SPARC*, and *COL4A1*, whereas PC2 (PVE = 16.98%) and PC3 (PVE = 16.50%) captured endocrine secretory genes such as *CHGA*, *SCG3*, *SCG5*, *CPE*, and *PCSK1N* (Figure 5E). Enrichment analysis confirmed that CAPER loading‐associated genes were enriched for extracellular matrix (GO:0031012, adjusted *p*‐value = 3.81e‐13), endoplasmic reticulum lumen (GO:0005788, adjusted *p*‐value = 6.61e‐8), transport vesicle (GO:0030133, adjusted *p*‐value = 3.12e‐11), and secretory vesicle (GO:0099503, adjusted *p*‐value = 5.39e‐5; Figure S4D). These pathways are consistent with β‐cell extracellular remodeling, vesicle trafficking, and endoplasmic reticulum‐associated stress responses in T1D. In contrast, CellANOVA's leading components explained lower variance, with PVE = 15.86% for PC1, PVE = 5.81% for PC2 (Figure 5F). GO enrichment of CellANOVA loading‐associated genes was mainly related to RNA binding (GO:0003723, adjusted *p*‐value = 0.0102), virus receptor activity (GO:0001618, adjusted *p*‐value = 0.00116; Figure S4E). These results suggest that its loading‐associated pathways were less centered on the *β*‐cell vesicle trafficking and endoplasmic reticulum‐related programs recovered by CAPER. We excluded the remaining methods from this comparison because their outputs do not provide directly comparable gene‐loading structures.

We then assessed the reconstruction of gene‐level disease‐associated responses by performing differential expression (DE) analysis between T1D and control cells (Materials and Methods). Differentially expressed genes were defined using adjusted *p*‐value < 0.05 and |∆mean| > 0.1 (|∆mean| > 0.01 for scGen; |∆mean| > 0.001 for scVI and fastMNN) (Table S5A). In type B pancreatic cells, CAPER identified the inflammasome‐related gene *NLRP1* [51, 52] as upregulated in T1D cells (∆mean = 0.520; adjusted *p*‐value = 3.33e‐79; Figure 5G), consistent with enhanced inflammatory stress in disease‐affected β cells. ComBat (∆mean = 0.332; adjusted *p*‐value = 6.33e‐30) and Raw (∆mean = 0.237; adjusted *p*‐value = 3.19e‐12) also detected *NLRP1* upregulation. In contrast, CellANOVA (∆mean = 0.132; adjusted *p*‐value = 0.464) and scGen (∆mean = 0.0163; adjusted *p*‐value = 0.0848) did not identify *NLRP1* as differentially expressed because they failed to meet the statistical‐significance criterion. Although scDisInFact (∆mean = −0.0549; adjusted *p*‐value = 2.85e‐16) and scVI (∆mean = 7.66e‐4; adjusted *p*‐value = 2.79e‐60) yielded significant adjusted *p*‐values, their effect sizes did not pass the predefined thresholds. fastMNN showed a significant negative change (∆mean = −0.00174; adjusted *p*‐value = 1.49e‐7), indicating inconsistent recovery of the expected *NLRP1* induction. For *HOOK2*, a gene not expected to represent a core T1D *β*‐cell disease‐response signal [53, 54], CAPER did not identify it as differentially expressed (∆mean = 0.0849; adjusted *p*‐value = 0.384). Similarly, CellANOVA (∆mean = 0.0267; adjusted *p*‐value = 0.160), scDisInFact (∆mean = −0.0770; adjusted *p*‐value = 6.93e‐54), scVI (∆mean = 2.09e‐4; adjusted *p*‐value = 5.89e‐126), scGen (∆mean = 0.00397; adjusted *p*‐value = 0.414), and Raw (∆mean = −0.0838; adjusted *p*‐value = 5.82e‐13) did not pass the predefined DE criteria because of non‐significant adjusted *p*‐values or insufficient effect sizes. In contrast, ComBat (∆mean = 0.124; adjusted *p*‐value = 1.05e‐14) and fastMNN (∆mean = −0.00116; adjusted *p*‐value = 2.87e‐15) identified significant changes in *HOOK2* with opposite directions. These results indicate that CAPER recovered a disease‐relevant inflammatory signal in *NLRP1* while avoiding spurious effect‐size‐level changes in the non‐core response gene *HOOK2*.

To further assess whether each method preserved cell‐type‐specific T1D‐response heterogeneity, we compared DE gene rankings across cell populations using pairwise Kendall's τ and global Kendall's W. CAPER showed lower concordance between endocrine lineages, including pancreatic A, type B pancreatic, and delta cells, and non‐endocrine lineages, including ductal, stellate, and acinar cells, indicating that it preserved known biological differences between pancreatic cell populations. In contrast, ComBat produced uniformly high concordance across cell types, suggesting that its reconstructed expression matrix tended to smooth T1D‐associated responses and reduce cell‐type‐specific biological heterogeneity. scVI and scDisInFact also showed higher global concordance than CAPER (Figure S4F).

Different methods may identify different DE genes that nonetheless participate in related biological processes. To assess functional coherence, we performed pairwise comparisons between DE genes identified by CAPER and each alternative method in type B pancreatic cells (Materials and Methods). These comparisons separated shared, CAPER‐unique, and other method‐unique genes for each method pair (Figure 5H). CAPER‐unique DE genes more specifically captured β‐cell‐relevant programs related to vesicle trafficking and cellular stress responses, whereas genes uniquely identified by compared methods were mainly associated with broader secretion or mitochondrial processes [49, 55, 56]. For example, CAPER‐unique genes were enriched for intracellular transport (CAPER vs. scVI, GO:0046907, adjusted *p*‐value = 0.0054) and response to endoplasmic reticulum stress (CAPER vs. scGen, GO:0034976, adjusted *p*‐value = 0.0185), two processes closely linked to insulin granule trafficking and β‐cell stress in T1D (Figure 5I). In contrast, compared method‐unique genes were enriched for more general terms such as peptide hormone secretion (scDisInFact vs. CAPER, GO:0030072, adjusted *p*‐value = 0.0298) and mitochondrion (Raw vs. CAPER, GO:0005739, adjusted *p*‐value = 0.0012) (Figure S4G and Table S5B). These results suggest that CAPER more specifically recovers T1D‐associated β‐cell functional programs than the compared methods.

Finally, we evaluated whether CAPER's corrected expression matrix could support trajectory inference and pathway activity analysis in type B pancreatic cells. Using diffusion pseudotime, CAPER revealed a significant positive shift in T1D cells compared with control cells (T1D—control = 0.137, *p* = 3.71e‐106; Figure 5J) [57]. Along this trajectory, reactive oxygen species (ROS) pathway activity increased with pseudotime and showed a positive correlation (Spearman *r* = 0.46, *p* = 1.8e‐223), suggesting that CAPER preserved a disease‐associated progression‐like structure linked to oxidative‐stress activation [49, 58]. Notably, scVI also recovered a positive T1D‐control pseudotime shift and a strong ROS‐pseudotime association (T1D—control = 0.176, *p* = 6.83e‐130; Spearman *r* = 0.77, *p* < 1e‐300; Figure S5C). In contrast, CellANOVA showed a negative ROS association (Spearman *r* = −0.03, *p* = 2.7e‐2; Figure S5A), whereas scGen showed a negative pseudotime shift (T1D—control = −0.072, *p* = 1.83e‐21; Figure S5D). This indicates that CAPER supports trajectory analysis while preserving a biologically interpretable link between T1D‐associated β‐cell progression and ROS activation [56].

Taken together, these results demonstrate that CAPER robustly recovers condition‐associated biological signals even amidst significant clinical heterogeneity, i.e., low SNR scenario. It correctly prioritizes disease‐relevant endocrine populations, recovers biologically plausible expression changes for key genes, and yields functionally coherent results centered on endocrine pathophysiology.

## 3 Discussion

In this study, we developed CAPER, an interpretable matrix factorization framework for multi‐condition single‐cell RNA‐seq data. CAPER models gene expression using two latent components: a shared component that captures cell‐state information and condition‐associated biological variation across conditions, and a condition‐specific component that captures residual unwanted variation, including donor‐, sample‐, and technology‐associated effects. Specifically, the global gene‐level effects $z_{g}^{t}$ define a shared biological coordinate system across conditions, and the corresponding cell‐level global latent factors **Λ** ^*m*^ preserve cell‐state structures and reproducible condition‐associated biological effects. In contrast, the condition‐specific factor loadings $z_{g}^{m}$ and cell‐level condition‐specific latent factors $\Lambda_{u}^{m}$ model residual variation that is specific to each condition. Therefore, CAPER does not simply remove all condition‐specific variation. Instead, biologically meaningful condition‐associated signals are retained in the shared latent space, whereas unwanted residual variation is absorbed by the condition‐specific component. This explicit decomposition makes CAPER interpretable and identifiable under its model structure, and enables the reconstruction of a batch‐corrected expression matrix for downstream analysis.

We first benchmarked CAPER using simulations with controlled SNR and confounding structures. These simulations allowed systematic assessment of whether each method could preserve cell‐type identity, remove unwanted donor‐associated variation, and recover cell‐type‐specific condition responses. Across high‐, confounded‐, and low‐SNR scenarios, CAPER showed stable performance and maintained a favorable balance between biological conservation and batch correction. Ablation and sensitivity analyses further showed that CAPER was robust across a reasonable range of latent dimensions, supporting the stability of its matrix factorization design.

We then validated CAPER in three real‐data applications. In the high‐SNR IFN‐*β* stimulation PBMC dataset, CAPER recovered interpretable inflammatory and immune‐response programs, correctly identified known IFN‐responsive cell populations such as monocytes and dendritic cells, and preserved canonical interferon‐stimulated gene responses. In the confounded‐SNR LUAD dataset, CAPER preserved major cell‐population structures while identifying tumor‐associated epithelial populations, and recovered LUAD‐relevant programs including antigen presentation, immune activation, and tumor microenvironment‐related programs. We emphasize that these survival analyses are exploratory proof‐of‐principle results, indicating that CAPER‐recovered signals are enriched for biologically relevant genes associated with patient outcomes in an independent dataset. In the low‐SNR T1D dataset, CAPER identified disease‐relevant endocrine populations, particularly pancreatic A cells and type B pancreatic cells, and recovered *β*‐cell‐associated inflammatory, secretory, and stress‐response programs. Together, these applications demonstrate that CAPER can recover biologically meaningful condition‐associated signals across controlled perturbation, cancer, and autoimmune disease settings.

Across simulations and real‐data analyses, CAPER showed several practical advantages over existing integration and correction methods probably due to the following three reasons. First, CAPER explicitly models two intertwined perturbation‐induced biological effects and unwanted variations in a statistical framework. Second, CAPER generates a corrected expression matrix, which supports multiple downstream analyses, including clustering, differential expression analysis, gene co‐expression analysis, survival analysis, and trajectory inference. Third, CAPER achieves a “soft” separation of biological effects and unwanted variation through the use of distinct prior variance structures. These features distinguish CAPER from methods that mainly output embeddings, methods that may over‐correct biological signals, and methods that may retain excessive condition‐specific or batch‐associated variation. In our benchmarks, scDisInFact showed weaker preservation of biological signals, especially in the high‐SNR dataset, where the denoised data structure was strongly driven by condition rather than cell‐population identity. This may reflect its conditional variational autoencoder (cVAE) design, which is well‐suited for representation learning and batch integration, but may not be optimal for preserving cell‐population heterogeneity when recovering condition‐associated responses in these datasets.

CAPER also has several practical limitations that should be addressed in future. CAPER may either over‑correct if control samples lack certain batch effects or under‑correct if control samples contain biological variation that is mistakenly treated as batch. In addition, the model does not force all condition‑specific factors to be unwanted; it only removes the component that aligns with the variation observed within control samples. The identifiability of cell‐level embedding factors depends strongly on the scale parameters. Modeling normalized gene expression with a Gaussian approximation could affect the accuracy of batch correction and signal recovery, potentially leading to underestimated uncertainty or distorted loadings for low‑abundance genes. In addition, iterative matrix factorization can become computationally demanding for very large scRNA‐seq datasets. More efficient numerical updates, parallel implementation, and scalable approximation strategies will be important for applying CAPER to large single‐cell atlases and complex disease cohorts.

Several related methods point to useful extensions of CAPER in future. First, CAPER can currently be applied in a pairwise manner to biologically meaningful contrasts. However, we acknowledge that a joint model that directly handles three or more conditions would be a valuable extension. Second, the count nature modeling of scRNA‐seq is a valuable point for future extensions of CAPER. For example, scLM uses a negative‐binomial latent‐variable model to identify consensus co‐expressed gene clusters across multiple single‐cell datasets, highlighting the value of count‐based latent modeling for gene‐module discovery [59]. Third, the integration of diverse molecular data types to predict perturbation responses in a spatially resolved manner is another valuable extension for CAPER. For example, SpaRx uses graph‐based domain adaptation to transfer pharmacogenomic information for predicting spatially heterogeneous drug responses [60].

In summary, CAPER provides an interpretable and flexible framework for recovering condition‐associated biological signals in multi‐condition single‐cell transcriptomics. By separating shared biological structure from condition‐specific unwanted variation, CAPER preserves reproducible biological effects while reducing donor‐, sample‐, and technology‐associated confounding. Its performance across simulation studies and high‐, confounded‐, and low‐SNR real‐data scenarios demonstrates its utility for identifying responsive cell populations, recovering biologically meaningful genes, and supporting diverse downstream analyses. Future extensions to count‐based modeling, multi‐condition designs, spatial and multi‐omics data, and larger disease cohorts will further broaden the applicability and robustness of CAPER.

## 4 Materials and Methods

### 4.1 Parameter Estimates of CAPER

Here, we provided a detailed derivation of parameter estimation for CAPER model using EM algorithm, expanding on the equations presented in the Supporting Information. To facilitate inference, we stack the transformed gene expression profiles from control (*c*) and stimulated (*s*) cells into a single joint Gaussian model, i.e.,

(3) $$x_{g}=\mu_{g}+\Lambda z_{g}+\varepsilon_{g},\;g=1,2,\cdots ,G.$$

where the latent factors $\Lambda =[\begin{array}{ccc} \Lambda^{c} & \Lambda_{u}^{c} & 0\\ \Lambda^{s} & 0 & \Lambda_{u}^{s} \end{array}]$; the loadings $z_{g}=\left[\begin{array}{c} z_{g}^{t}\\ z_{g}^{c}\\ z_{g}^{s} \end{array}\right]$; the data $x_{g}=\left[\begin{array}{c} x_{g}^{c}\\ x_{g}^{s} \end{array}\right]$; the intercept $\mu_{g}=\left[\begin{array}{c} \mu^{c}\\ \mu^{s} \end{array}\right]$; the error term $\varepsilon_{g}=\left[\begin{array}{c} \varepsilon_{g}^{c}\\ \varepsilon_{g}^{s} \end{array}\right]$.

Specifically, $z_{g}^{t}\in R^{k_{1}}$ represents global gene‐level effects across conditions, with a prior distribution that follows a multivariate normal distribution, i.e.,

(4) $$z_{g}^{t}∼\mathrm{MVN}\left(0,\;I_{k_{1}\times k_{1}}\right),\;g=1,2,\cdots ,G.$$

where *G* is the number of genes that commonly shared across all scRNA‐seq data sets; *k*_1_ is the number of latent dimensions. In contrast, the condition‐specific factor loading vector $z_{g}^{m}\in R^{k_{2}}\left(m=c\;or\;s\right)$, with a prior distribution that follows a multivariate normal distribution, i.e.,

(5) $$z_{g}^{m}∼\mathrm{MVN}\left(0,\;\sigma_{m}I_{k_{2}\times k_{2}}\right),\;m=c\;\mathrm{or}\;s;\;g=1,2,\cdots ,G.$$

where  σ_*m*_ is a condition‐specific scaling parameter, set to the mean per‐gene variance in condition *m*; *k*_2_ is the number of latent dimensions. In addition, the residual term $\varepsilon_{g}^{m}\in R^{n^{m}}\;\left(m=c\;or\;s\right)$ was modeled as

(6) $$\varepsilon_{g}^{m}∼\mathrm{MVN}\left(0,\Psi^{m}\right),\;m=c\;\mathrm{or}\;s.$$

where the covariance $\Psi^{m}\in R^{n^{m}\times n^{m}}$ is assumed to be a diagonal matrix; and MVN denotes a multivariate normal distribution.

The loadings ***z**_g_* have a block‐diagonal covariance matrix reflecting their independent scales, the scaling factors  σ_*c*_ and  σ_*s*_ were set as the mean per‐gene variance in the control and stimulated matrices. i.e.,

(7) $$\mathrm{Cov}\;\left(z_{g}\right)=I=\left[\begin{array}{ccc} I_{k_{1}\times k_{1}} & 0 & 0\\ 0 & \;\sigma_{c}I_{k_{2}\times k_{2}} & 0\\ 0 & 0 & \;\sigma_{s}I_{k_{2}\times k_{2}} \end{array}\right].$$

Therefore, the joint distribution of (***z**_g_*, ***x**_g_*) is multivariate Gaussian:

(8) $$\left(\begin{array}{c} z_{g}\\ x_{g} \end{array}\right)∼\mathrm{MVN}\left(0,\left(\begin{array}{cc} I & I\Lambda^{T}\\ \Lambda I & \Lambda I\Lambda^{T}+\Psi \end{array}\right)\right),\;g=1,2,\cdots ,G.$$

Here, we used Cov (***z**_g_*, ***x**_g_*) = * *Cov (***z**_g_*,**Λ** ***z**_g_* + ***ε**_g_*) =** **IΛ** ^*T*^; Var (***x**_g_*) =** **ΛIΛ** ^*T*^ + **Ψ**. The detailed derivations were given in the Supporting Information.

Our primary goal is to infer the cell‐level global low‐dimensional latent factors $\Lambda^{m}\in R^{n^{m}\times k_{1}}\left(m=c\;or\;s\right)$. However, accurate parameter estimation in this MF framework is challenging, as the posterior updates involve repeated matrix inversions within an expectation‐maximization (EM) [20] (Materials and Methods). To enable scalable estimation and robust inference, CAPER leverages a Woodbury formula‐based update strategy to avoid high‐dimensional matrix inversions [61].

In above CAPER model, the parameters ***θ *** =  {**Λ**, **Ψ**} were estimated by maximizing the observed‐data likelihood using the EM algorithm. The complete‐data log‐likelihood (for all genes) is,

(9) $$\begin{array}{ccc} \ell \;\left(\theta \right) & = & \sum_{g=1}^{G}\log\mathrm{Pr}\left(z_{g},x_{g}|\theta \right)\\ & = & \sum_{g=1}^{G}\left[\log\mathrm{Pr}\left(x_{g}|z_{g},\theta \right)+\log\mathrm{Pr}\left(z_{g}\right)\right]. \end{array}$$

where $\mathrm{Pr}(z_{g})$ follows multivariate normal distribution, i.e., $\mathrm{Pr}\left(z_{g}\right)=\mathrm{MVN}\left(0,I\right)$ and $\mathrm{Pr}\left(x_{g}|z_{g},\theta \right)=\mathrm{MVN}\left(\mu_{g}+\Lambda z_{g},\;\Psi \right)$. For simplicity, we assume ***µ**_g_* is zeros because the data were centered by z‐score.

**E‐step**: In the E‐step, by properties of the joint Gaussian distribution, the posterior moments of ***z**_g_* given ***x**_g_* are

(10) $$\begin{array}{ccc} E_{z_{g}|x_{g}}\;\left[z_{g}\right] & = & \mathrm{Cov}\left(z_{g},x_{g}\right)\mathrm{Var}{\left(x_{g}\right)}^{-1}\;x_{g}\\ & = & I\Lambda^{T}{\left(\Lambda I\Lambda^{T}+\Psi \right)}^{-1}\;x_{g}. \end{array}$$

(11) $$\begin{array}{ccc} \mathrm{Va}r_{z_{g}|x_{g}}\;\left(z_{g}\right) & = & \mathrm{Var}\left(z_{g}\right)-\mathrm{Cov}\left(z_{g},x_{g}\right)\mathrm{Var}{\left(x_{g}\right)}^{-1}\mathrm{Cov}\;\left(x_{g},z_{g}\right)\\ & = & I-I\Lambda^{T}{\left(\Lambda I\Lambda^{T}+\Psi \right)}^{-1}\Lambda I. \end{array}$$

(12) $$E_{z_{g}|x_{g}}\;\left[z_{g}{z_{g}}^{T}\right]=\mathrm{Va}r_{z_{g}|x_{g}}\;\left(z_{g}\right)+E_{z_{g}|x_{g}}\left[z_{g}\right]E_{z_{g}|x_{g}}^{T}\left[z_{g}\right].$$

These posterior moments are computed for each gene *g* in the E‐step.

**M‐step**: In the M‐step, we maximize the expected complete‐data log‐likelihood, the *Q*‐function is:

(13) $$\begin{array}{ccc} Q\;\left(\theta |\theta^{\left(i\right)}\right) & = & \int_{z_{g}|x_{g}∼\mathrm{Pr}\left(z_{g}|x_{g},\theta^{\left(i\right)}\right)}\mathrm{Pr}\left(z_{g}|x_{g},\theta^{\left(i\right)}\right)\log\mathrm{Pr}\left(z_{g},x_{g}|\theta \right)dz_{g}\\ & = & \sum_{g=1}^{G}E_{z_{g}|x_{g}}\left[-\frac{N}{2}\log2\pi -\frac{1}{2}\log\left|\Psi \right|\right.\\ & & \left.-\;\frac{1}{2}{\left(x_{g}-\Lambda z_{g}\right)}^{T}\Psi^{-1}\left(x_{g}-\Lambda z_{g}\right)\right]. \end{array}$$

Taking derivatives leads to closed‐form updates. For **Λ**, we have

(14) $$\Lambda_{\mathrm{new}}=\left(\sum_{g=1}^{G}x_{g}E_{z_{g}|x_{g}}^{T}\left[z_{g}\right]\right)\;{\left(\sum_{g=1}^{G}E_{z_{g}|x_{g}}\left[z_{g}{z_{g}}^{T}\right]\right)}^{-1}.$$

For **Ψ**, assuming it is diagonal, we update each diagonal element as,

(15) $$\Psi_{\mathrm{new}}=\frac{1}{G}\;\mathrm{diag}\left(\sum_{g=1}^{G}x_{g}{x_{g}}^{T}-\Lambda_{\mathrm{new}}E_{z_{g}|x_{g}}\left[z_{g}\right]{x_{g}}^{T}\right).$$

The algorithm iterates between E‐step and M‐step until convergence. This detailed derivation clarifies the underlying assumptions and the steps leading to the EM updates are given in the Supporting Information.

### 4.2 Signal‐to‐Noise Ratio Estimation

To quantify the signal‐to‐noise ratio (SNR) of each real dataset, we computed cell‐type‐level SNR using sample‐level pseudobulk expression profiles. Cells were grouped by sample, condition, and cell type, and the mean expression of each gene was calculated within each group to generate pseudobulk profiles.

For each cell population *c*, let ${\bar{x}}_{1cg}$ and ${\bar{x}}_{0cg}$ denote the mean pseudobulk expression of gene *g* in condition 1 and condition 0, respectively, and let $s_{1cg}^{2}$ and $s_{0cg}^{2}$ denote the corresponding across‐sample variances. The SNR for cell population *c* was defined as:

(16) $$\mathrm{SN}R_{c}=\frac{\sum_{g}{\left(\;{\bar{x}}_{1\mathrm{cg}}-{\bar{x}}_{0\mathrm{cg}}\right)}^{2}}{\sum_{g}\left[\frac{1}{2}\left(\;s_{1\mathrm{cg}}^{2}+s_{0\mathrm{cg}}^{2}\right)+\varepsilon \right]}\;.$$

where ε  = 10^−8^ was added for numerical stability. The dataset‐level SNR was defined as the median *SNR_c_* across cell populations. This calculation was performed using the log‐normalized layers.

### 4.3 Simulations

We generated simulated two‐condition scRNA‐seq datasets using *Splatter* [62] package (1.26.0) to evaluate CAPER under three controlled signal‐to‐noise settings. Each dataset contained 5000 cells, 1000 genes, four cell populations, two conditions (Control and Stimulated), and six donors per condition. Cell‐type proportions were imbalanced across the four simulated populations, with proportions of 0.35, 0.30, 0.20, and 0.15 for CP1–CP4, respectively.

Condition‐associated perturbation effects were introduced into stimulated cells in a cell‐population‐specific manner. The response strengths of CP1–CP4 were set to 1.00, 0.65, 0.35, and 0.15, respectively, and stronger‐response cell populations were assigned higher probabilities of perturbation DE genes.

High‐, confounded‐, and low‐SNR scenarios were generated by varying perturbation effect sizes and donor/sample‐associated variation. Specifically, the high‐SNR scenario used a strong perturbation effect and weak donor variation; the confounded‐SNR scenario used an intermediate perturbation effect and moderate donor variation; and the low‐SNR scenario used a weak perturbation effect and strong donor variation (Table S2A,B). The resulting dataset‐level SNR values were 6.896, 1.903, and 0.826 for the high‐, confounded‐, and low‐SNR scenarios, respectively. Method performance was evaluated by biological conservation, batch correction, recovery of condition‐associated structure, and robustness across SNR levels.

To evaluate the stability of CAPER under different implementation settings, we performed ablation and sensitivity analyses using the simulated datasets. For the ablation analysis, we compared CAPER with and without the ComBat‐based correction step to assess whether this component contributed to removing donor‐associated variation. For the sensitivity analysis, we varied the number of latent factors to examine whether CAPER remained stable across latent dimensionalities.

### 4.4 Multi‐Condition scRNA‐seq Data

To evaluate CAPER across distinct signal‐to‐noise scenarios, we applied it to three publicly available scRNA‐seq datasets spanning diverse biological contexts and technical platforms:

1. Interferon‐β‐Stimulated Human PBMCs (High SNR): Peripheral blood mononuclear cells (PBMCs) were collected from eight patients with systemic lupus erythematosus (SLE). For each patient, samples were divided into two conditions: a stimulated group treated with interferon‐*β* (IFN‐*β*) for 6 h and an unstimulated control group. Single‐cell RNA sequencing was performed using the 10x Genomics Chromium platform, yielding a total of 14 039 cells after quality control, with expression measurements for 35 635 genes [22]. This dataset represents a high SNR scenario, as the IFN‐*β* stimulation induces a strong and well‐characterized transcriptional response that dominates over sample heterogeneity.
2. Tumor and Control Lung Cells From LUAD (Confounded SNR): Tissue samples were obtained from patients with lung adenocarcinoma (LUAD), comprising eight tumor biopsies and eleven paracancerous control lung tissue samples. scRNA‐seq libraries were prepared using the 10x Genomics Chromium platform, generating 63 890 cells with 29 634 detected genes [34]. This dataset exemplifies a confounded SNR scenario: the biological differences between tumor and control tissue are substantial, but they are intertwined with patient‐specific heterogeneity and tissue microenvironment effects, making separation challenging.
3. Pancreatic Cells From Type 1 Diabetes Patients and Healthy Controls (Low SNR): Human pancreatic islets were procured through the Human Pancreas Analysis Program (HPAP) with informed donor consent. Donors were diagnosed with type 1 diabetes (T1D) based on clinical criteria and antibody screening. Islet cells were isolated, captured as single cells, and sequenced using the 10x Genomics Chromium platform. For our analysis, we selected cells from 16 donors: five diagnosed with T1D and eleven non‐diabetic controls. The final dataset comprised 48 994 cells and 21 891 genes [49]. This represents a low SNR scenario, as the transcriptional alterations associated with T1D are subtle and localized to specific rare cell types, while sample heterogeneity from donor variability and islet isolation is pronounced (Table S1).

### 4.5 Preprocessing of scRNA‐seq Data

All three datasets were processed using a standardized preprocessing pipeline implemented with Seurat [63]. To preserve condition‐specific biological variation while enabling cross‐condition comparison, we adopted the following strategy. First, for each dataset, the Seurat object was split by experimental condition (e.g., stimulated vs. control, tumor vs. control, T1D vs. control), and each subset was processed independently. Raw counts in each subset were normalized using the *LogNormalize* method, which scales each cell's total counts to 10 000 and applies a natural log transformation. The normalized data were then centered and scaled to unit variance using *ScaleData*, producing z‐scores for each gene across cells. To focus on biologically informative genes, we selected the top 2000 variable features within each subset using the *vst* (variance‐stabilizing transformation) method implemented in *FindVariableFeatures*. The union of highly variable genes selected from the two condition‐specific subsets was used as the common feature space for downstream analysis. This union set served as the input for all methods, ensuring comparability while retaining genes variable in at least one condition.

The specific numbers of highly variable genes retained were: 3242 for the PBMC dataset, 2640 for the LUAD dataset, and 2626 for the T1D pancreatic dataset. For each method, the input matrix was formatted according to its model requirements: scVI and scGen were applied to the raw count matrix of the union of highly variable genes; scDisInFact and fastMNN were applied to the log‐normalized expression matrix; and CAPER, CellANOVA, and ComBat were applied to the scaled z‐score matrix derived from the same common shared genes.

Additionally, for CAPER, we applied ComBat [16] to correct for batch effects across different sequencing batches within the same experimental condition, further reducing unwanted technical variation prior to factor analysis.

### 4.6 Methods for Comparison

We benchmarked CAPER against six existing methods, including CellANOVA, scDisInFact, scVI, scGen, ComBat, and fastMNN. These methods were selected to cover representative strategies for single‐cell data correction and integration, including statistical decomposition, deep generative modeling, empirical Bayes batch correction, and mutual‐nearest‐neighbor‐based correction.

CellANOVA [18] is a lightweight statistical framework designed to recover biological signals that are lost or distorted during standard single‐cell batch integration. Built on a linear decomposition model, it explicitly leverages experimental design by requiring a set of control samples (e.g., healthy individuals or baseline time points) to estimate a latent space of cell‐state‐dependent batch effects, which is then used to remove unwanted variation from all samples while preserving orthogonal biological signals of interest. Applied after an initial integration method such as Harmony or Seurat, CellANOVA outputs a full, interpretable batch‐corrected gene expression matrix that enables downstream analyses like differential expression and pathway enrichment without the distortions introduced by standard integration.

scDisInFact [19] is based on a conditional variational autoencoder (cVAE) that jointly models shared and condition‐specific biological factors. It explicitly disentangles condition‐independent biological states from condition‐specific variations while simultaneously removing batch effects. This deep learning approach enables the identification of condition‐specific key genes and supports predictive tasks, such as forecasting gene expression under unseen conditions or batches. For scDisInFact, reconstructed expression counts were generated using *model.predict_counts()* with *predict_conds = None* to preserve the experimental conditions and *predict_batch = ref_batch* for batch correction, where *ref_batch* was defined as the first donor in the sorted donor list. The resulting batch‐corrected reconstructed expression matrix was used for downstream analyses.

scVI [14] is a variational autoencoder‐based deep generative model for scRNA‐seq data that learns a low‐dimensional latent representation while accounting for batch effects and library‐size variation. We used decoder‐derived normalized expression estimates as the model‐derived expression matrix for downstream analyses.

scGen [15] is a variational autoencoder‐based method for modeling batch or perturbation‐associated shifts in latent space. We applied scGen to log‐normalized expression data and used the batch‐corrected expression matrix returned by scGen for downstream analyses.

ComBat [16] is an empirical Bayes‐based batch correction method that adjusts gene‐wise additive and scale batch effects in continuous expression data. We applied ComBat to the scaled expression matrix and used the corrected matrix for downstream analyses.

fastMNN [17] corrects batch effects by identifying mutual nearest neighbors across batches and estimating correction vectors. We applied fastMNN to log‐normalized expression data and used the reconstructed corrected expression matrix for downstream analyses.

### 4.7 Metrics for Performance Evaluation

To comprehensively assess the effectiveness of integration methods, we evaluated each method from two aspects: biological conservation and batch correction. Biological conservation was quantified using four metrics, including ARI, NMI, ASWct, and cLISI, and the average biological conservation score was calculated as their mean. Batch correction was quantified using three metrics, including graph connectivity, ASWbt, and iLISI, and the average batch correction score was calculated as their mean. All integration metrics were computed using the *scib* python package (version 1.1.7), which provides standardized metrics for benchmarking single‐cell data integration methods [10]. The details of these metrics are provided in Supporting Information. The final total score was computed as:

(17) $$\mathrm{avg}\;\mathrm{bio}\;=\frac{\mathrm{ARI}+\mathrm{NMI}+\mathrm{ASWct}+\mathrm{cLISI}}{4}.$$

(18) $$\mathrm{avg}\;\mathrm{batch}\;=\frac{\mathrm{graph}\;\mathrm{connectivity}+\mathrm{ASWbt}+\mathrm{iLISI}}{3}.$$

(19) Total=0.6×avgbio+0.4×avgbatch.

### 4.8 *Augur* Model for Perturbation Response Quantification

To systematically evaluate cell‐type‐specific sensitivity to simulated condition differences as well as to IFN‐β stimulation or disease conditions in real datasets, and to benchmark the performance of each comparison method in recovering biologically meaningful signals, we employed the *Augur* framework [35].

For each cell type, *Augur* trains a classifier to predict whether a given cell originates from the stimulated/disease group or the control group, using the corrected or model‐derived expression matrices produced by each method. The classifier's performance is assessed through cross‐validation, and the mean area under the receiver operating characteristic curve (AUC) is computed as a quantitative measure of perturbation response intensity for that cell type. A higher AUC indicates that the cell type's transcriptional state is more distinctly altered by the condition, reflecting stronger signal recovery.

We implemented and ran the *Augur* model using the *Pertpy* package (version 1.0.2) [64], applying it uniformly across the expression matrices reconstructed by CAPER, CellANOVA, scDisInFact, scVI, scGen, ComBat, fastMNN, and the raw uncorrected data. This allowed us to compare how well each method preserved the condition‐associated signals that distinguish stimulated/disease cells from control cells across different cell populations.

### 4.9 Interferome Gene List

To quantify the intensity of IFN‐β stimulation effects across different cell types in the PBMC dataset, we derived a reference set of interferon‐stimulated genes (ISGs) from Interferome v2.0 [27], a comprehensive database of interferon‐regulated genes. Interferome integrates publicly available microarray and high‐throughput sequencing experiments to curate genes that are significantly upregulated or downregulated in response to type I, II, or III interferon treatment compared to control conditions. The database serves as a resource for exploring interferon‐activated pathways and their associated biological processes (https://interferome.org/).

For our analysis, we first performed differential expression (DE) analysis between the stimulated and control groups using the reconstructed expression matrices generated by each method, stratified by cell type. From each method‐cell type combination, we extracted the top 50 upregulated genes and intersected them with the Interferome‐derived ISG reference set. The number of overlapping genes was then used as a quantitative measure of each cell type's responsiveness to IFN‐*β* stimulation, enabling cross‐method and cross‐cell‐population comparisons of perturbation signal recovery.

### 4.10 Differential Expression Analysis

To identify genes associated with condition‐specific responses, we performed differential expression (DE) analysis for each cell type by comparing the stimulated/disease group against the control group. All DE analyses were conducted using the Wilcoxon rank‐sum test, implemented via the *rank_genes_groups*() function in the *Scanpy* package (version 1.9.8) [65].

To ensure comparability across methods while accounting for differences in output scales, DE genes were defined using both statistical significance and method‐specific effect‐size thresholds. Genes with adjusted *p*‐value < 0.05 were considered significant, and Δmean was defined as the mean expression difference between the stimulated/disease and control groups. For most methods, DE genes were further required to satisfy |∆mean| > 0.1. Because the output matrices from scVI, scGen, and fastMNN showed compressed mean‐difference scales, smaller cutoffs were used for these methods, with |∆mean| > 0.01 for scGen and |∆mean| > 0.001 for scVI and fastMNN

### 4.11 Kendall's ***τ*** and W for Cell‐Type‐Specific Perturbation‐Response Concordance

For each cell type, we ranked genes by the Wilcoxon rank‐sum statistic comparing the two conditions. We then quantified the similarity of perturbation‐response rankings between cell types using Kendall's rank correlation coefficient (τ). For each pair of cell types *c* and *c*′, Kendall's τ was computed as

(20) $$\tau_{cc^{\prime }}=\frac{N_{\mathrm{con}}-N_{\mathrm{dis}}}{\sqrt{\left(N_{\mathrm{con}}+N_{\mathrm{dis}}+T_{c}\right)\left(N_{\mathrm{con}}+N_{\mathrm{dis}}+T_{c^{\prime }}\right)}}.$$

where *N*_con_ and *N*_dis_ denote the numbers of concordant and discordant gene pairs, respectively, and *T_c_* and *T*_*c*′_ are tie‐correction terms.

To summarize concordance across all *C* cell types, we computed Kendall's coefficient of concordance,

(21) $$\begin{array}{ccc} & & W=\frac{12\sum_{g=1}^{G}{\left(R_{g}-\bar{R}\right)}^{2}}{C^{2}\left(G^{3}-G\right)-C\sum_{c=1}^{C}\sum_{k}\left(t_{\mathrm{ck}}^{3}-t_{\mathrm{ck}}\right)},\\ & & R_{g}=\sum_{c=1}^{C}r_{\mathrm{cg}}\;,\;\bar{R}=\frac{1}{G}\;\sum_{g=1}^{G}R_{g}. \end{array}$$

where *r_cg_* denotes the rank of gene *g* in cell type *c*, and *t_ck_* denotes the size of the *k*‐th tie group in cell type *c*.

### 4.12 Gene Set Enrichment Analysis

To investigate the biological processes underlying method‐specific differences in signal recovery, we performed Gene Ontology (GO) enrichment analysis separately on the uniquely upregulated and uniquely downregulated DE genes identified by each method. Upregulated and downregulated genes were defined according to the direction of the Wilcoxon test scores. Enrichment analysis was conducted in R using the *enrichGO*() function from the *clusterProfiler* package [66], with *org.Hs.eg.db* as the reference annotation database. For each analysis, the background gene set was defined as all genes tested in the corresponding dataset and cell type. Multiple testing correction was applied using the Benjamini‐Hochberg procedure to control the false discovery rate (FDR).

### 4.13 Gene Co‐Expression Analysis

To assess whether CAPER‐recovered expression matrices support gene co‐expression analysis, we performed weighted gene co‐expression network analysis on CD14+ monocytes from the PBMC dataset. We used the R package *hdWGCNA*(version 0.4.11) [67] to construct co‐expression networks from the CAPER‐reconstructed expression matrix. Cells were grouped into control and stimulated conditions according to their condition labels.

We first generated metacells within each condition to reduce the sparsity of single‐cell expression profiles. We then constructed a signed co‐expression network based on Pearson correlation and identified gene modules using the *hdWGCNA* workflow. For each module, we calculated the module eigengene to summarize its overall expression pattern. We ranked modules according to the Wilcoxon test *p*‐values comparing module eigengene values between control and stimulated cells, and selected the top three modules as the most condition‐responsive modules. We further computed module eigengene‐based connectivity (kME) scores to measure the association between each gene and its assigned module. Hub genes were defined as genes with the highest absolute kME within each module.

### 4.14 Survival Prediction Using LUAD Bulk RNA‐seq Data

To evaluate whether the biological signals preserved by CAPER carry stronger prognostic relevance, we performed survival analysis using bulk RNA‐seq data from The Cancer Genome Atlas (TCGA) Lung Adenocarcinoma (LUAD) cohort. This independent dataset allowed us to assess the prognostic relevance of gene signatures derived from each method's corrected expression matrices.

LUAD bulk RNA‐seq data from TCGA were accessed via the Genomic Data Commons (GDC) Data Portal (https://portal.gdc.cancer.gov/). We downloaded level 3 RNA‐seq expression data (HTSeq‐FPKM) along with corresponding clinical annotations, including overall survival time and vital status, for all LUAD patients.

For each method, we first identified the top 40 differentially expressed (DE) genes by ranking genes based on the absolute score from the tumor‐control comparison. These gene sets were then used as candidate prognostic features.

For each method DE gene set, we fitted a multivariable Cox proportional hazards (CoxPH) model using the *lifelines* Python package (version 0.30.0). The model relates patient covariates ***x*** to survival outcomes through the hazard function,

(22) $$h\;\left(t|x\right)=h_{0}\;\left(t\right)\exp\left(x^{T}\beta \;\right).$$

where *h*_0_(*t*) is the baseline hazard and ***β*** is the vector of regression coefficients. The corresponding survival function is

(23) $$S\;\left(t|x\right)=\exp\left\{-H_{0}\left(t\right)\exp\left(x^{T}\beta \right)\right\}.$$

with $H_{0}\;\left(t\right)=\int_{0}^{t}h_{0}\left(u\right)du$ denoting the baseline cumulative hazard. Model coefficients ***β*** were estimated by maximizing the Cox partial likelihood,

(24) $$L_{p}\;\left(\beta \right)=\prod_{i:\delta_{i}=1}\frac{\exp\left(x_{i}^{T}\beta \right)}{\sum_{j\in R\left(T_{i}\right)}\exp\left(x_{j}^{T}\beta \right)}.$$

where *T_i_* is the observed time, δ_*i*_ is the event indicator, and *R*(*T_i_*)  =  {*j*: *T_j_* ≥ *T_i_*} is the risk set at time *T_i_*.

To compare prognostic performance across methods, we computed four complementary metrics: (1) Significant gene ratio, defined as the fraction of model coefficients with Wald test *p* < 0.05 among all genes included in the fitted model; (2) Concordance index (C‐index), obtained from the fitted model to quantify risk‐ranking accuracy; (3) Akaike Information Criterion (AIC) computed from the Cox partial likelihood; and (4) Brier score, computed using the predicted survival probability at the last evaluated time point and the observed event indicator (Supporting Information). All method‐derived gene signatures were analyzed in the same TCGA‐LUAD cohort using an identical preprocessing pipeline and modeling procedure, enabling a consistent comparison of their prognosis‐associated signals.

### 4.15 Trajectory Inference and Pathway Activity Analysis

To evaluate whether corrected expression matrices support trajectory inference analysis, we performed diffusion pseudotime analysis on Type B pancreatic cells from the T1D dataset. For each method, including CAPER, CellANOVA, scDisInFact, scVI, scGen, ComBat, fastMNN, and raw uncorrected data, we used the corresponding reconstructed or corrected expression matrix stored in an AnnData object.

We constructed a nearest‐neighbor graph using PCA representation for each corrected or reconstructed data and the raw uncorrected data. Diffusion maps and diffusion pseudotime were computed using *sc.tl.diffmap()* and *sc.tl.dpt()* [57]. Root cells were selected from control cells with high β‐cell maturity scores and low interferon, HLA, and stress‐response scores. Pseudotime distributions between control and T1D cells were compared using the Wilcoxon rank‐sum test.

Pathway activity along pseudotime was quantified using *sc.tl.score_genes()*. The reactive oxygen species pathway gene set was obtained from the *MSigDB* Hallmark collection, and its association with pseudotime was assessed using Spearman correlation. This analysis evaluated whether each method preserved disease‐associated transcriptional progression and pathway activation in Type B pancreatic cells.

## Author Contributions

S.S. conceived the overarching theme of the study, developed the methodological framework and software implementation, and provided financial support. Y.L. performed the data analysis, refined the method, and drafted the manuscript. JN refined the method. A.W., M.S., Y.C., and G.L. partially performed data analysis and provided analytical suggestions. All authors contributed to writing the manuscript and approved the final version.

## Funding

This study was supported by the STI2030‐Major Projects (Grant No. 2022ZD0208000) to SS; the National Natural Science Foundation of China (Grant Nos. 82574212, 82122061) to SS; the National Key R&D Program of China (Grant No. 2024YFC3405600) to YF and AW; the Fundamental Research Funds for the Central Universities, Xi'an Jiaotong University (Grant Nos. xtr052024011, 11913222000003, and 11913222000007); the Natural Science Basic Research Program of Shaanxi (Grant Nos. 2024JC‐YBMS‐575 and 2023‐JC‐QN‐0737).

## Ethics Declarations

The authors have nothing to report.

## Code Availability Statement

The CAPER software is publicly available at: https://github.com/liye‐me/CAPER.

## Conflicts of Interest

The authors declare no conflicts of interest.

## Supporting information




**Supporting File 1**: advs76186‐sup‐0001‐SuppMat.pdf.

**Supporting File 2**: advs76186‐sup‐0002‐TablesS1‐S5.xlsx.

## Data Availability

The single‐cell RNA‐seq datasets analyzed in this study are publicly available in the Gene Expression Omnibus (GEO) database under the following accession numbers: GSE96583 for interferon‐β‐stimulated human PBMCs; GSE131907 for tumor and control lung cells from lung adenocarcinoma patients, and GSE148073 for pancreatic cells from type 1 diabetes patients and healthy controls.

## References

[1] D. Calderon , R. Blecher‐Gonen , X. Huang , et al., “The Continuum of Drosophila Embryonic Development at Single‐Cell Resolution,” Science 377 (2022): abn5800, 10.1126/science.abn5800. PMC9371440 35926038

[2] Z. Lu , Z. Zhang , Z. Xu , A. Abdulraouf , W. Zhou , and J. Cao , “Organism‐Wide Cellular Dynamics and Epigenomic Remodeling in Mammalian Aging,” Science 391 (2026): adw6273, 10.1126/science.adw6273.41747035

[3] Y. Ma , S. Sun , X. Shang , E. T. Keller , M. Chen , and X. Zhou , “Integrative Differential Expression and Gene Set Enrichment Analysis Using Summary Statistics for scRNA‐seq Studies,” Nature Communications 11 (2020): 1585, 10.1038/s41467-020-15298-6. PMC7101316 32221292

[4] C. Ahlmann‐Eltze and W. Huber , “Analysis of Multi‐Condition Single‐Cell Data With Latent Embedding Multivariate Regression,” Nature Genetics 57 (2025): 659–667, 10.1038/s41588-024-01996-0. 39753773 PMC11906359

[5] H. T. N. Tran , K. S. Ang , M. Chevrier , et al., “A Benchmark of Batch‐Effect Correction Methods for Single‐Cell RNA Sequencing Data,” Genome Biology 21 (2020): 12, 10.1186/s13059-019-1850-9. 31948481 PMC6964114

[6] X. Guo , J. Ning , Y. Chen , et al., “Recent Advances in Differential Expression Analysis for Single‐Cell RNA‐Seq and Spatially Resolved Transcriptomic Studies,” Briefings in Functional Genomics 23 (2024): 95–109, 10.1093/bfgp/elad011. 37022699

[7] Y. Fan , L. Li , and S. Sun , “Powerful and Accurate Detection of Temporal Gene Expression Patterns From Multi‐Sample Multi‐Stage Single‐Cell Transcriptomics Data With TDEseq,” Genome Biology 25 (2024): 96, 10.1186/s13059-024-03237-3. 38622747 PMC11020788

[8] H. C. T. Nguyen , B. Baik , S. Yoon , T. Park , and D. Nam , “Benchmarking Integration of Single‐Cell Differential Expression,” Nature Communications 14 (2023): 1570, 10.1038/s41467-023-37126-3. PMC10030080 36944632

[9] W. Saelens , R. Cannoodt , H. Todorov , and Y. Saeys , “A Comparison of Single‐Cell Trajectory Inference Methods,” Nature Biotechnology 37 (2019): 547–554, 10.1038/s41587-019-0071-9. 30936559

[10] M. D. Luecken , M. Büttner , K. Chaichoompu , et al., “Benchmarking Atlas‐Level Data Integration in Single‐Cell Genomics,” Nature Methods 19 (2022): 41–50, 10.1038/s41592-021-01336-8. 34949812 PMC8748196

[11] I. Korsunsky , N. Millard , J. Fan , et al., “Fast, Sensitive and Accurate Integration of Single‐Cell Data With Harmony,” Nature Methods 16 (2019): 1289–1296, 10.1038/s41592-019-0619-0. 31740819 PMC6884693

[12] K. Polanski , M. D. Young , Z. Miao , K. B. Meyer , S. A. Teichmann , and J.‐E. Park , “BBKNN: Fast Batch Alignment of Single Cell Transcriptomes,” Bioinformatics 36 (2020): 964–965, 10.1093/bioinformatics/btz625. 31400197 PMC9883685

[13] L. Xiong , K. Tian , Y. Li , W. Ning , X. Gao , and Q. C. Zhang , “Online Single‐Cell Data Integration Through Projecting Heterogeneous Datasets Into a Common Cell‐Embedding Space,” Nature Communications 13 (2022): 6118, 10.1038/s41467-022-33758-z. PMC9574176 36253379

[14] R. Lopez , J. Regier , M. B. Cole , M. I. Jordan , and N. Yosef , “Deep Generative Modeling for Single‐Cell Transcriptomics,” Nature Methods 15 (2018): 1053–1058, 10.1038/s41592-018-0229-2. 30504886 PMC6289068

[15] M. Lotfollahi , F. A. Wolf , and F. J. Theis , “scGen Predicts Single‐Cell Perturbation Responses,” Nature Methods 16 (2019): 715–721, 10.1038/s41592-019-0494-8. 31363220

[16] Y. Zhang , G. Parmigiani , and W. E. Johnson , “ComBat‐Seq: Batch Effect Adjustment for RNA‐Seq Count Data,” NAR Genomics and Bioinformatics 2 (2020): lqaa078, 10.1093/nargab/lqaa078. 33015620 PMC7518324

[17] L. Haghverdi , A. T. L. Lun , M. D. Morgan , and J. C. Marioni , “Batch Effects in Single‐Cell RNA‐Sequencing Data are Corrected by Matching Mutual Nearest Neighbors,” Nature Biotechnology 36 (2018): 421–427, 10.1038/nbt.4091. PMC6152897 29608177

[18] Z. Zhang , D. Mathew , T. L. Lim , et al., “Recovery of Biological Signals Lost in Single‐Cell Batch Integration With CellANOVA,” Nature Biotechnology 43 (2025): 1861–1877, 10.1038/s41587-024-02463-1. 39592777

[19] Z. Zhang , X. Zhao , M. Bindra , P. Qiu , and X. Zhang , “scDisInFact: Disentangled Learning for Integration and Prediction of Multi‐Batch Multi‐Condition Single‐Cell RNA‐Sequencing Data,” Nature Communications 15 (2024): 912, 10.1038/s41467-024-45227-w. PMC10827746 38291052

[20] A. P. Dempster , N. M. Laird , and D. B. Rubin , “Maximum Likelihood From Incomplete Data via the EM Algorithm,” Journal of the Royal Statistical Society Series B: Statistical Methodology 39 (1977): 1–22, 10.1111/j.2517-6161.1977.tb01600.x.

[21] L. McInnes , J. Healy , N. Saul , and L. Großberger , “UMAP: Uniform Manifold Approximation and Projection,” Journal of Open Source Software 3 (2018): 861, 10.21105/joss.00861.

[22] H. M. Kang , M. Subramaniam , S. Targ , et al., “Multiplexed Droplet Single‐Cell RNA‐Sequencing Using Natural Genetic Variation,” Nature Biotechnology 36 (2017): 89–94, 10.1038/nbt.4042. PMC5784859 29227470

[23] N. Henig , N. Avidan , I. Mandel , et al., “Interferon‐Beta Induces Distinct Gene Expression Response Patterns in Human Monocytes Versus T Cells,” PLoS One 8 (2013): 62366, 10.1371/journal.pone.0062366. PMC3633862 23626809

[24] R. Förster , A. C. Davalos‐Misslitz , and A. Rot , “CCR7 and Its Ligands: Balancing Immunity and Tolerance,” Nature Reviews Immunology 8 (2008): 362–371, 10.1038/nri2297. 18379575

[25] M. E. Bianchi and R. Mezzapelle , “The Chemokine Receptor CXCR4 in Cell Proliferation and Tissue Regeneration,” Frontiers in Immunology 11 (2020): 2109, 10.3389/fimmu.2020.02109. 32983169 PMC7484992

[26] M. Muckenhuber , I. Seufert , K. Müller‐Ott , et al., “Epigenetic Signals That Direct Cell Type–Specific Interferon Beta Response in Mouse Cells,” Life Science Alliance 6 (2023): 202201823, 10.26508/lsa.202201823. PMC9900254 36732019

[27] I. Rusinova , S. Forster , S. Yu , et al., “INTERFEROME v2.0: An Updated Database of Annotated Interferon‐Regulated Genes,” Nucleic Acids Research 41 (2013): D1040–D1046, 10.1093/nar/gks1215. 23203888 PMC3531205

[28] M. G. Kendall , “A New Measure of Rank Correlation,” Biometrika 30 (1938): 81–93, 10.1093/biomet/30.1-2.81.

[29] M. G. Kendall , “The Treatment of Ties in Ranking Problems,” Biometrika 33 (1945): 239–251, 10.2307/2332303. 21006841

[30] J. W. Schoggins , S. J. Wilson , M. Panis , et al., “A Diverse Range of Gene Products are Effectors of the Type I Interferon Antiviral Response,” Nature 472 (2011): 481–485, 10.1038/nature09907. 21478870 PMC3409588

[31] J. Wang , X. Dang , X. Wu , et al., “DNA Methylation of IFI44L as a Potential Blood Biomarker for Childhood‐Onset Systemic Lupus Erythematosus,” Pediatric Research 96 (2024): 494–501, 10.1038/s41390-024-03135-1. 38514858 PMC11343705

[32] Z. Brkic , N. I. Maria , C. G. van Helden‐Meeuwsen , et al., “Prevalence of Interferon Type I Signature in CD14 Monocytes of Patients With Sjögren's Syndrome and Association With Disease Activity and BAFF Gene Expression,” Annals of the Rheumatic Diseases 72 (2013): 728–735, 10.1136/annrheumdis-2012-201381. 22736090 PMC3618683

[33] L. Falcucci , C. M. Dooley , D. Adamoski , et al., “Transcriptional Adaptation Upregulates Utrophin in Duchenne Muscular Dystrophy,” Nature 639 (2025): 493–502, 10.1038/s41586-024-08539-x. 39939773 PMC11903304

[34] N. Kim , H. K. Kim , K. Lee , et al., “Single‐Cell RNA Sequencing Demonstrates the Molecular and Cellular Reprogramming of Metastatic Lung Adenocarcinoma,” Nature Communications 11 (2020): 2285, 10.1038/s41467-020-16164-1. PMC7210975 32385277

[35] M. A. Skinnider , J. W. Squair , C. Kathe , et al., “Cell Type Prioritization in Single‐Cell Data,” Nature Biotechnology 39 (2021): 30–34, 10.1038/s41587-020-0605-1. PMC7610525 32690972

[36] J. Zhu , Y. Fan , Y. Xiong , et al., “Delineating the Dynamic Evolution From Preneoplasia to Invasive Lung Adenocarcinoma by Integrating Single‐Cell RNA Sequencing and Spatial Transcriptomics,” Experimental & Molecular Medicine 54 (2022): 2060–2076, 10.1038/s12276-022-00896-9. 36434043 PMC9722784

[37] C. J. Hanley , S. Waise , M. J. Ellis , et al., “Single‐Cell Analysis Reveals Prognostic Fibroblast Subpopulations Linked to Molecular and Immunological Subtypes of Lung Cancer,” Nature Communications 14 (2023): 387, 10.1038/s41467-023-35832-6. PMC9889778 36720863

[38] X. Xing , F. Yang , Q. Huang , et al., “Decoding the Multicellular Ecosystem of Lung Adenocarcinoma Manifested as Pulmonary Subsolid Nodules by Single‐Cell RNA Sequencing,” Science Advances 7 (2021): abd9738, 10.1126/sciadv.abd9738. PMC7840134 33571124

[39] M. De Zuani , H. Xue , J. S. Park , et al., “Single‐Cell and Spatial Transcriptomics Analysis of Non‐Small Cell Lung Cancer,” Nature Communications 15 (2024): 4388, 10.1038/s41467-024-48700-8. PMC11116453 38782901

[40] Y. Lavin , S. Kobayashi , A. Leader , et al., “Innate Immune Landscape in Early Lung Adenocarcinoma by Paired Single‐Cell Analyses,” Cell 169 (2017): 750–765.e17, 10.1016/j.cell.2017.04.014. 28475900 PMC5737939

[41] X. Guo , Y. Zhang , L. Zheng , et al., “Global Characterization of T Cells in Non‐Small‐Cell Lung Cancer by Single‐Cell Sequencing,” Nature Medicine 24 (2018): 978–985, 10.1038/s41591-018-0045-3. 29942094

[42] S. S. Ng , F. De Labastida Rivera , J. Yan , et al., “The NK Cell Granule Protein NKG7 Regulates Cytotoxic Granule Exocytosis and Inflammation,” Nature Immunology 21 (2020): 1205–1218, 10.1038/s41590-020-0758-6. 32839608 PMC7965849

[43] E. Fitzsimons , D. Qian , A. Enica , et al., “A Pan‐Cancer Single‐Cell RNA‐Seq Atlas of Intratumoral B Cells,” Cancer Cell 42 (2024): 1784–1797.e4, 10.1016/j.ccell.2024.09.011. 39406187

[44] J.‐Y. Ho , H.‐Y. Lu , H.‐H. Cheng , Y.‐C. Kuo , Y.‐L. A. Lee , and C.‐H. Cheng , “UBE2S Activates NF‐κB Signaling by Binding With IκBα and Promotes Metastasis of Lung Adenocarcinoma Cells,” Cellular Oncology 44 (2021): 1325–1338, 10.1007/s13402-021-00639-4. 34582005 PMC12980711

[45] G. Han , A. Sinjab , Z. Rahal , et al., “An Atlas of Epithelial Cell States and Plasticity in Lung Adenocarcinoma,” Nature 627 (2024): 656–663, 10.1038/s41586-024-07113-9. 38418883 PMC10954546

[46] T. Eguether , J. T. San Agustin , B. T. Keady , et al., “IFT27 Links the BBSome to IFT for Maintenance of the Ciliary Signaling Compartment,” Developmental Cell 31 (2014): 279–290, 10.1016/j.devcel.2014.09.011. 25446516 PMC4254547

[47] Y. Takano , J. Suzuki , K. Nomura , et al., “Spatially Resolved Gene Expression Profiling of Tumor Microenvironment Reveals Key Steps of Lung Adenocarcinoma Development,” Nature Communications 15 (2024): 10637, 10.1038/s41467-024-54671-7. PMC11621540 39639005

[48] A. Chakravarthy , L. Khan , N. P. Bensler , P. Bose , and D. D. De Carvalho , “TGF‐β‐Associated Extracellular Matrix Genes Link Cancer‐Associated Fibroblasts to Immune Evasion and Immunotherapy Failure,” Nature Communications 9 (2018): 4692, 10.1038/s41467-018-06654-8. PMC6224529 30410077

[49] M. Fasolino , G. W. Schwartz , A. R. Patil , et al., “Single‐Cell Multi‐Omics Analysis of Human Pancreatic Islets Reveals Novel Cellular States in Type 1 Diabetes,” Nature Metabolism 4 (2022): 284–299, 10.1038/s42255-022-00531-x. PMC8938904 35228745

[50] R. Melton , S. Jimenez , W. Elison , et al., “Single‐Cell Multiome and Spatial Profiling Reveals Pancreas Cell Type–Specific Gene Regulatory Programs of Type 1 Diabetes Progression,” Science Advances 11 (2025): ady0080, 10.1126/sciadv.ady0080. PMC12422192 40929272

[51] F. R. C. Costa , J. A. Leite , D. M. Rassi , et al., “NLRP1 Acts as a Negative Regulator of Th17 Cell Programming in Mice and Humans With Autoimmune Diabetes,” Cell Reports 35 (2021): 109176, 10.1016/j.celrep.2021.109176. 34038731

[52] S. M. Ghiasi , M. S. Dahllöf , Y. Osmai , et al., “Regulation of the β‐Cell Inflammasome and Contribution to Stress‐Induced Cellular Dysfunction and Apoptosis,” Molecular and Cellular Endocrinology 478 (2018): 106–114, 10.1016/j.mce.2018.08.001. 30121202

[53] C. L. Baron Gaillard , E. Pallesi‐Pocachard , D. Massey‐Harroche , et al., “Hook2 is Involved in the Morphogenesis of the Primary Cilium,” Molecular Biology of the Cell 22 (2011): 4549–4562, 10.1091/mbc.E11-05-0405. 21998199 PMC3226474

[54] E. Pallesi‐Pocachard , E. Bazellieres , A. Viallat‐Lieutaud , et al., “Hook2, a Microtubule‐Binding Protein, Interacts With Par6α and Controls Centrosome Orientation During Polarized Cell Migration,” Scientific Reports 6 (2016): 33259, 10.1038/srep33259. 27624926 PMC5021942

[55] P. Rorsman and F. M. Ashcroft , “Pancreatic β‐Cell Electrical Activity and Insulin Secretion: Of Mice and Men,” Physiological Reviews 98 (2018): 117–214, 10.1152/physrev.00008.2017. 29212789 PMC5866358

[56] M. M. Maestas , M. Ishahak , P. Augsornworawat , et al., “Identification of Unique Cell Type Responses in Pancreatic Islets to Stress,” Nature Communications 15 (2024): 5567, 10.1038/s41467-024-49724-w. PMC11220140 38956087

[57] L. Haghverdi , M. Büttner , F. A. Wolf , F. Buettner , and F. J. Theis , “Diffusion Pseudotime Robustly Reconstructs Lineage Branching,” Nature Methods 13 (2016): 845–848, 10.1038/nmeth.3971. 27571553

[58] B. O. Roep , S. Thomaidou , R. van Tienhoven , and A. Zaldumbide , “Type 1 Diabetes Mellitus as a Disease of the β‐Cell (Do Not Blame the Immune System?),” Nature Reviews Endocrinology 17 (2021): 150–161, 10.1038/s41574-020-00443-4. PMC7722981 33293704

[59] Q. Song , J. Su , L. D. Miller , and W. Zhang , “scLM: Automatic Detection of Consensus Gene Clusters across Multiple Single‐Cell Datasets,” Genomics, Proteomics & Bioinformatics 19 (2021): 330–341, 10.1016/j.gpb.2020.09.002. PMC8602751 33359676

[60] Z. Tang , X. Liu , Z. Li , et al., “SpaRx: Elucidate Single‐Cell Spatial Heterogeneity of Drug Responses for Personalized Treatment,” Briefings in Bioinformatics 24 (2023): bbad338, 10.1093/bib/bbad338. 37798249 PMC10555713

[61] J. Sherman and W. J. Morrison , “Adjustment of an Inverse Matrix Corresponding to a Change in One Element of a Given Matrix,” The Annals of Mathematical Statistics 21 (1950): 124–127, 10.1214/aoms/1177729893.

[62] L. Zappia , B. Phipson , and A. Oshlack , “Splatter: Simulation of Single‐Cell RNA Sequencing Data,” Genome Biology 18 (2017): 174, 10.1186/s13059-017-1305-0. 28899397 PMC5596896

[63] Y. Hao , S. Hao , E. Andersen‐Nissen , et al., “Integrated Analysis of Multimodal Single‐Cell Data,” Cell 184 (2021): 3573–3587.e29, 10.1016/j.cell.2021.04.048. 34062119 PMC8238499

[64] L. Heumos , Y. Ji , L. May , et al., “Pertpy: An End‐to‐End Framework for Perturbation Analysis,” Nature Methods 23 (2026): 350–359, 10.1038/s41592-025-02909-7. 41476114 PMC12904789

[65] F. A. Wolf , P. Angerer , and F. J. Theis , “SCANPY: Large‐Scale Single‐Cell Gene Expression Data Analysis,” Genome Biology 19 (2018): 15, 10.1186/s13059-017-1382-0. 29409532 PMC5802054

[66] G. Yu , L.‐G. Wang , Y. Han , and Q.‐Y. He , “clusterProfiler: An R Package for Comparing Biological Themes Among Gene Clusters,” OMICS: A Journal of Integrative Biology 16 (2012): 284–287, 10.1089/omi.2011.0118. 22455463 PMC3339379

[67] S. Morabito , F. Reese , N. Rahimzadeh , E. Miyoshi , and V. Swarup , “hdWGCNA Identifies Co‐Expression Networks in High‐Dimensional Transcriptomics Data,” Cell Reports Methods 3 (2023): 100498, 10.1016/j.crmeth.2023.100498. 37426759 PMC10326379

