# Supplementary material for: Condition‐Associated Pattern Extraction and Recovery From Multi‐Condition Single‐Cell RNA‐seq Data With CAPER
Source: Adv Sci (Weinh). 2026 Jun 22;13(58):e76186. doi: 10.1002/advs.76186 (PMC13336869; doi:10.1002/advs.76186)
Supplement: Supplementary file 1 [file ADVS-13-e76186-s001.pdf]

Supporting Information

Supporting Files for: Condition-Associated Pattern Extraction and Recovery from Multi-Condition Single-Cell RNA-seq Data with CAPER

Ye Li[1,2], Jin Ning[1,2], An Wang[1,2], Minxi Shi[1,2], Yuanze Chen[1,3], Guoliang Liu[1,2], and Shiquan Sun[1,2,4,5]*

1. Center for Single-Cell Omics and Health, School of Public Health, Xi'an Jiaotong University, Xi'an, Shaanxi 710061, P.R. China
2. Collaborative Innovation Center of Endemic Diseases and Health Promotion in Silk Road Region and NHC Key Laboratory of Environment and Endemic Diseases, Xi'an Jiaotong University, Xi'an, Shaanxi 710061, P.R. China
3. Sun Yat-sen Memorial Hospital, Sun Yat-sen University, Guangzhou, Guangdong 510120, P.R. China
4. Key Laboratory of Environment and Genes Related to Diseases (Xi'an Jiaotong University), Ministry of Education, Xi'an, Shaanxi 710061, P.R. China
5. Key Laboratory for Disease Prevention and Control and Health Promotion of Shaanxi Province, Xi'an, Shaanxi 710061, P.R. China

* Correspondence to: SS (Email: sqsunsph@xjtu.edu.cn)

1. Supporting Text

1.1. Parameter Estimates of CAPER

1.1.1. The Expectation-Maximization (EM) algorithm

The EM algorithm used for parameter estimation is summarized as follows[1]:

Input: Normalized gene expression matrices $\mathbf{X}^c \in \mathbb{R}^{G\times n^c}$ from control group and $\mathbf{X}^s \in \mathbb{R}^{G\times n^s}$ from stimulated group; number of shared latent dimensions k_1; number of condition-specific latent dimensions k_2; maximum number of iterations; convergence threshold ε.

Initialization: Initialize the model parameters $\boldsymbol{\theta}^{(0)} = (\widehat{\boldsymbol{\Lambda}}^{(0)}, \widehat{\boldsymbol{\Psi}}^{(0)})$.

For the iteration $i = 0,1,2,\ldots$ until maximum iteration number or convergence threshold ε:

E-step: compute the posterior moments under the current parameter values $\boldsymbol{\theta}^{(i)}$ for each gene $g = 1,\ldots,G$:

$$\mathrm{E}_{\boldsymbol{z}_g|\boldsymbol{x}_g}[\boldsymbol{z}_g] = \mathbf{I}\boldsymbol{\Lambda}^T(\boldsymbol{\Lambda}\mathbf{I}\boldsymbol{\Lambda}^T + \boldsymbol{\Psi})^{-1}\boldsymbol{x}_g.$$

$$\mathrm{Var}_{\boldsymbol{z}_g|\boldsymbol{x}_g}(\boldsymbol{z}_g) = \mathbf{I} - \mathbf{I}\boldsymbol{\Lambda}^T(\boldsymbol{\Lambda}\mathbf{I}\boldsymbol{\Lambda}^T + \boldsymbol{\Psi})^{-1}\boldsymbol{\Lambda}\mathbf{I}.$$

$$\mathrm{E}_{\boldsymbol{z}_g|\boldsymbol{x}_g}[\boldsymbol{z}_g\boldsymbol{z}_g{}^T] = \mathrm{Var}_{\boldsymbol{z}_g|\boldsymbol{x}_g}(\boldsymbol{z}_g) + \mathrm{E}_{\boldsymbol{z}_g|\boldsymbol{x}_g}[\boldsymbol{z}_g]\mathrm{E}^T_{\boldsymbol{z}_g|\boldsymbol{x}_g}[\boldsymbol{z}_g].$$

M-step: update the model parameters by maximizing the conditional expectation of the complete-data log-likelihood,

$$(\boldsymbol{\theta}|\boldsymbol{\theta}^{(i)}) = \int_{\boldsymbol{z}_g|\boldsymbol{x}_g\sim\Pr(\boldsymbol{z}_g|\boldsymbol{x}_g,\boldsymbol{\theta}^{(i)})} \Pr(\boldsymbol{z}_g|\boldsymbol{x}_g,\boldsymbol{\theta}^{(i)}) \log\Pr(\boldsymbol{z}_g,\boldsymbol{x}_g|\boldsymbol{\theta}) d\boldsymbol{z}_g.$$

Equivalently, update $\boldsymbol{\Lambda}$ and $\boldsymbol{\Psi}$ using the closed-form expressions derived above:

$$\boldsymbol{\theta}^{(i+1)} = (\widehat{\boldsymbol{\Lambda}}^{(i+1)}, \widehat{\boldsymbol{\Psi}}^{(i+1)}).$$

Convergence check: if

$$\left\|\widehat{\boldsymbol{\Lambda}}^{(i)}\widehat{\boldsymbol{\Lambda}}^{(i)T} - \widehat{\boldsymbol{\Lambda}}^{(i+1)}\widehat{\boldsymbol{\Lambda}}^{(i+1)T}\right\|_F^2 < \varepsilon,$$

stop; otherwise, continue to the next iteration.

Output: the final parameter estimates

$$\boldsymbol{\theta} = (\widehat{\boldsymbol{\Lambda}}, \widehat{\boldsymbol{\Psi}}).$$

1.1.2. Parameter initialization

Model parameters were estimated using an expectation–maximization (EM) algorithm. Before running EM, the expression data were scaled and centered within each condition so that $\boldsymbol{\mu}^c = \boldsymbol{\mu}^s = 0$. For initialization, we reformulated the gene-level model in matrix form by stacking $(\boldsymbol{x}_g^m)^T (m = c \text{ or } s)$ across genes, yielding the gene-by-cell expression matrices $\mathbf{X}^c \in \mathbb{R}^{G\times n^c}$ and $\mathbf{X}^s \in \mathbb{R}^{G\times n^s}$. Likewise, the global gene-level loading vectors $\boldsymbol{z}_g^t \in \mathbb{R}^{k_1}$ and

the condition-specific gene-level loading vectors $\boldsymbol{z}_g^m \in \mathbb{R}^{k_2}$ were represented in matrix form by $\mathbf{Z}^t \in \mathbb{R}^{G\times k_1}$ and $\mathbf{Z}^m \in \mathbb{R}^{G\times k_2}$, respectively, whose g-th rows are $(\boldsymbol{z}_g^t)^T$ and $(\boldsymbol{z}_g^m)^T$.

We first initialized the shared gene-level latent matrix from the control data using truncated SVD,

$$\mathbf{X}^c \approx \mathbf{U}^c\mathbf{S}^c(\mathbf{V}^c)^T,\ (\mathbf{Z}^t)^{(0)} = \mathbf{U}^c_{(:,1:k_1)}\left(\mathbf{S}^c_{(1:k1,1:k_1)}\right)^{\frac{1}{2}}.$$

Following the implementation, $(\mathbf{Z}^t)^{(0)}$ was standardized element-wise:

$$(\mathbf{Z}^t)^{(0)} \leftarrow \frac{(\mathbf{Z}^t)^{(0)} - mean(vec((\mathbf{Z}^t)^{(0)}))}{sd(vec((\mathbf{Z}^t)^{(0)}))}.$$

The cell-level global latent factor matrices were then initialized by least squares:

$$(\boldsymbol{\Lambda}^c)^{(0)} = (\mathbf{X}^c)^T(\mathbf{Z}^t)^{(0)}\left(\left((\mathbf{Z}^t)^{(0)}\right)^T(\mathbf{Z}^t)^{(0)}\right)^{-1},\ (\boldsymbol{\Lambda}^s)^{(0)} = (\mathbf{X}^s)^T(\mathbf{Z}^t)^{(0)}\left(\left((\mathbf{Z}^t)^{(0)}\right)^T(\mathbf{Z}^t)^{(0)}\right)^{-1}.$$

Next, we formed residual matrices by removing the fitted global component. For the control condition,

$$\mathbf{R}^c = \mathbf{X}^c - (\mathbf{Z}^t)^{(0)}\left((\boldsymbol{\Lambda}^c)^{(0)}\right)^T.$$

We then applied a rank-k_2 truncated SVD to $\mathbf{R}^c$,

$$\mathbf{R}^c \approx \mathbf{U}^c_u\mathbf{S}^c_u(\mathbf{V}^c_u)^T,\ \ (\mathbf{Z}^c)^{(0)} = \mathbf{U}^c_{u,(:,1:k_2)}\left(\mathbf{S}^c_{u,(1:k_2,1:k_2)}\right)^{1/2},$$

followed by the same element-wise standardization. The control-specific cell-level latent factor matrix was initialized as

$$(\boldsymbol{\Lambda}^c_u)^{(0)} = (\mathbf{R}^c)^T(\mathbf{Z}^c)^{(0)}\left(\left((\mathbf{Z}^c)^{(0)}\right)^T(\mathbf{Z}^c)^{(0)}\right)^{-1}.$$

For the stimulated condition, following the implementation, we first obtained a rank-k_1 low-dimensional representation from $\mathbf{X}^s$,

$$\mathbf{X}^s \approx \mathbf{U}^s\mathbf{S}^s(\mathbf{V}^s)^T,\ \tilde{\mathbf{Z}}^s = \mathbf{U}^s_{(:,1:k_1)}\left(\mathbf{S}^s_{(1:k_1,1:k_1)}\right)^{\frac{1}{2}}.$$

which was standardized in the same way. We then defined

$$\mathbf{R}^s = \mathbf{X}^s - \tilde{\mathbf{Z}}^s\left((\boldsymbol{\Lambda}^s)^{(0)}\right)^T.$$

Applying a rank-k_2 truncated SVD to $\mathbf{R}^s$ gives

$$\mathbf{R}^s \approx \mathbf{U}^s_u\mathbf{S}^s_u(\mathbf{V}^s_u)^T,\ \ (\mathbf{Z}^s)^{(0)} = \mathbf{U}^s_{u,(:,1:k_2)}\left(\mathbf{S}^s_{u,(1:k_2,1:k_2)}\right)^{1/2}.$$

followed by the same element-wise standardization. The stimulated-specific cell-level latent factor matrix was initialized as

$$(\boldsymbol{\Lambda}^s_u)^{(0)} = (\mathbf{R}^s)^T(\mathbf{Z}^s)^{(0)}\left(\left((\mathbf{Z}^s)^{(0)}\right)^T(\mathbf{Z}^s)^{(0)}\right)^{-1}.$$

Finally, the residual covariance matrices were initialized as diagonal identity matrices:

$$(\boldsymbol{\Psi}^c)^{(0)} = \mathbf{I}_{n^c},\ \text{and}\ \ (\boldsymbol{\Psi}^s)^{(0)} = \mathbf{I}_{n^s}.$$

1.1.3. Joint Gaussian model and posterior moments

Under the joint Gaussian model, $\boldsymbol{x}_g = \boldsymbol{\mu}_g + \boldsymbol{\Lambda}\boldsymbol{z}_g + \boldsymbol{\varepsilon}_g,\ g = 1,2,\cdots,G$, where

$$\boldsymbol{z}_g \sim \mathrm{MVN}(\mathbf{0},\mathbf{I}), \boldsymbol{\varepsilon}_g \sim \mathrm{MVN}(\mathbf{0},\boldsymbol{\Psi}),\ g = 1,2,\cdots,G.$$

and $\boldsymbol{z}_g$ and $\boldsymbol{\varepsilon}_g$ are independent. Here, $\mathbf{I}$ denotes the block-diagonal prior covariance matrix of $\boldsymbol{z}_g$,

$$\mathbf{I} = \begin{bmatrix} \mathbf{I}_{k_1\times k_1} & \mathbf{0} & \mathbf{0} \\ \mathbf{0} & \sigma_c \mathbf{I}_{k_2\times k_2} & \mathbf{0} \\ \mathbf{0} & \mathbf{0} & \sigma_s \mathbf{I}_{k_2\times k_2} \end{bmatrix}.$$

rather than a standard identity matrix. After centering within each condition, we have

$$\mathrm{E}[\boldsymbol{z}_g] = 0, \mathrm{E}[\boldsymbol{x}_g] = 0.$$

Therefore, the joint distribution of $\boldsymbol{z}_g$ and $\boldsymbol{x}_g$ is multivariate Gaussian:

$$\begin{pmatrix} \boldsymbol{z}_g \\ \boldsymbol{x}_g \end{pmatrix} \sim \mathrm{MVN}\left(\mathbf{0}, \begin{pmatrix} \mathrm{Var}(\boldsymbol{z}_g) & \mathrm{Cov}(\boldsymbol{z}_g, \boldsymbol{x}_g) \\ \mathrm{Cov}(\boldsymbol{x}_g, \boldsymbol{z}_g) & \mathrm{Var}(\boldsymbol{x}_g) \end{pmatrix}\right), g = 1,2,\cdots,G.$$

where

$$\mathrm{Var}(\boldsymbol{z}_g) = \mathbf{I}.$$

$$\mathrm{Cov}(\boldsymbol{z}_g, \boldsymbol{x}_g) = \mathrm{Cov}(\boldsymbol{z}_g, \boldsymbol{\Lambda}\boldsymbol{z}_g + \boldsymbol{\varepsilon}_g) = \mathbf{I}\boldsymbol{\Lambda}^{\boldsymbol{T}}.$$

$$\mathrm{Cov}(\boldsymbol{x}_g, \boldsymbol{z}_g) = \mathrm{Cov}(\boldsymbol{\Lambda}\boldsymbol{z}_g + \boldsymbol{\varepsilon}_g, \boldsymbol{z}_g) = \boldsymbol{\Lambda}\mathbf{I}.$$

$$\mathrm{Var}(\boldsymbol{x}_g) = \mathrm{Var}(\boldsymbol{\Lambda}\boldsymbol{z}_g + \boldsymbol{\varepsilon}_g) = \boldsymbol{\Lambda}\mathbf{I}\boldsymbol{\Lambda}^{\boldsymbol{T}} + \boldsymbol{\Psi}.$$

Equivalently,

$$\begin{pmatrix} \boldsymbol{z}_g \\ \boldsymbol{x}_g \end{pmatrix} \sim \mathrm{MVN}\left(\mathbf{0}, \begin{pmatrix} \mathbf{I} & \mathbf{I}\boldsymbol{\Lambda}^T \\ \boldsymbol{\Lambda}\mathbf{I} & \boldsymbol{\Lambda}\mathbf{I}\boldsymbol{\Lambda}^T + \boldsymbol{\Psi} \end{pmatrix}\right), g = 1,2,\cdots,G.$$

1.1.4. EM iterative estimation

Based on the complete-data log-likelihood given in main text, CAPER estimates parameters $\boldsymbol{\theta} = \{\boldsymbol{\Lambda}, \boldsymbol{\Psi}\}$ using an EM algorithm consisting of the following E-step and M-step.

E-step: In the E-step, the posterior moments of $\boldsymbol{z}_g$ given $\boldsymbol{x}_g$ are obtained from the conditional distribution of a partitioned multivariate normal vector, i.e.,

$$\mathrm{E}_{\boldsymbol{z}_g|\boldsymbol{x}_g}[\boldsymbol{z}_g] = \mathrm{Cov}(\boldsymbol{z}_g, \boldsymbol{x}_g)\mathrm{Var}(\boldsymbol{x}_g)^{-1}\boldsymbol{x}_g = \mathbf{I}\boldsymbol{\Lambda}^T(\boldsymbol{\Lambda}\mathbf{I}\boldsymbol{\Lambda}^T + \boldsymbol{\Psi})^{-1}\boldsymbol{x}_g.$$

$$\mathrm{Var}_{\boldsymbol{z}_g|\boldsymbol{x}_g}(\boldsymbol{z}_g) = \mathrm{Var}(\boldsymbol{z}_g) - \mathrm{Cov}(\boldsymbol{z}_g, \boldsymbol{x}_g)\mathrm{Var}(\boldsymbol{x}_g)^{-1}\mathrm{Cov}(\boldsymbol{x}_g, \boldsymbol{z}_g) = \mathbf{I} - \mathbf{I}\boldsymbol{\Lambda}^T(\boldsymbol{\Lambda}\mathbf{I}\boldsymbol{\Lambda}^T + \boldsymbol{\Psi})^{-1}\boldsymbol{\Lambda}\mathbf{I}.$$

$$\mathrm{E}_{\boldsymbol{z}_g|\boldsymbol{x}_g}[\boldsymbol{z}_g{\boldsymbol{z}_g}^T] = \mathrm{Var}_{\boldsymbol{z}_g|\boldsymbol{x}_g}(\boldsymbol{z}_g) + \mathrm{E}_{\boldsymbol{z}_g|\boldsymbol{x}_g}[\boldsymbol{z}_g]\mathrm{E}^T_{\boldsymbol{z}_g|\boldsymbol{x}_g}[\boldsymbol{z}_g].$$

To scale inference, CAPER uses Woodbury-based updates to avoid high-dimensional inversions of $\boldsymbol{\Lambda}\mathbf{I}\boldsymbol{\Lambda}^T + \boldsymbol{\Psi}$.[2] Specifically,

$$(\boldsymbol{\Lambda}\mathbf{I}\boldsymbol{\Lambda}^T + \boldsymbol{\Psi})^{-1} = \boldsymbol{\Psi}^{-1} - \boldsymbol{\Psi}^{-1}\boldsymbol{\Lambda}\left(\mathbf{I}_{(k_1+2k_2)\times(k_1+2k_2)} + \mathbf{I}\boldsymbol{\Lambda}^T\boldsymbol{\Psi}^{-1}\boldsymbol{\Lambda}\right)^{-1}\mathbf{I}\boldsymbol{\Lambda}^T\boldsymbol{\Psi}^{-1},$$

where $\mathbf{I}_{(k_1+2k_2)\times(k_1+2k_2)}$ denotes the identity matrix, $\mathbf{I}$ denotes the block-diagonal prior covariance matrix of $\boldsymbol{z}_g$.

M-step: In the M-step, we maximize the conditional expectation of the complete-data log-likelihood with respect to the model parameters. Specifically, the Q-function is defined as

$$Q(\boldsymbol{\theta}|\boldsymbol{\theta}^{(i)}) = \int_{\boldsymbol{z}_g|\boldsymbol{x}_g\sim\Pr(\boldsymbol{z}_g|\boldsymbol{x}_g,\boldsymbol{\theta}^{(i)})} \Pr(\boldsymbol{z}_g|\boldsymbol{x}_g,\boldsymbol{\theta}^{(i)}) \log\Pr(\boldsymbol{z}_g,\boldsymbol{x}_g|\boldsymbol{\theta}) d\boldsymbol{z}_g$$

or equivalently, after summing over all groups $g = 1,2,\cdots,G$,

$$Q(\boldsymbol{\theta}|\boldsymbol{\theta}^{(i)}) = \sum_{g=1}^{G} \mathrm{E}_{\boldsymbol{z}_g|\boldsymbol{x}_g,\boldsymbol{\theta}^{(i)}}\left[\log\Pr(\boldsymbol{z}_g,\boldsymbol{x}_g|\boldsymbol{\theta})\right].$$

Using the decomposition of the complete-data log-likelihood,

$$\log\Pr(\boldsymbol{z}_g,\boldsymbol{x}_g|\boldsymbol{\theta}) = \log\Pr(\boldsymbol{x}_g|\boldsymbol{z}_g,\boldsymbol{\theta}) + \log\Pr(\boldsymbol{z}_g).$$

we obtain

$$Q(\boldsymbol{\theta}|\boldsymbol{\theta}^{(i)}) = \sum_{g=1}^{G} \mathrm{E}_{\boldsymbol{z}_g|\boldsymbol{x}_g,\boldsymbol{\theta}^{(i)}}\left[\log\Pr(\boldsymbol{x}_g|\boldsymbol{z}_g,\boldsymbol{\theta}) + \log\Pr(\boldsymbol{z}_g)\right].$$

Since $\Pr(\boldsymbol{z}_g) = \mathrm{MVN}(\mathbf{0},\mathbf{I})$ does not depend on $\boldsymbol{\Lambda}$ or $\boldsymbol{\Psi}$, maximizing $Q(\boldsymbol{\theta}|\boldsymbol{\theta}^{(i)})$ with respect to $\boldsymbol{\theta} = \{\boldsymbol{\Lambda},\boldsymbol{\Psi}\}$ is equivalent to maximizing

$$Q(\boldsymbol{\theta}|\boldsymbol{\theta}^{(i)}) = \sum_{g=1}^{G} \mathrm{E}_{\boldsymbol{z}_g|\boldsymbol{x}_g,\boldsymbol{\theta}^{(i)}}\left[\log\Pr(\boldsymbol{x}_g|\boldsymbol{z}_g,\boldsymbol{\theta})\right].$$

Because

$$\boldsymbol{x}_g|\boldsymbol{z}_g,\boldsymbol{\theta} \sim \mathrm{MVN}(\boldsymbol{\mu}_g + \boldsymbol{\Lambda}\boldsymbol{z}_g, \boldsymbol{\Psi}).$$

and $\boldsymbol{\mu}_g = 0$ after centering, the conditional log-likelihood is

$$\log\Pr(\boldsymbol{x}_g|\boldsymbol{z}_g,\boldsymbol{\theta}) = -\frac{N}{2}\log 2\pi - \frac{1}{2}\log|\boldsymbol{\Psi}| - \frac{1}{2}(\boldsymbol{x}_g - \boldsymbol{\Lambda}\boldsymbol{z}_g)^T\boldsymbol{\Psi}^{-1}(\boldsymbol{x}_g - \boldsymbol{\Lambda}\boldsymbol{z}_g).$$

where $N = \sum_m n^m$, and n^m is the number of cells for data m. Therefore,

$$Q(\boldsymbol{\theta}|\boldsymbol{\theta}^{(i)}) = \sum_{g=1}^{G} \mathrm{E}_{\boldsymbol{z}_g|\boldsymbol{x}_g}\left[-\frac{N}{2}\log 2\pi - \frac{1}{2}\log|\boldsymbol{\Psi}| - \frac{1}{2}(\boldsymbol{x}_g - \boldsymbol{\Lambda}\boldsymbol{z}_g)^T\boldsymbol{\Psi}^{-1}(\boldsymbol{x}_g - \boldsymbol{\Lambda}\boldsymbol{z}_g)\right].$$

The constant term $-\frac{N}{2}\log 2\pi$ does not affect the maximization over $\boldsymbol{\theta}$ and can be omitted. Expanding the quadratic form gives

$$(\boldsymbol{x}_g - \boldsymbol{\Lambda}\boldsymbol{z}_g)^T\boldsymbol{\Psi}^{-1}(\boldsymbol{x}_g - \boldsymbol{\Lambda}\boldsymbol{z}_g) = \boldsymbol{x}_g^T\boldsymbol{\Psi}^{-1}\boldsymbol{x}_g - 2\boldsymbol{x}_g^T\boldsymbol{\Psi}^{-1}\boldsymbol{\Lambda}\boldsymbol{z}_g + \boldsymbol{z}_g^T\boldsymbol{\Lambda}^T\boldsymbol{\Psi}^{-1}\boldsymbol{\Lambda}\boldsymbol{z}_g.$$

Therefore,

$$Q(\boldsymbol{\theta}|\boldsymbol{\theta}^{(i)}) = \sum_{g=1}^{G}\left[-\frac{1}{2}\log|\boldsymbol{\Psi}| - \frac{1}{2}\boldsymbol{x}_g^T\boldsymbol{\Psi}^{-1}\boldsymbol{x}_g + \boldsymbol{x}_g^T\boldsymbol{\Psi}^{-1}\boldsymbol{\Lambda}\mathrm{E}_{\boldsymbol{z}_g|\boldsymbol{x}_g}[\boldsymbol{z}_g] - \frac{1}{2}\mathrm{E}_{\boldsymbol{z}_g|\boldsymbol{x}_g}\left[\boldsymbol{z}_g^T\boldsymbol{\Lambda}^T\boldsymbol{\Psi}^{-1}\boldsymbol{\Lambda}\boldsymbol{z}_g\right]\right].$$

Using the trace identities

$$\boldsymbol{a}^T\mathbf{B}\boldsymbol{c} = \mathbf{Tr}(\boldsymbol{c}^T\mathbf{B}^T\boldsymbol{a}) \text{ and } \mathbf{E}[\boldsymbol{z}^T\mathbf{A}\boldsymbol{z}] = \mathrm{Tr}(\mathbf{A}\mathbf{E}[\boldsymbol{z}\boldsymbol{z}^T]).$$

we can rewrite the last two terms as

$$\boldsymbol{x}_g^T \boldsymbol{\Psi}^{-1} \boldsymbol{\Lambda} \mathrm{E}_{\boldsymbol{z}_g|\boldsymbol{x}_g}[\boldsymbol{z}_g] = \mathrm{Tr}\left(\mathrm{E}_{\boldsymbol{z}_g|\boldsymbol{x}_g}^T[\boldsymbol{z}_g] \boldsymbol{\Lambda}^T \boldsymbol{\Psi}^{-1} \boldsymbol{x}_g\right).$$

and

$$\mathrm{E}_{\boldsymbol{z}_g|\boldsymbol{x}_g}[\boldsymbol{z}_g^T \boldsymbol{\Lambda}^T \boldsymbol{\Psi}^{-1} \boldsymbol{\Lambda} \boldsymbol{z}_g] = \mathrm{Tr}\left(\boldsymbol{\Lambda}^T \boldsymbol{\Psi}^{-1} \boldsymbol{\Lambda} \mathrm{E}_{\boldsymbol{z}_g|\boldsymbol{x}_g}[\boldsymbol{z}_g \boldsymbol{z}_g{}^T]\right).$$

And ignoring terms that do not depend on the expectations of $\boldsymbol{z}_g$, we obtain

$$Q(\boldsymbol{\theta}|\boldsymbol{\theta}^{(i)}) = \sum_{g=1}^G \left[-\frac{1}{2}\log|\boldsymbol{\Psi}| + \mathrm{Tr}\left(\mathrm{E}_{\boldsymbol{z}_g|\boldsymbol{x}_g}^T[\boldsymbol{z}_g] \boldsymbol{\Lambda}^T \boldsymbol{\Psi}^{-1} \boldsymbol{x}_g\right) - \frac{1}{2}\mathrm{Tr}\left(\boldsymbol{\Lambda}^T \boldsymbol{\Psi}^{-1} \boldsymbol{\Lambda} \mathrm{E}_{\boldsymbol{z}_g|\boldsymbol{x}_g}[\boldsymbol{z}_g \boldsymbol{z}_g{}^T]\right)\right].$$

We maximize $Q(\boldsymbol{\theta}|\boldsymbol{\theta}^{(i)})$ with respect to $\boldsymbol{\Lambda}$ and $\boldsymbol{\Psi}$ in turn.

Update of $\boldsymbol{\Lambda}$: We omit terms that do not depend on $\boldsymbol{\Lambda}$, and take the derivative of $Q(\boldsymbol{\theta}|\boldsymbol{\theta}^{(i)})$ with respect to $\boldsymbol{\Lambda}$, using the identities

$$\frac{\partial}{\partial \boldsymbol{\Lambda}} \mathrm{Tr}(\boldsymbol{\Lambda}^T \mathbf{A}) = \mathbf{A} \text{ and } \frac{\partial}{\partial \boldsymbol{\Lambda}} \mathrm{Tr}(\boldsymbol{\Lambda}^T \mathbf{A} \boldsymbol{\Lambda} \mathbf{B}) = \mathbf{A} \boldsymbol{\Lambda} \mathbf{B} + \mathbf{A}^T \boldsymbol{\Lambda} \mathbf{B}^T.$$

we obtain

$$\frac{\partial}{\partial \boldsymbol{\Lambda}} \mathrm{Tr}\left(\mathrm{E}_{\boldsymbol{z}_g|\boldsymbol{x}_g}^T[\boldsymbol{z}_g] \boldsymbol{\Lambda}^T \boldsymbol{\Psi}^{-1} \boldsymbol{x}_g\right) = \frac{\partial}{\partial \boldsymbol{\Lambda}} \mathrm{Tr}\left(\boldsymbol{\Lambda}^T \boldsymbol{\Psi}^{-1} \boldsymbol{x}_g \mathrm{E}_{\boldsymbol{z}_g|\boldsymbol{x}_g}^T[\boldsymbol{z}_g]\right) = \boldsymbol{\Psi}^{-1} \boldsymbol{x}_g \mathrm{E}_{\boldsymbol{z}_g|\boldsymbol{x}_g}^T[\boldsymbol{z}_g].$$

and

$$\frac{\partial}{\partial \boldsymbol{\Lambda}} \mathrm{Tr}\left(\boldsymbol{\Lambda}^T \boldsymbol{\Psi}^{-1} \boldsymbol{\Lambda} \mathrm{E}_{\boldsymbol{z}_g|\boldsymbol{x}_g}[\boldsymbol{z}_g \boldsymbol{z}_g{}^T]\right) = 2\boldsymbol{\Psi}^{-1} \boldsymbol{\Lambda} \mathrm{E}_{\boldsymbol{z}_g|\boldsymbol{x}_g}[\boldsymbol{z}_g \boldsymbol{z}_g{}^T].$$

where we use the symmetry of both $\boldsymbol{\Psi}^{-1}$ and $\mathrm{E}_{\boldsymbol{z}_g|\boldsymbol{x}_g}[\boldsymbol{z}_g \boldsymbol{z}_g{}^T]$. Therefore,

$$\frac{\partial Q(\boldsymbol{\theta}|\boldsymbol{\theta}^{(i)})}{\partial \boldsymbol{\Lambda}} = \sum_{g=1}^G \left[\boldsymbol{\Psi}^{-1} \boldsymbol{x}_g \mathrm{E}_{\boldsymbol{z}_g|\boldsymbol{x}_g}^T[\boldsymbol{z}_g] - \boldsymbol{\Psi}^{-1} \boldsymbol{\Lambda} \mathrm{E}_{\boldsymbol{z}_g|\boldsymbol{x}_g}[\boldsymbol{z}_g \boldsymbol{z}_g{}^T]\right].$$

Setting the derivative to zero yields

$$\sum_{g=1}^G \boldsymbol{x}_g \mathrm{E}_{\boldsymbol{z}_g|\boldsymbol{x}_g}^T[\boldsymbol{z}_g] = \boldsymbol{\Lambda} \sum_{g=1}^G \mathrm{E}_{\boldsymbol{z}_g|\boldsymbol{x}_g}[\boldsymbol{z}_g \boldsymbol{z}_g{}^T].$$

Therefore,

$$\boldsymbol{\Lambda}_{new} = \left(\sum_{g=1}^G \boldsymbol{x}_g \mathrm{E}_{\boldsymbol{z}_g|\boldsymbol{x}_g}^T[\boldsymbol{z}_g]\right)\left(\sum_{g=1}^G \mathrm{E}_{\boldsymbol{z}_g|\boldsymbol{x}_g}[\boldsymbol{z}_g \boldsymbol{z}_g{}^T]\right)^{-1}.$$

Under the structured latent factors, loadings, and data,

$$\boldsymbol{\Lambda} = \begin{bmatrix} \boldsymbol{\Lambda}^c & \boldsymbol{\Lambda}_u^c & 0 \\ \boldsymbol{\Lambda}^s & 0 & \boldsymbol{\Lambda}_u^s \end{bmatrix}; \boldsymbol{z}_g = \begin{bmatrix} \boldsymbol{z}_g^t \\ \boldsymbol{z}_g^c \\ \boldsymbol{z}_g^s \end{bmatrix}; \boldsymbol{x}_g = \begin{bmatrix} \boldsymbol{x}_g^c \\ \boldsymbol{x}_g^s \end{bmatrix}.$$

Substituting these block forms into the normal equation

$$\sum_{g=1}^G \boldsymbol{x}_g \mathrm{E}_{\boldsymbol{z}_g|\boldsymbol{x}_g}^T[\boldsymbol{z}_g] = \boldsymbol{\Lambda} \sum_{g=1}^G \mathrm{E}_{\boldsymbol{z}_g|\boldsymbol{x}_g}[\boldsymbol{z}_g \boldsymbol{z}_g{}^T].$$

and taking the first row block corresponding to $\boldsymbol{x}_g^c$, we obtain

$$\sum_{g=1}^G \boldsymbol{x}_g^c \mathrm{E}_{\boldsymbol{z}_g|\boldsymbol{x}_g}\left[\begin{bmatrix} \boldsymbol{z}_g^t \\ \boldsymbol{z}_g^c \\ \boldsymbol{z}_g^s \end{bmatrix}^T\right] = [\boldsymbol{\Lambda}^c \quad \boldsymbol{\Lambda}_u^c \quad 0] \sum_{g=1}^G \mathrm{E}_{\boldsymbol{z}_g|\boldsymbol{x}_g}\left[\begin{bmatrix} \boldsymbol{z}_g^t \\ \boldsymbol{z}_g^c \\ \boldsymbol{z}_g^s \end{bmatrix}\begin{bmatrix} \boldsymbol{z}_g^t \\ \boldsymbol{z}_g^c \\ \boldsymbol{z}_g^s \end{bmatrix}^T\right].$$

Comparing the column block corresponding to the shared factor $\boldsymbol{z}_g^t$ yields

$$\sum_{g=1}^{G} \boldsymbol{x}_g^c \mathrm{E}_{\boldsymbol{z}_g|\boldsymbol{x}_g}\left[(\boldsymbol{z}_g^t)^T\right] = \boldsymbol{\Lambda}^c \sum_{g=1}^{G} \mathrm{E}_{\boldsymbol{z}_g|\boldsymbol{x}_g}\left[\boldsymbol{z}_g^t(\boldsymbol{z}_g^t)^T\right] + \boldsymbol{\Lambda}_u^c \sum_{g=1}^{G} \mathrm{E}_{\boldsymbol{z}_g|\boldsymbol{x}_g}\left[\boldsymbol{z}_g^c(\boldsymbol{z}_g^t)^T\right].$$

Thus, fixing $\boldsymbol{\Lambda}_u^c$ at the previous iteration, we update $\boldsymbol{\Lambda}_{new}^c$ as

$$\boldsymbol{\Lambda}_{new}^c = (\sum_{g=1}^{G} \boldsymbol{x}_g^c \mathrm{E}_{\boldsymbol{z}_g|\boldsymbol{x}_g}\left[(\boldsymbol{z}_g^t)^T\right] - \boldsymbol{\Lambda}_u^c \sum_{g=1}^{G} \mathrm{E}_{\boldsymbol{z}_g|\boldsymbol{x}_g}\left[\boldsymbol{z}_g^c(\boldsymbol{z}_g^t)^T\right]) \left(\sum_{g=1}^{G} \mathrm{E}_{\boldsymbol{z}_g|\boldsymbol{x}_g}\left[\boldsymbol{z}_g^t(\boldsymbol{z}_g^t)^T\right]\right)^{-\mathbf{1}}.$$

Similarly, comparing the column block corresponding to $\boldsymbol{z}_g^c$ yields

$$\sum_{g=1}^{G} \boldsymbol{x}_g^c \mathrm{E}_{\boldsymbol{z}_g|\boldsymbol{x}_g}\left[(\boldsymbol{z}_g^c)^T\right] = \boldsymbol{\Lambda}^c \sum_{g=1}^{G} \mathrm{E}_{\boldsymbol{z}_g|\boldsymbol{x}_g}\left[\boldsymbol{z}_g^t(\boldsymbol{z}_g^c)^T\right] + \boldsymbol{\Lambda}_u^c \sum_{g=1}^{G} \mathrm{E}_{\boldsymbol{z}_g|\boldsymbol{x}_g}\left[\boldsymbol{z}_g^c(\boldsymbol{z}_g^c)^T\right].$$

Using the newly updated $\boldsymbol{\Lambda}_{new}^c$, we then update $\boldsymbol{\Lambda}_{u,new}^c$ as

$$\boldsymbol{\Lambda}_{u,new}^c = (\sum_{g=1}^{G} \boldsymbol{x}_g^c \mathrm{E}_{\boldsymbol{z}_g|\boldsymbol{x}_g}\left[(\boldsymbol{z}_g^c)^T\right] - \boldsymbol{\Lambda}_{new}^c \sum_{g=1}^{G} \mathrm{E}_{\boldsymbol{z}_g|\boldsymbol{x}_g}\left[\boldsymbol{z}_g^t(\boldsymbol{z}_g^c)^T\right]) \left(\sum_{g=1}^{G} \mathrm{E}_{\boldsymbol{z}_g|\boldsymbol{x}_g}\left[\boldsymbol{z}_g^c(\boldsymbol{z}_g^c)^T\right]\right)^{-\mathbf{1}}.$$

Applying the same argument to the second row block corresponding to $\boldsymbol{x}_g^s$ gives

$$\boldsymbol{\Lambda}_{new}^s = (\sum_{g=1}^{G} \boldsymbol{x}_g^s \mathrm{E}_{\boldsymbol{z}_g|\boldsymbol{x}_g}\left[(\boldsymbol{z}_g^t)^T\right] - \boldsymbol{\Lambda}_u^s \sum_{g=1}^{G} \mathrm{E}_{\boldsymbol{z}_g|\boldsymbol{x}_g}\left[\boldsymbol{z}_g^s(\boldsymbol{z}_g^t)^T\right]) \left(\sum_{g=1}^{G} \mathrm{E}_{\boldsymbol{z}_g|\boldsymbol{x}_g}\left[\boldsymbol{z}_g^t(\boldsymbol{z}_g^t)^T\right]\right)^{-\mathbf{1}}.$$

$$\boldsymbol{\Lambda}_{u,new}^s = \left(\sum_{g=1}^{G} \boldsymbol{x}_g^s \mathrm{E}_{\boldsymbol{z}_g|\boldsymbol{x}_g}\left[(\boldsymbol{z}_g^s)^T\right] - \boldsymbol{\Lambda}_{new}^s \sum_{g=1}^{G} \mathrm{E}_{\boldsymbol{z}_g|\boldsymbol{x}_g}\left[\boldsymbol{z}_g^t(\boldsymbol{z}_g^s)^T\right]\right) \left(\sum_{g=1}^{G} \mathrm{E}_{\boldsymbol{z}_g|\boldsymbol{x}_g}\left[\boldsymbol{z}_g^s(\boldsymbol{z}_g^s)^T\right]\right)^{-\mathbf{1}}.$$

These updates are obtained sequentially in a blockwise Gauss-Seidel manner: we first update $\boldsymbol{\Lambda}_{new}^c$ and $\boldsymbol{\Lambda}_{new}^s$ using $\boldsymbol{\Lambda}_u^c$ and $\boldsymbol{\Lambda}_u^s$, respectively, and then update $\boldsymbol{\Lambda}_{u,new}^c$ and $\boldsymbol{\Lambda}_{u,new}^s$ using the newly updated values $\boldsymbol{\Lambda}_{new}^c$ and $\boldsymbol{\Lambda}_{new}^s$.

Update of $\boldsymbol{\Psi}$: For the Q-function

$$Q(\boldsymbol{\theta}|\boldsymbol{\theta}^{(i)}) = \sum_{g=1}^{G} \mathrm{E}_{\boldsymbol{z}_g|\boldsymbol{x}_g}\left[-\frac{N}{2}\log 2\pi - \frac{1}{2}\log|\boldsymbol{\Psi}| - \frac{1}{2}(\boldsymbol{x}_g - \boldsymbol{\Lambda}\boldsymbol{z}_g)^T \boldsymbol{\Psi}^{-1}(\boldsymbol{x}_g - \boldsymbol{\Lambda}\boldsymbol{z}_g)\right]$$

$$= -\frac{GN}{2}\log 2\pi - \frac{G}{2}\log|\boldsymbol{\Psi}| - \frac{1}{2}\sum_{g=1}^{G} \mathrm{E}_{\boldsymbol{z}_g|\boldsymbol{x}_g}\left[(\boldsymbol{x}_g - \boldsymbol{\Lambda}\boldsymbol{z}_g)^T \boldsymbol{\Psi}^{-1}(\boldsymbol{x}_g - \boldsymbol{\Lambda}\boldsymbol{z}_g)\right].$$

Using the trace identity $\boldsymbol{a}^T\mathbf{B}\boldsymbol{a} = \mathbf{Tr}(\mathbf{B}\boldsymbol{a}\boldsymbol{a}^T)$, and dropping the constant term, we obtain

$$Q(\boldsymbol{\theta}|\boldsymbol{\theta}^{(i)}) = -\frac{G}{2}\log|\boldsymbol{\Psi}| - \frac{1}{2}\sum_{g=1}^{G} \mathrm{Tr}\left(\boldsymbol{\Psi}^{-1}\mathrm{E}_{\boldsymbol{z}_g|\boldsymbol{x}_g}\left[(\boldsymbol{x}_g - \boldsymbol{\Lambda}\boldsymbol{z}_g)(\boldsymbol{x}_g - \boldsymbol{\Lambda}\boldsymbol{z}_g)^T\right]\right).$$

We use the standard matrix derivative identities for symmetric positive definite $\boldsymbol{\Psi}$

$$\frac{\partial}{\partial\boldsymbol{\Psi}}\log|\boldsymbol{\Psi}| = \boldsymbol{\Psi}^{-1}, \frac{\partial}{\partial\boldsymbol{\Psi}}\mathrm{Tr}(\boldsymbol{\Psi}^{-1}\mathbf{A}) = -\boldsymbol{\Psi}^{-1}\mathbf{A}\boldsymbol{\Psi}^{-1}.$$

where $\mathbf{A}$ is any symmetric matrix.

Taking the derivative of $Q(\boldsymbol{\theta}|\boldsymbol{\theta}^{(i)})$ with respect to $\boldsymbol{\Psi}$, we obtain

$$\frac{\partial Q(\boldsymbol{\theta}|\boldsymbol{\theta}^{(i)})}{\partial\boldsymbol{\Psi}} = -\frac{G}{2}\boldsymbol{\Psi}^{-1} + \frac{1}{2}\boldsymbol{\Psi}^{-1}\left[\sum_{g=1}^{G} \mathrm{E}_{\boldsymbol{z}_g|\boldsymbol{x}_g}\left[(\boldsymbol{x}_g - \boldsymbol{\Lambda}\boldsymbol{z}_g)(\boldsymbol{x}_g - \boldsymbol{\Lambda}\boldsymbol{z}_g)^T\right]\right]\boldsymbol{\Psi}^{-1}.$$

Setting $\frac{\partial Q(\boldsymbol{\theta}|\boldsymbol{\theta}^{(i)})}{\partial\boldsymbol{\Psi}} = 0$, we obtain

$$G\boldsymbol{\Psi}^{-1} = \boldsymbol{\Psi}^{-1}\left[\sum_{g=1}^{G} \mathrm{E}_{\boldsymbol{z}_g|\boldsymbol{x}_g}\left[(\boldsymbol{x}_g - \boldsymbol{\Lambda}\boldsymbol{z}_g)(\boldsymbol{x}_g - \boldsymbol{\Lambda}\boldsymbol{z}_g)^T\right]\right]\boldsymbol{\Psi}^{-1}.$$

Left-multiplying both sides by $\boldsymbol{\Psi}$ and right-multiplying both sides by $\boldsymbol{\Psi}$ yields

$$G\boldsymbol{\Psi} = \sum_{g=1}^{G} \mathrm{E}_{\boldsymbol{z}_g|\boldsymbol{x}_g}\left[(\boldsymbol{x}_g - \boldsymbol{\Lambda}\boldsymbol{z}_g)(\boldsymbol{x}_g - \boldsymbol{\Lambda}\boldsymbol{z}_g)^T\right].$$

and hence the M-step update

$$\boldsymbol{\Psi} = \frac{1}{G}\sum_{g=1}^{G} \mathrm{E}_{\boldsymbol{z}_g|\boldsymbol{x}_g}\left[\left(\boldsymbol{x}_g - \boldsymbol{\Lambda}\boldsymbol{z}_g\right)\left(\boldsymbol{x}_g - \boldsymbol{\Lambda}\boldsymbol{z}_g\right)^T\right].$$

Expanding the quadratic term gives

$$\boldsymbol{\Psi} = \frac{1}{G}\sum_{g=1}^{G} \mathrm{E}_{\boldsymbol{z}_g|\boldsymbol{x}_g}\left[\boldsymbol{x}_g\boldsymbol{x}_g^T - \boldsymbol{\Lambda}\boldsymbol{z}_g\boldsymbol{x}_g^T - \boldsymbol{x}_g\boldsymbol{z}_g^T\boldsymbol{\Lambda}^T + \boldsymbol{\Lambda}\boldsymbol{z}_g\boldsymbol{z}_g^T\boldsymbol{\Lambda}^T\right]$$

$$= \frac{1}{G}\sum_{g=1}^{G}\left[\boldsymbol{x}_g\boldsymbol{x}_g^T - \boldsymbol{\Lambda}\mathrm{E}_{\boldsymbol{z}_g|\boldsymbol{x}_g}[\boldsymbol{z}_g]\boldsymbol{x}_g^T - \boldsymbol{x}_g\mathrm{E}_{\boldsymbol{z}_g|\boldsymbol{x}_g}^T[\boldsymbol{z}_g]\boldsymbol{\Lambda}^T + \boldsymbol{\Lambda}\mathrm{E}_{\boldsymbol{z}_g|\boldsymbol{x}_g}[\boldsymbol{z}_g\boldsymbol{z}_g^T]\boldsymbol{\Lambda}^T\right].$$

In our implementation, $\boldsymbol{\Psi}$ is constrained to be diagonal. We approximate its update by taking the diagonal part of the aggregated residual covariance, i.e.,

$$\boldsymbol{\Psi}_{new} = \frac{1}{G}\mathrm{diag}\left(\sum_{g=1}^{G}\boldsymbol{x}_g{\boldsymbol{x}_g}^T - \boldsymbol{\Lambda}_{new}\mathrm{E}_{\boldsymbol{z}_g|\boldsymbol{x}_g}[\boldsymbol{z}_g]{\boldsymbol{x}_g}^T\right).$$

We then form $\mathbf{W}$ by concatenating the two data matrices, $\mathbf{W} = [\boldsymbol{X}^c\ \boldsymbol{X}^s]$, so that for each gene g, $\boldsymbol{x}_g = {\boldsymbol{w}_g}^T$ is the transpose of the g-th row of $\mathbf{W}$.

Finally, we apply a small positive floor to the diagonal entries of $\boldsymbol{\Psi}$ to ensure the numerical stability of $\boldsymbol{\Psi}^{-1}$. Convergence is monitored by the change in the model-implied low-rank covariance matrix:

$$\|\boldsymbol{\Lambda}\boldsymbol{\Lambda}^T - \boldsymbol{\Lambda}_{new}\boldsymbol{\Lambda}_{new}^T\|_F^2 < \varepsilon.$$

evaluated every 10 iterations after a burn-in period (100 iterations).

1.2. Performance Evaluation Metrics

1.2.1. Adjusted Rand Index (ARI)

ARI compares the overlap between two partitions of cells, here the Leiden clustering result and the known cell-type labels. ARI is 1 for perfect agreement and has an expected value of 0 for random labeling; negative values may occur when the agreement is worse than random. ARI is calculated using the formula:

$$\mathrm{ARI}(P,T) = \frac{\sum_{ls}\binom{n_{ls}}{2} - \left[\sum_l\binom{n_l}{2}\sum_s\binom{n_s}{2}\right]/\binom{n}{2}}{\left[\sum_l\binom{n_l}{2} + \sum_s\binom{n_s}{2}\right]/2 - \left[\sum_l\binom{n_l}{2}\sum_s\binom{n_s}{2}\right]/\binom{n}{2}}$$

1.2.2. Normalized Mutual Information (NMI)

In this study, NMI is used to compare the cell type labels with the results obtained from Leiden clustering of integrated data. NMI measures the similarity between cell type labels and clustering results, with values ranging from 0 to 1, where 1 indicates a perfect match between the two clustering results, and 0 indicates no correlation.

Let P be the Leiden clustering results and T be the known cell type labels. The mutual information (MI) between P and T is:

$$\mathrm{MI}(P,T) = \sum_l \sum_s \frac{n_{ls}}{n} log\left(\frac{\frac{n_{ls}}{n}}{\frac{n_l n_s}{n^2}}\right)$$

where n is the total number of cells; n_l is the number of cells in the l-th Leiden cluster ($l = 1,2,\cdots,r$); n_s is the number of cells in the s-th cluster of the known cell type labels T ($s = 1,2,\cdots,k$); and n_{ls} represents the overlap between the l-th Leiden cluster and the s-th cell type cluster.

Definitions of n_l, n_s, and n_{ls} are:

$$n_l = \sum_i^n I\{p_i = l\}$$

$$n_s = \sum_i^n I\{t_i = s\}$$

$$n_{ls} = \sum_i^n I\{p_i = l\} I\{t_i = s\}$$

The entropy for P and T is defined as follows:

$$H(P) = -\sum_l \frac{n_l}{n} \, log\left(\frac{n_l}{n}\right)$$

$$H(T) = -\sum_s \frac{n_s}{n} \, log\left(\frac{n_s}{n}\right)$$

Using entropy and mutual information, we define NMI as:

$$\mathrm{NMI}(P,T) = \frac{\mathrm{MI}(P,T)}{\sqrt{H(P)H(T)}}$$

1.2.3. Average silhouette width (ASW)

Average silhouette width (ASW) was used to evaluate both cell-type separation and batch mixing in the integrated representation. For each cell i, the silhouette coefficient was calculated as follows:

$$s(i) = \frac{b(i) - a(i)}{\max(a(i), b(i))}.$$

Here, $a(i)$ is the average distance from cell i to all other cells in the same cluster and $b(i)$ is the minimum average distance from cell i to cells with a different label. The ASW was calculated as the average silhouette coefficient across all cells:

$$\mathrm{ASW} = \frac{1}{N} \sum_{i=1}^{N} s(i),$$

where N is the total number of cells.

For biological conservation, cell-type ASW (ASWct) was calculated using known cell-type labels and rescaled to the range [0, 1]:

$$\text{ASWct} = \frac{\text{ASW(cell type)}+1}{2}.$$

Higher ASWct indicates better separation of known cell-type labels in the integrated representation.

For batch correction, batch ASW (ASWbt) was calculated using batch labels within each cell type and then averaged across cell types. It was transformed as:

$$\text{ASWbt} = 1 - |\text{ASW(batch)}|.$$

Higher ASWbt indicates better batch mixing, because batch labels are less separable in the integrated representation.

Both metrics were computed using the embeddings provided by integration methods or the PCA representations of feature outputs.

1.2.4. Local inverse Simpson's index (LISI)

Local inverse Simpson's index (LISI) was used to quantify local label diversity in the integrated representation. For each cell i, the local distribution of labels was estimated from its neighborhood graph. If p_{ij} denotes the local proportion of label j around cell i, the LISI score was defined as:

$$\text{LISI}_i = \frac{1}{\sum_j p_{ij}^2}.$$

We used two LISI-based metrics: integration LISI(iLISI) and cell-type LISI(cLISI). iLISI was used to evaluate batch mixing. Higher raw iLISI values indicate better local mixing of batches. In contrast, cLISI was used to evaluate cell-type conservation. Lower raw cLISI values indicate better preservation of cell-type separation, because cells from the same cell type should remain locally grouped after integration.

To make the metrics comparable with other evaluation scores, LISI values were scaled to the range [0,1]. For B batches and C cell types, the scaled scores were defined as:

$$\text{iLISI}_{\text{scaled}} = \frac{\text{iLISI}-1}{B-1},$$

$$\text{cLISI}_{\text{scaled}} = 1 - \frac{\text{cLISI}-1}{C-1},$$

Thus, higher $\text{iLISI}_{\text{scaled}}$ indicates better batch mixing, whereas higher $\text{cLISI}_{\text{scaled}}$ indicates better cell-type conservation. In the comprehensive evaluation score, the scaled LISI scores were used.

1.2.5 Graph Connectivity

Graph connectivity measures whether cells with the same identity label remain connected in the integrated k-nearest-neighbor graph. For each cell identity label c, a subgraph $G_c(N_c; E_c)$ was extracted from the integrated k-nearest-neighbor graph, containing only cells with label c. The graph connectivity score was calculated as:

$$GC = \frac{1}{|C|}\sum_{c\epsilon C}\frac{|LCC(G_c)|}{|N_c|}$$

where C is the set of cell identity labels, N_c is the set of cells with identity label c, and $LCC(G_c)$ denotes the largest connected component of G_c. The score ranges from 0 to 1, with higher values indicating better connectivity of cells with the same identity across batches.

1.2.6. Comprehensive Integration Evaluation Score

avg bio is the average of biological conservation metrics, calculated by averaging ARI, NMI, ASWct, and cLISI. This metric reflects the separation of cell types and how well the integrated data matches the true labels. avg batch is the average of batch effect removal metrics, calculated by averaging Graph connectivity, ASWbt, and iLISI. This metric assesses how well different batches are integrated and the extent of batch effect removal. T$otal$ is the combined score, calculated as a weighted average of avg bio (60%) and avg batch (40%). The formula is:

$$\mathrm{T}otal = 0.6 \times \text{avg bio} + 0.4 \times \text{avg batch}$$

This metric provides an overall evaluation by considering both biological conservation and batch effect removal.[3]

1.3. Survival Prediction Evaluation Metrics

Let T_i denote the observed follow-up time for subject i, $\delta_i \in \{0,1\}$ the event indicator, and $\eta_i = \boldsymbol{x}_i^T\widehat{\boldsymbol{\beta}}$ the fitted risk score, where $\boldsymbol{x}_i$ is the covariate vector and $\widehat{\boldsymbol{\beta}}$ is the estimated coefficient vector. Let $\widehat{\boldsymbol{S}}(t|\boldsymbol{x}_i)$ denote the predicted survival probability for subject i at time t. We evaluated survival prediction performance using the following metrics.

1.3.1 Significant Gene Ratio

For each gene g, the Wald statistic is defined as

$$z_g = \frac{\hat{\beta}_g}{\mathrm{SE}(\hat{\beta}_g)},$$

with corresponding two-sided p-value

$$p_g = 2\{1 - \Phi(|\mathrm{z_g}|)\},$$

where $\Phi(\cdot)$ denotes the cumulative distribution function of the standard normal distribution. The significant gene ratio is then defined as

$$\mathrm{SGR} = \frac{1}{p}\sum_{g=1}^{p} \mathbb{1}(p_g < 0.05),$$

where p is the number of genes included in the fitted Cox proportional hazards model.

1.3.2 Concordance Index (C-index)

Let $\mathrm{P} = \{(i,j): T_i < T_j,\ \delta_i = 1\}$ denote the set of comparable pairs. The C-index is defined as

$$C = \frac{1}{|\mathrm{P}|}\sum_{(i,j)\in\mathrm{P}} [\,\mathbb{1}(\eta_i > \eta_j) + \frac{1}{2}\mathbb{1}(\eta_i = \eta_j)],$$

where $\mathbb{1}(\cdot)$ is the indicator function.

1.3.3 Akaike Information Criterion (AIC)

AIC was computed from the Cox partial likelihood:

$$\mathrm{AIC} = -2\log L_p(\hat{\boldsymbol{\beta}}) + 2k,$$

where

$$L_p(\boldsymbol{\beta}) = \prod_{i:\delta_i=1} \frac{\exp(\boldsymbol{x}_i^T\boldsymbol{\beta})}{\sum_{j\in R(T_i)}\exp(\boldsymbol{x}_j^T\boldsymbol{\beta})},$$

and $R(T_i) = \{j: T_j \geq T_i\}$ is the risk set at time T_i, and k is the number of fitted coefficients.

1.3.4 Brier score

At evaluation time t, the Brier score is defined as

$$\mathrm{BS}(t) = \frac{1}{n}\sum_{i=1}^{n}\left(\mathbb{1}(T_i > t) - \hat{S}(t|\boldsymbol{x}_i)\right)^2,$$

which measures the squared difference between the observed survival status at time t and the predicted survival probability.

For right-censored data, a censoring-adjusted IPCW form can be used:

$$BS(t) = \frac{1}{n}\sum_{i=1}^{n}\omega_i(t)\left(\mathbb{1}(T_i > t) - \hat{S}(t|x_i)\right)^2,$$

where $\omega_i(t)$ is the inverse probability of censoring weight, estimated from the censoring distribution.

1.3.5 Log-rank p-value

To assess the survival difference between groups, we used the log-rank test. The corresponding *p*-value was reported as the log-rank *p*-value.

2. Supporting Figures

Figure S1. Supplementary analysis of the simulation.

(**A-C**) Ablation and sensitivity analysis of CAPER across high-, confounded-, and low-SNR scenarios. CAPER variants with different latent dimensions show stable performance, while the correction step modestly improves batch-correction-related metrics in some settings.

(**D**) CAPER-based recovery of graded cell-population-specific responses across SNR settings. The simulated response hierarchy was designed as CP1 > CP2 > CP3 > CP4, and CAPER recovered this expected pattern more clearly as SNR increased.

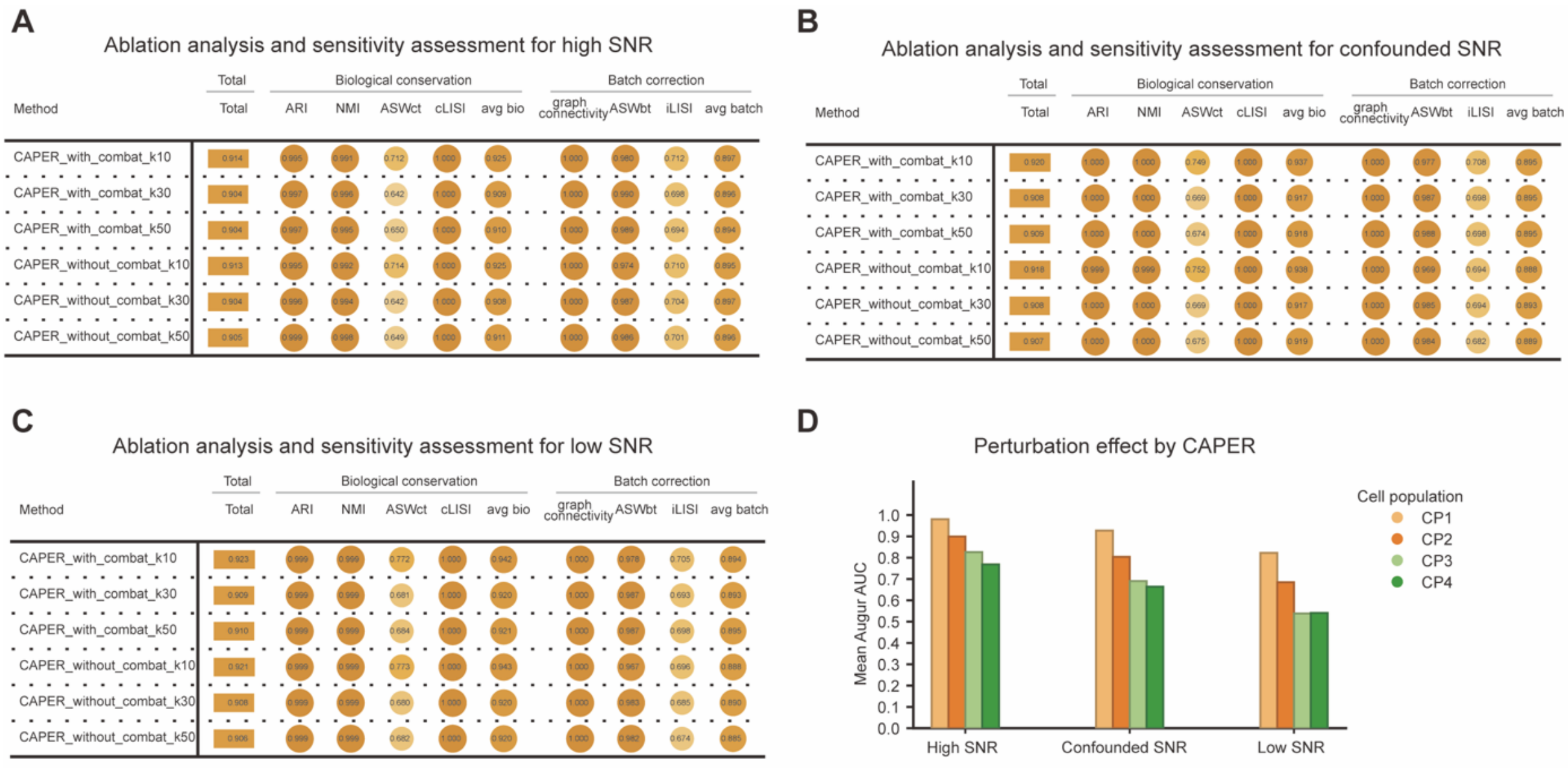
A
Ablation analysis and sensitivity assessment for high SNR
B
Ablation analysis and sensitivity assessment for confounded SNR
C
Ablation analysis and sensitivity assessment for low SNR
Method
Total
Biological conservation
Batch correction
ARI
NMI
ASWct
cLISI
avg bio
graph connectivity
ASWbt
iLISI
avg batch
CAPER_with_combat_k10
CAPER_with_combat_k30
CAPER_with_combat_k50
CAPER_without_combat_k10
CAPER_without_combat_k30
CAPER_without_combat_k50
D
Perturbation effect by CAPER
Mean Augur AUC
1.0
0.9
0.8
0.7
0.6
0.5
0.4
0.3
0.2
0.1
0.0
High SNR
Confounded SNR
Low SNR
Cell population
CP1
CP2
CP3
CP4

Figure S2. Supplementary analysis of the PBMC dataset.

(**A**) UMAP visualization of CAPER condition-specific latent factors. Low-dimensional embeddings of the condition-specific latent factors extracted by CAPER are shown, with cells colored by experimental condition (left) and cell type (right). The clear separation between stimulated and control cells in responsive populations demonstrates CAPER's ability to isolate perturbation-specific unwanted variation.

(B) Overlap of upregulated DE genes in CD14+ monocytes. Venn diagram shows shared and component-specific upregulated DE genes identified from CAPER reconstruction and the unwanted-variation component.

(C) GO enrichment analysis of component-specific upregulated DE genes in CD14+ monocytes. The bar plot shows the top 5 GO terms enriched among CAPER reconstruction-specific and unwanted variation-specific upregulated DE genes. Compared with unwanted variation-specific genes, CAPER reconstruction-specific genes were more strongly enriched for immune defense and interferon-related response programs, indicating that CAPER reconstruction preferentially retained biologically relevant IFN-β-induced signals.

(**D**) GO enrichment analysis of CAPER's top PC loadings. Bar plots display the top 5 Gene Ontology (GO) terms enriched among genes with the most positive and most negative loadings on the first three principal components identified by CAPER. These terms reflect the primary biological processes driving variation in the corrected data, with strong enrichment for immune activation, antigen-presentation, and antiviral-response programs.

(**E**) GO enrichment analysis of CellANOVA's top PC loadings. Bar plots show the top 5 GO terms enriched among genes with the most positive and most negative loadings on the first three principal components identified by CellANOVA. The enrichment patterns differ from CAPER, reflecting methodological differences in signal recovery.

(**F**) Cell-type transcriptional concordance analysis. Heatmaps show pairwise Kendall's τ correlation coefficients between gene expression rankings for each cell type, derived from the corrected expression matrices and global Kendall's W concordance across all cell types. CAPER, CellANOVA and scGen capture known biological differences between myeloid and lymphoid lineages, reflected in lower cross-cell-type concordance. In contrast, scDisInFact, ComBat yield uniformly high concordance across all cell types, indicating a loss of cell-type-specific biological heterogeneity.

(**G**) GO enrichment analysis of co-expression module hub genes. Bar plots show enriched GO terms for hub genes from ME1, ME2, and ME3, supporting the biological interpretation of CAPER-derived co-expression modules.

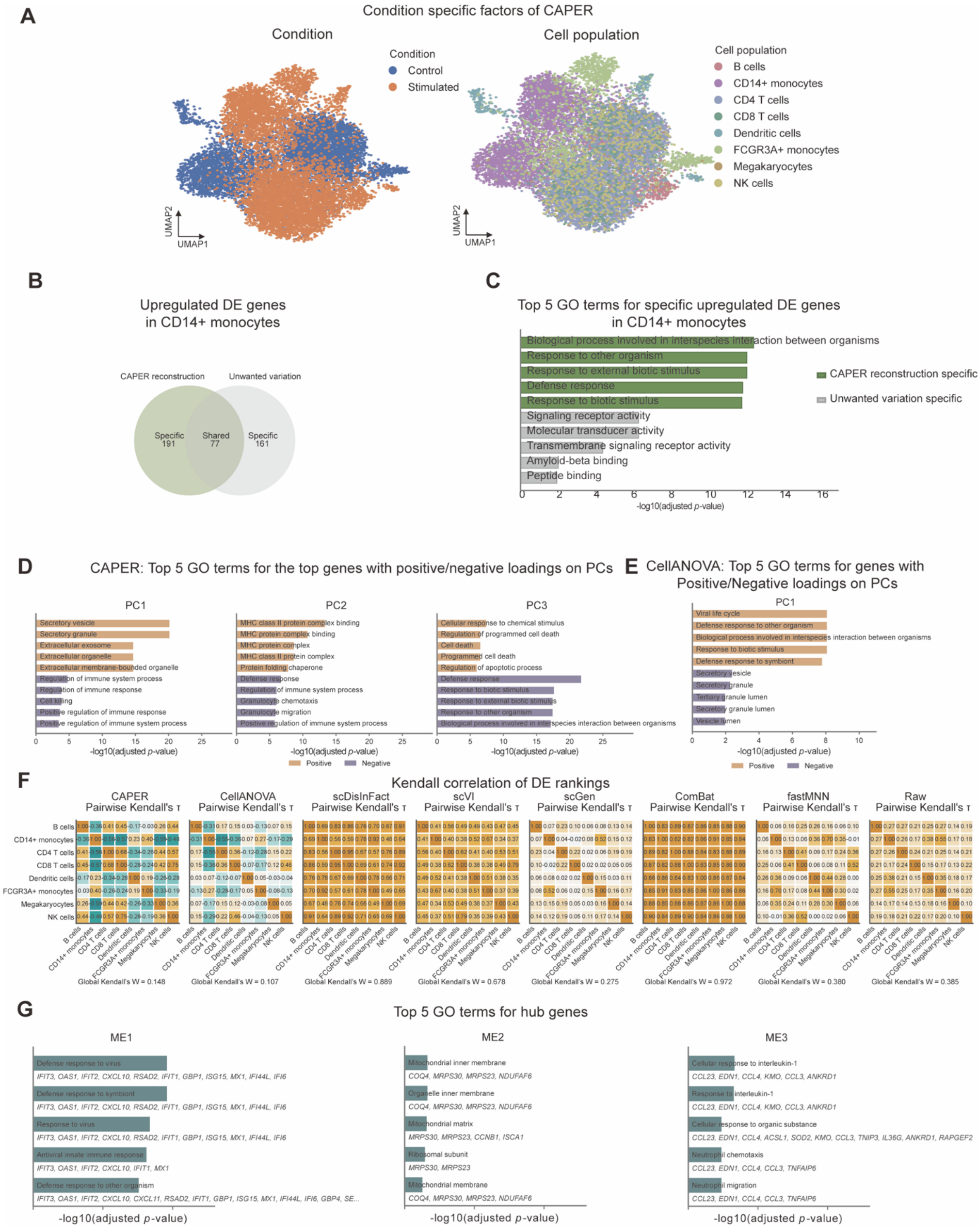
A
Condition specific factors of CAPER
Condition
Cell population
Condition
Control
Stimulated
Cell population
B cells
CD14+ monocytes
CD4 T cells
CD8 T cells
Dendritic cells
FCGR3A+ monocytes
Megakaryocytes
NK cells
B
Upregulated DE genes
in CD14+ monocytes
CAPER reconstruction
Unwanted variation
C
Top 5 GO terms for specific upregulated DE genes
in CD14+ monocytes
CAPER reconstruction specific
Unwanted variation specific
-log10(adjusted p-value)
D
CAPER: Top 5 GO terms for the top genes with positive/negative loadings on PCs
E
CellANOVA: Top 5 GO terms for genes with Positive/Negative loadings on PCs
F
Kendall correlation of DE rankings
G
Top 5 GO terms for hub genes
ME1
ME2
ME3

Figure S3. Supplementary analysis of the LUAD dataset.

(**A**) UMAP visualization of CAPER condition-specific latent factors. Low-dimensional embeddings of the condition-specific latent factors extracted by CAPER are shown, with cells colored by condition (tumor vs. control; left) and cell type (right), showing that CAPER condition-specific factors capture variation independent of core cell identity.

(**B**) Overlap of upregulated DE genes in epithelial cells. Venn diagram shows shared and component-specific upregulated DE genes identified from CAPER reconstruction and the unwanted-variation component in epithelial cells.

(**C**) GO enrichment analysis of component-specific upregulated DE genes in epithelial cells. The bar plot shows the top 5 GO terms enriched among CAPER reconstruction-specific and unwanted variation-specific upregulated DE genes. Compared with unwanted variation-specific genes, CAPER reconstruction-specific genes showed stronger enrichment for epithelial cell-state programs related to lung epithelial biology and tumor-associated remodeling, suggesting that CAPER reconstruction preferentially retained LUAD-relevant epithelial signals.

(**D**) GO enrichment analysis of CAPER's top PC loadings. Bar plots display the top 5 GO terms enriched among genes with the most positive and most negative loadings on the first three principal components identified by CAPER. These terms reflect the primary biological processes driving tumor-associated variation, with enrichment for antigen presentation, immune activation, and tumor microenvironment-related programs.

(**E**) GO enrichment analysis of CellANOVA's top PC loadings. Bar plots show the top 5 GO terms enriched among genes with the most positive and most negative loadings on the first three principal components identified by CellANOVA. While some cancer-relevant terms are captured, the enrichment patterns differ from CAPER, reflecting methodological differences in signal recovery and highlighting distinct biological processes prioritized by each method.

(**F**) Kendall correlation of DE gene rankings across cell populations. Heatmaps show pairwise Kendall's τ values and global Kendall's W for each method, evaluating preservation of cell-type-specific tumor-response heterogeneity. CAPER captures known biological differences in tumor-associated responses among distinct cell populations, reflected in intermediate cross-cell-type concordance. In contrast, ComBat yields uniformly high concordance across cell types, suggesting smoothing or homogenization of tumor-response patterns and reduced preservation of cell-type-specific biological heterogeneity.

(**G**) Overlap of downregulated DE genes in epithelial cells. The UpSet plot summarizes shared and method-specific downregulated DE genes across CAPER and compared methods.

(**H**) GO enrichment of method-specific downregulated DE genes. Bubble plots show enriched GO terms among method-specific downregulated DE genes in epithelial cells.

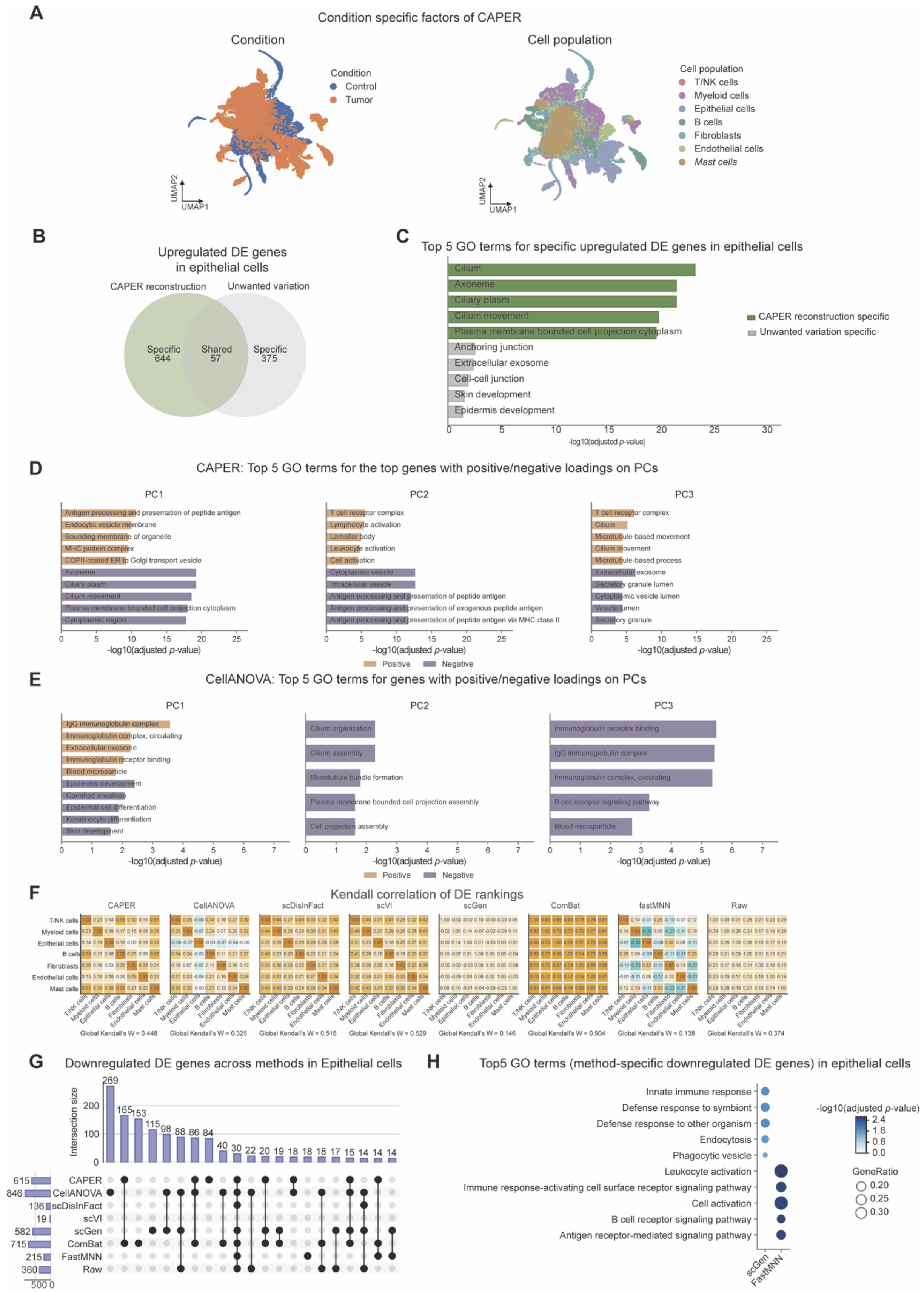
A
Condition specific factors of CAPER
Condition
Condition
Control
Tumor
UMAP2
UMAP1
Cell population
Cell population
T/NK cells
Myeloid cells
Epithelial cells
B cells
Fibroblasts
Endothelial cells
Mast cells
B
Upregulated DE genes in epithelial cells
CAPER reconstruction
Unwanted variation
Specific 644
Shared 57
Specific 375
C
Top 5 GO terms for specific upregulated DE genes in epithelial cells
Cilium
Axoneme
Ciliary plasm
Cilium movement
Plasma membrane bounded cell projection cytoplasm
Anchoring junction
Extracellular exosome
Cell-cell junction
Skin development
Epidermis development
CAPER reconstruction specific
Unwanted variation specific
-log10(adjusted p-value)
D
CAPER: Top 5 GO terms for the top genes with positive/negative loadings on PCs
PC1
PC2
PC3
Positive
Negative
E
CellANOVA: Top 5 GO terms for genes with positive/negative loadings on PCs
F
Kendall correlation of DE rankings
CAPER
CellANOVA
scDisInFact
scVI
scGen
ComBat
fastMNN
Raw
Global Kendall's W = 0.448
Global Kendall's W = 0.325
Global Kendall's W = 0.516
Global Kendall's W = 0.529
Global Kendall's W = 0.146
Global Kendall's W = 0.904
Global Kendall's W = 0.138
Global Kendall's W = 0.374
G
Downregulated DE genes across methods in Epithelial cells
Intersection size
H
Top5 GO terms (method-specific downregulated DE genes) in epithelial cells
Innate immune response
Defense response to symbiont
Defense response to other organism
Endocytosis
Phagocytic vesicle
Leukocyte activation
Immune response-activating cell surface receptor signaling pathway
Cell activation
B cell receptor signaling pathway
Antigen receptor-mediated signaling pathway
-log10(adjusted p-value)
GeneRatio
scGen
FastMNN

Figure S4. Supplementary analysis of T1D dataset.

(**A**) UMAP visualization of CAPER condition-specific latent factors. Low-dimensional embeddings of the condition-specific latent factors extracted by CAPER are shown, with cells colored by condition (T1D vs. control; left) and cell type (right), showing that CAPER condition-specific factors capture variation independent of core pancreatic cell identity and condition-associated biological signals.

(**B**) Overlap of upregulated DE genes in type B pancreatic cells. Venn diagram shows shared and component-specific upregulated DE genes identified from CAPER reconstruction and the unwanted-variation component in type B pancreatic cells.

(**C**) GO enrichment analysis of component-specific upregulated DE genes in type B pancreatic cells. The bar plot shows the top 5 GO terms enriched among CAPER reconstruction-specific and unwanted variation-specific upregulated DE genes. Compared with unwanted variation-specific genes, CAPER reconstruction-specific genes were more closely associated with β-cell secretory and vesicle/exocytosis-related programs, supporting that CAPER reconstruction preferentially preserved biologically relevant T1D-associated β-cell functional signals.

(**D**) GO enrichment analysis of CAPER's top PC loadings. Bar plots display the top 5 Gene Ontology (GO) terms enriched among genes with the most positive and most negative loadings on the first three principal components identified by CAPER. These terms reflect the primary biological processes driving T1D-associated variation, with enrichment for extracellular matrix, endoplasmic reticulum, transport vesicle, and secretory vesicle programs.

(**E**) GO enrichment analysis of CellANOVA's top PC loadings. Bar plots show the top 5 GO terms enriched among genes with the most positive and most negative loadings on the first three principal components identified by CellANOVA. While some diabetes-relevant terms are captured, the enrichment patterns differ from CAPER, reflecting methodological differences in signal recovery and highlighting distinct biological processes prioritized by each method in this challenging dataset.

(**F**) Kendall correlation of DE gene rankings across cell populations. Heatmaps show pairwise Kendall's τ values and global Kendall's W for each method, evaluating preservation of cell-type-specific T1D-response heterogeneity. CAPER captures known biological differences between endocrine and non-endocrine pancreatic lineages, reflected in lower cross-cell-type concordance. ComBat shows uniformly high concordance across cell types, whereas scVI and scDisInFact show higher global concordance than CAPER, suggesting reduced preservation of cell-type-specific T1D-response heterogeneity.

(**G**) Functional enrichment of method-unique DE genes. Bubble plots show enriched GO terms among genes uniquely identified by compared methods relative to CAPER, highlighting broader secretion- or mitochondrion-related processes.

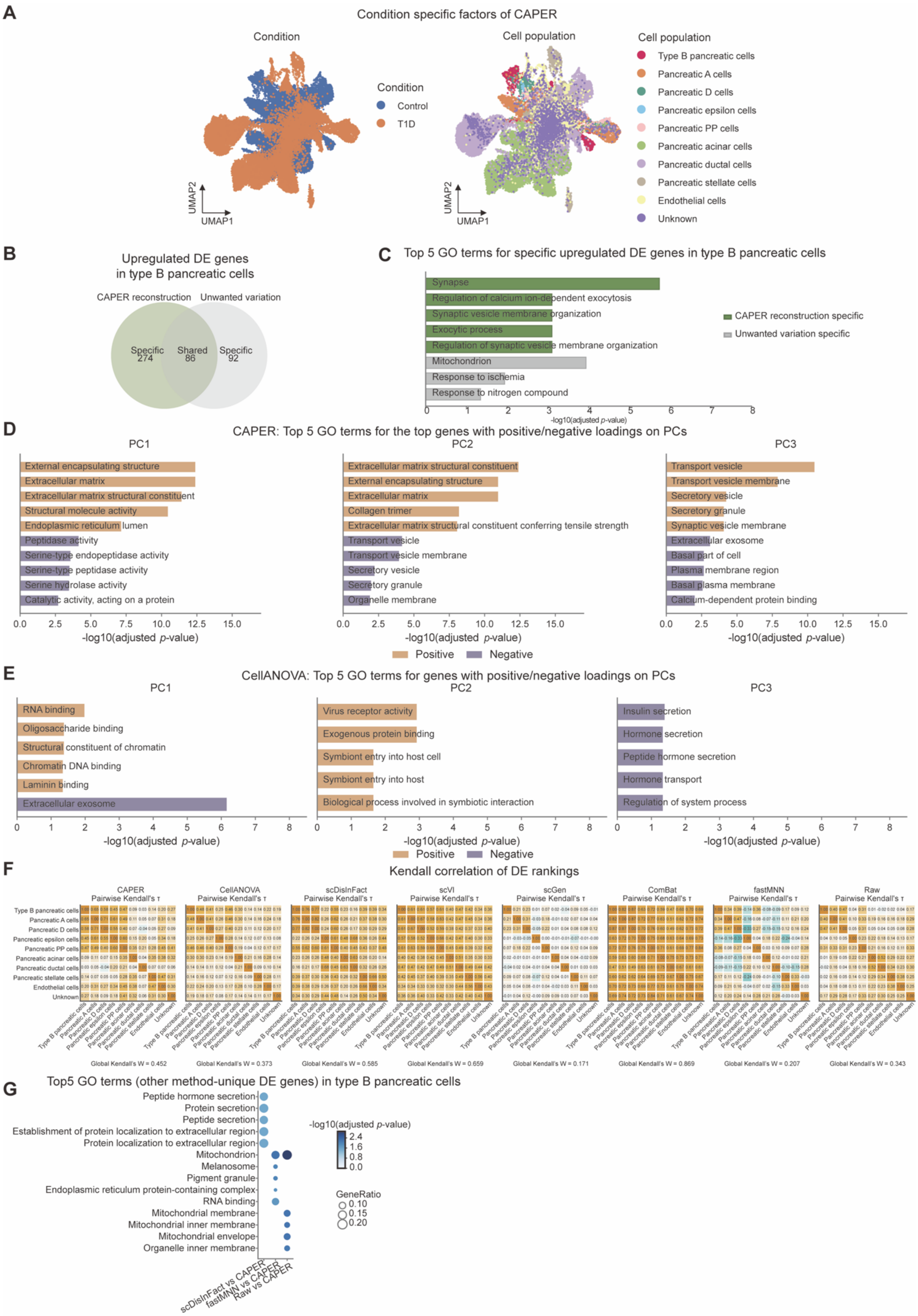
A
Condition specific factors of CAPER
Condition
Cell population
Condition
Control
T1D
Cell population
Type B pancreatic cells
Pancreatic A cells
Pancreatic D cells
Pancreatic epsilon cells
Pancreatic PP cells
Pancreatic acinar cells
Pancreatic ductal cells
Pancreatic stellate cells
Endothelial cells
Unknown
UMAP1
UMAP2
B
Upregulated DE genes
in type B pancreatic cells
CAPER reconstruction
Unwanted variation
Specific 274
Shared 86
Specific 92
C
Top 5 GO terms for specific upregulated DE genes in type B pancreatic cells
Synapse
Regulation of calcium ion-dependent exocytosis
Synaptic vesicle membrane organization
Exocytic process
Regulation of synaptic vesicle membrane organization
Mitochondrion
Response to ischemia
Response to nitrogen compound
CAPER reconstruction specific
Unwanted variation specific
-log10(adjusted p-value)
D
CAPER: Top 5 GO terms for the top genes with positive/negative loadings on PCs
PC1
External encapsulating structure
Extracellular matrix
Extracellular matrix structural constituent
Structural molecule activity
Endoplasmic reticulum lumen
Peptidase activity
Serine-type endopeptidase activity
Serine-type peptidase activity
Serine hydrolase activity
Catalytic activity, acting on a protein
PC2
Extracellular matrix structural constituent
External encapsulating structure
Extracellular matrix
Collagen trimer
Extracellular matrix structural constituent conferring tensile strength
Transport vesicle
Transport vesicle membrane
Secretory vesicle
Secretory granule
Organelle membrane
PC3
Transport vesicle
Transport vesicle membrane
Secretory vesicle
Secretory granule
Synaptic vesicle membrane
Extracellular exosome
Basal part of cell
Plasma membrane region
Basal plasma membrane
Calcium-dependent protein binding
-log10(adjusted p-value)
Positive
Negative
E
CellANOVA: Top 5 GO terms for genes with positive/negative loadings on PCs
PC1
RNA binding
Oligosaccharide binding
Structural constituent of chromatin
Chromatin DNA binding
Laminin binding
Extracellular exosome
PC2
Virus receptor activity
Exogenous protein binding
Symbiont entry into host cell
Symbiont entry into host
Biological process involved in symbiotic interaction
PC3
Insulin secretion
Hormone secretion
Peptide hormone secretion
Hormone transport
Regulation of system process
-log10(adjusted p-value)
Positive
Negative
F
Kendall correlation of DE rankings
CAPER Pairwise Kendall's τ
CellANOVA Pairwise Kendall's τ
scDisInFact Pairwise Kendall's τ
scVI Pairwise Kendall's τ
scGen Pairwise Kendall's τ
ComBat Pairwise Kendall's τ
fastMNN Pairwise Kendall's τ
Raw Pairwise Kendall's τ
Global Kendall's W = 0.452
Global Kendall's W = 0.373
Global Kendall's W = 0.585
Global Kendall's W = 0.659
Global Kendall's W = 0.171
Global Kendall's W = 0.869
Global Kendall's W = 0.207
Global Kendall's W = 0.343
G
Top5 GO terms (other method-unique DE genes) in type B pancreatic cells
Peptide hormone secretion
Protein secretion
Peptide secretion
Establishment of protein localization to extracellular region
Protein localization to extracellular region
Mitochondrion
Melanosome
Pigment granule
Endoplasmic reticulum protein-containing complex
RNA binding
Mitochondrial membrane
Mitochondrial inner membrane
Mitochondrial envelope
Organelle inner membrane
scDisInFact vs CAPER
fastMNN vs CAPER
Raw vs CAPER
-log10(adjusted p-value)
GeneRatio
0.10
0.15
0.20

Figure S5. Trajectory inference and ROS pathway activity analysis.

(A-G) Trajectory inference and ROS pathway activity analysis in type B pancreatic cells across compared methods, including CellANOVA, scDisInFact, scVI, scGen, ComBat, fastMNN, and Raw. For each method, UMAP plots show cells colored by condition and diffusion pseudotime, followed by T1D-control pseudotime comparison and ROS pathway activity along pseudotime. This analysis evaluates whether each method preserves T1D-associated β-cell state progression and its association with oxidative-stress pathway activation.

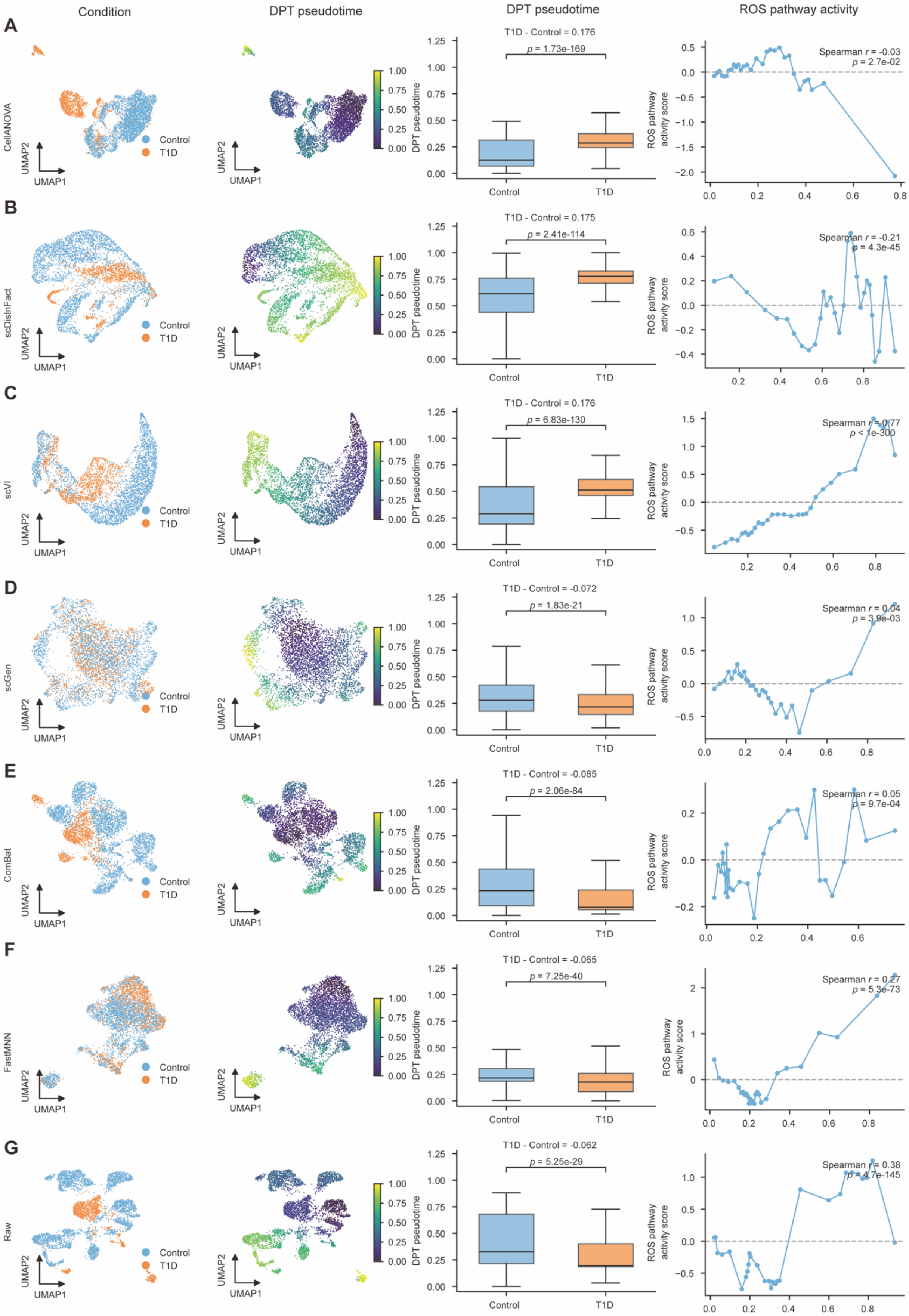
Condition
DPT pseudotime
DPT pseudotime
ROS pathway activity
A
CellANOVA
Control
T1D
UMAP1
UMAP2
T1D - Control = 0.176
p = 1.73e-169
Spearman r = -0.03
p = 2.7e-02
B
scDisInFact
T1D - Control = 0.175
p = 2.41e-114
Spearman r = -0.21
p = 4.3e-45
C
scVI
T1D - Control = 0.176
p = 6.83e-130
Spearman r = 0.77
p < 1e-300
D
scGen
T1D - Control = -0.072
p = 1.83e-21
Spearman r = 0.04
p = 3.9e-03
E
ComBat
T1D - Control = -0.085
p = 2.06e-84
Spearman r = 0.05
p = 9.7e-04
F
FastMNN
T1D - Control = -0.065
p = 7.25e-40
Spearman r = 0.27
p = 5.3e-73
G
Raw
T1D - Control = -0.062
p = 5.25e-29
Spearman r = 0.38
p = 4.7e-145
DPT pseudotime
ROS pathway activity score

References

1. A. P. Dempster, N. M. Laird, D. B. Rubin. Maximum Likelihood from Incomplete Data Via the EM Algorithm. *Journal of the Royal Statistical Society: Series B (Methodological)* **39**, 1-22 (1977). https://doi.org/10.1111/j.2517-6161.1977.tb01600.x
2. J. Sherman, W. J. Morrison. Adjustment of an Inverse Matrix Corresponding to a Change in One Element of a Given Matrix. *The Annals of Mathematical Statistics* **21**, 124-127 (1950). https://doi.org/10.1214/aoms/1177729893
3. M. D. Luecken, M. Buttner, K. Chaichoompu *et al.* Benchmarking atlas-level data integration in single-cell genomics. *Nat Methods* **19**, 41-50 (2022). https://doi.org/10.1038/s41592-021-01336-8
